# Site-Specific Protein Labeling and Generation of Defined Ubiquitin-Protein Conjugates Using an Asparaginyl Endopeptidase

**DOI:** 10.1021/jacs.2c02191

**Published:** 2022-07-18

**Authors:** Maximilian Fottner, Johannes Heimgärtner, Maximilian Gantz, Rahel Mühlhofer, Timon Nast-Kolb, Kathrin Lang

**Affiliations:** † Laboratory for Organic Chemistry (LOC), Department of Chemistry and Applied Biosciences (D-CHAB), ETH Zurich, Vladimir-Prelog-Weg 3, 8093 Zurich, Switzerland; ‡ Department of Chemistry, Technical University of Munich, Lichtenbergstr. 4, 85748 Garching, Germany; § Center for Protein Assemblies (CPA) and Lehrstuhl für Biophysik (E27), Physics Department, Technical University of Munich, Ernst-Otto-Fischer-Str. 8, 85748 Garching, Germany

## Abstract

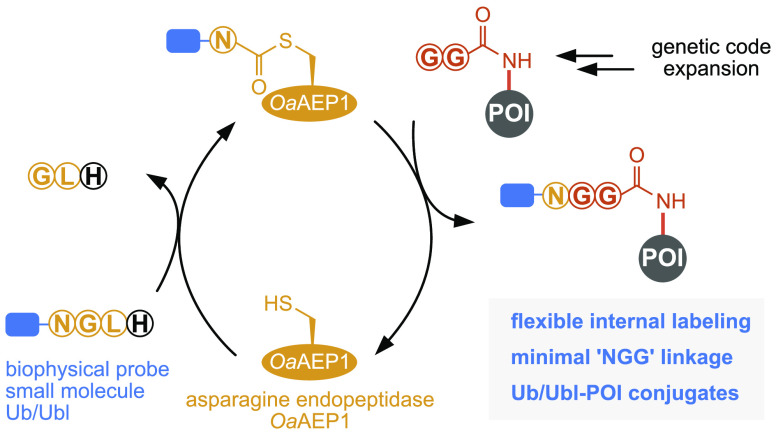

Asparaginyl endopeptidases (AEPs) have recently been widely utilized for peptide and protein modification. Labeling is however restricted to protein termini, severely limiting flexibility and scope in creating diverse conjugates as needed for therapeutic and diagnostic applications. Here, we use genetic code expansion to site-specifically modify target proteins with an isopeptide-linked glycylglycine moiety that serves as an acceptor nucleophile in AEP-mediated transpeptidation with various probes containing a tripeptidic recognition motif. Our approach allows simple and flexible labeling of recombinant proteins at any internal site and leaves a minimal, entirely peptidic footprint (NGG) in the conjugation product. We show site-specific labeling of diverse target proteins with various biophysical probes, including dual labeling at an internal site and the N-terminus. Furthermore, we harness AEP-mediated transpeptidation for generation of ubiquitin- and ubiquitin-like-modifier conjugates bearing a native isopeptide bond and only one point mutation in the linker region.

## Introduction

Site-specific protein labeling with various probes at internal sites and generation of defined protein–protein conjugates hold great promise for studying biological functions of proteins and for the development of therapeutic and diagnostic bioconjugates.^1−3^ Nevertheless, the modification of proteins at user-defined sites under mild conditions still represents a formidable challenge. Development of genetic code expansion approaches for site-specific cotranslational encoding of non-canonical amino acids (ncAAs) bearing bioorthogonal handles and their reaction with custom-made labels has enabled the generation of a diverse range of protein conjugates with an exquisite level of control over the labeling site and number of modifications.^4,5^ The synthesis and successful incorporation of functionalized ncAAs and probes can however be challenging. Furthermore, most bioorthogonal labeling reactions lead to bulky, hydrophobic, and artificial linkages between the biophysical probe and protein.^6−9^

Alternatively, chemoenzymatic labeling methods have proven to be powerful tools for attaching probes to specific amino acid side chains within recognition sequences under mild conditions.^10−12^ In a chemoenzymatic labeling experiment, the recombinantly expressed target protein is equipped with a peptide recognition motif (4–15 amino acids). The respective enzyme (a transferase^13^ or ligase^14,15^) binds to this recognition tag and catalyzes covalent attachment of a functionalized substrate to a specific amino acid within this motif, thereby labeling the protein of interest (POI). The recognition motifs are typically fused to the N- or C-terminus of the POI, allowing installation of modifications close to the target protein’s termini. In a few instances, it was also possible to introduce the recognition sequences into accessible loop regions of the POI, providing thereby greater flexibility with respect to the location and number of modifications.^11,16^ Still, most of these approaches leave sizable footprints and often bulky and artificial linkages in the ligation product.

Apart from ligases and transferases that covalently attach a functionalized probe to an amino acid within the recognition tag, also engineered proteases and transpeptidases [e.g., subtiligase,^17^ sortase,^18,19^ and asparaginyl endopeptidases (AEPs)^20^] have been used successfully for terminal protein labeling and for the generation of protein–protein conjugates linked via their N- or C-termini. Such enzymes typically cleave a peptide bond within a recognition motif fused to the POI, forming a labile enzyme-POI intermediate that undergoes specific transpeptidation with a peptide functionalized with a user-chosen probe.

**Figure 1:**
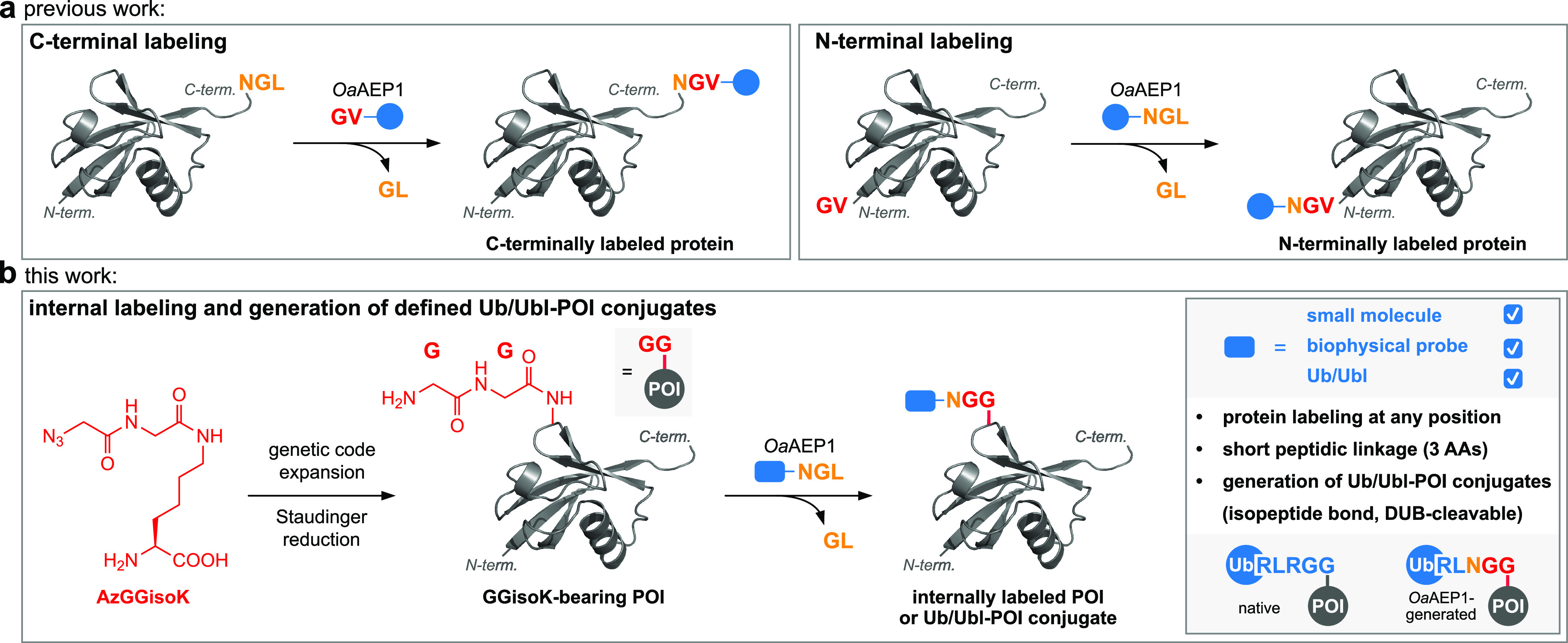
*Oa*AEP1-mediated protein labeling. (a) Previous work has established *Oa*AEP1-mediated labeling of protein C- and N-termini. C-terminal labeling is achieved through attachment of an NGL motif at the C-terminus of a POI and its *Oa*AEP1-catalyzed transpeptidation with a GV acceptor nucleophile bearing a user-defined cargo (blue circle). For N-terminal labeling, the GV acceptor nucleophile is installed at the N-terminus of the POI and reacted with a peptide displaying a C-terminal NGL motif in the presence of *Oa*AEP1. (b) *Oa*AEP1-mediated protein labeling at internal sites and generation of defined ubiquitin (Ub)/Ub-like protein (Ubl)-protein conjugates. Genetic code expansion allows site-specific encoding of GGisoK (via incorporation of AzGGisoK and subsequent Staudinger reduction) which can act as an acceptor nucleophile in *Oa*AEP1-driven transpeptidation with peptides or proteins bearing a C-terminal NGL motif. *Oa*AEP1-mediated internal site labeling enables the attachment of small molecules, biophysical probes, and Ub/Ubls to proteins in a site-specific manner, leaving a minimal scar (NGG). *Oa*AEP1 Ub/Ubl-POI conjugates display a native isopeptide bond and only one point mutation in the linker region.

Recent work has established the engineered AEP *Oa*AEP1 [C247A] (dubbed *Oa*AEP1 in the following) that can be produced recombinantly in *E. coli* as an ideally suited enzyme for N- and C-terminal labeling of recombinant proteins due to its high catalytic efficiency and minimal recognition motif (Figure 1a).^21,22^*Oa*AEP1 is a cysteine protease that cleaves C-terminally of an asparagine or aspartate residue within the tripeptidic NGL recognition motif, forming a thioester intermediate that is resolved by attack of a suitable nucleophile (e.g., α-amino group of an N-terminal amino acid) to yield a ligation product. *Oa*AEP1 has quite stringent sequence requirements for its recognition sequence (P1P1′P2′), with NGL representing an ideal motif, but was shown to be promiscuous for the incoming nucleophile sequence (P1″P2″). Previous work has found that both GL and GV dipeptides installed at the N-terminus of peptidic probes and proteins serve as efficient acceptor nucleophiles. Interestingly, the—in the latter case—resultant NGV ligation product is poorly recognized by *Oa*AEP1, shifting the reaction equilibrium toward the product and thereby allowing sequential dual labeling at the N- and C-terminus.^23^ Furthermore, *Oa*AEP1’s promiscuity for the incoming nucleophile was harnessed by ligating various primary amines—if presented at high enough molar excess (typically 500–1000 equiv)—to a C-terminal NGL recognition site.^24^ AEPs thereby represent versatile enzymes for protein labeling, but labeling is so far restricted to protein N- and C-termini.

On our quest to realize site-specific protein labeling at internal sites and to generate complex protein–protein conjugates that are not exclusively linked through their respective N- or C-termini, we here combine *Oa*AEP1-mediated transpeptidation with genetic code expansion. We show that target proteins carrying a site-specifically introduced isopeptide-linked glycylglycine moiety (GGisoK) serve as acceptor nucleophiles in *Oa*AEP1-mediated ligation with various NGL-bearing probes and proteins, allowing the generation of site-specific and user-defined protein conjugates displaying a minimal tripeptidic mark in the ligation product (Figure 1b). We modify diverse POIs at specific sites with different biophysical probes. Furthermore, we leverage *Oa*AEP1-mediated transpeptidation for the generation of defined ubiquitin (Ub)- and Ub-like protein (Ubl)-POI conjugates. Posttranslational modification of target proteins with Ub/Ubl presents one of the most common and versatile regulators in eukaryotic biology.^25,26^ During ubiquitylation, the C-terminal carboxylate of Ub is attached to the ε-amino group of a lysine within a substrate protein to form an isopeptide bond via a complex machinery employing E1/E2/E3 enzymes.^25,26^ We show that *Oa*AEP1 can be used to covalently attach a Ub/Ubl variant containing the NGL recognition motif in its C-terminus to a GGisoK-bearing POI. *Oa*AEP1-mediated Ub/Ubl conjugates display a native isopeptide bond connecting Ub/Ubl to a specific lysine and bear one point mutation in the linker region. Importantly, we show that these conjugates are still cleaved by the model deubiquitylase USP2,^27^ and we demonstrate the generality of our approach by preparing various site-specifically ubiquitylated substrate proteins (Ub, SUMO2, and histone H3).

## Results and Discussion

**Figure 2:**
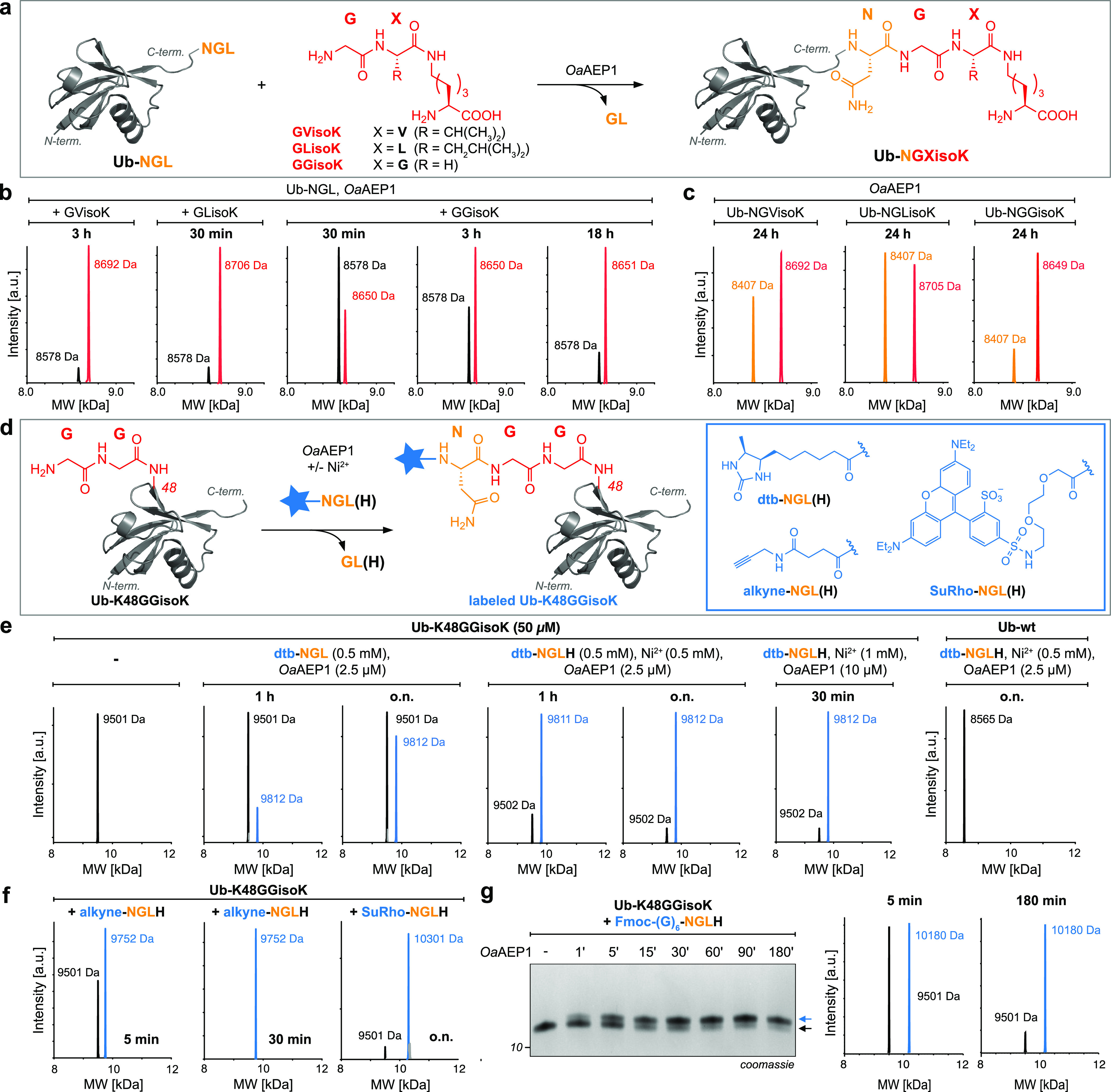
*Oa*AEP1-mediated protein labeling at internal sites. (a) Schematic representation of *Oa*AEP1-mediated transpeptidation between Ub-NGL and GXisoK peptides (X = V, L, or G). (b) LC–MS analysis shows >90% product formation within short times for GVisoK and GLisoK, while transpeptidation with GGisoK takes 18 h to reach approx. 85% yield (100 μM Ub-NGL, 1 mM GXisoK, 2.5 μM *Oa*AEP1, pH 7.0, 25 °C, calculated masses: Ub-NGL = 8579 Da, Ub-NGVisoK = 8693 Da, Ub-NGLisoK = 8707 Da, and Ub-NGGisoK = 8651 Da). (c) Time-resolved LC–MS analysis of purified Ub-NGXisoK ligation products (20 μM) incubated with *Oa*AEP1 (0.5 μM, pH 6.8, 30 °C) indicates superior stability of Ub-NGGisoK toward *Oa*AEP1-mediated hydrolysis (calculated masses: Ub-N = 8408 Da). (d) Schematic representation of *Oa*AEP1-mediated labeling of Ub-K48GGisoK with NGL(H) peptides bearing different biophysical probes [dtb-NGL(H), alkyne-NGLH, and SuRho-NGLH (SuRho = sulforhodamine), blue box]. (e) Optimization of *Oa*AEP1-mediated transpeptidation between Ub-K48GGisoK and dtb-NGL(H) as followed by time-resolved LC–MS. Although >90% labeling of Ub-K48GGisoK is achieved within 30 min using 1 mM dtb-NGLH, 1 mM NiSO_4_, and 10 μM *Oa*AEP1, Ub-wt is recalcitrant toward labeling under similar conditions (calculated masses: Ub-K48GGisoK-H6 = 9502 Da, Ub-K48(dtb-N)GGisoK-H6 = 9812, and Ub-wt = 8565 Da, o.n. = overnight). (f) LC–MS analysis of *Oa*AEP1-mediated transpeptidation of Ub-K48GGisoK with alkyne-NGLH and SuRho-NGLH peptides. Transpeptidation with alkyne-NGLH is quantitative within 30 min and with SuRho-NGLH yields >90% product formation overnight. (50 μM Ub-K48GGisoK, 1 mM alkyne/SuRho-NGLH, 1 mM NiCl_2_ or NiSO_4_, 5 or 10 μM *Oa*AEP1, pH 7.4, 30 °C; calculated masses: Ub-K48(alkyne-N)GGisoK-H6 = 9753 Da and Ub-K48(SuRho-N)GGisoK-H6 = 10302 Da). (g) SDS–PAGE (left) and LC–MS analysis (right) of *Oa*AEP1-driven labeling of Ub-K48GGisoK with Fmoc-(G)_6_-NGLH over time. The blue arrow indicates the labeled product displaying a gel shift, while a black arrow indicates the starting material. [50 μM Ub-K48GGisoK, 1 mM Fmoc-(G)_6_-NGLH, 1 mM NiCl_2_, 2 μM *Oa*AEP1, pH 6.8, 30 °C; calculated mass: Ub-K48(Fmoc-(G)_6_-N)GGisoK-H6 = 10181 Da]. For detailed conditions, see Figures S1–S8.

At the outset of our investigations, we were intrigued by the reported promiscuity of *Oa*AEP1 for nucleophile acceptors (P1″P2″) and we assessed the enzyme’s tolerance and specificity for nucleophiles that can be site-specifically incorporated into proteins in the form of ncAAs via genetic code expansion. We have recently shown that we can site-specifically modify any POI with a GG-dipeptide moiety attached to the ε-amino group of a lysine residue via genetic encoding of AzGGisoK and its phosphine-based reduction to GGisoK (Figure 1b).^28,29^ To assess *Oa*AEP1’s tolerance for GXisoK nucleophiles (with X representing different natural amino acids), we synthesized GLisoK, GVisoK, and GGisoK and tested their *Oa*AEP1-mediated transpeptidation onto Ub bearing the recognition sequence NGL at its C-terminus (Ub-NGL, Figure 2a). GLisoK and GVisoK were efficiently attached onto Ub-NGL within 30 min using 10 mol equiv of acceptor nucleophile over Ub-NGL (100 μM) and 0.02 equiv of *Oa*AEP1. Ligation using GGisoK also resulted in the correct ligation product but was considerably slower, leading to >85% Ub-NGGisoK formation after 18 h (Figures 2b and S1). Unsurprisingly though, this ligation product showed higher stability against enzymatic hydrolysis upon prolonged incubation with *Oa*AEP1 in the absence of any other acceptor nucleophile, with >85% intact NGG-ligation product after 24 h, while NGL- and NGV-bearing ligation products showed ∼50% hydrolysis under the same conditions (Figures 2c and S2). Encouraged by these results, we set out to investigate if GGisoK-bearing proteins could also function as potential nucleophiles in *Oa*AEP1-mediated transpeptidation. We site-specifically encoded AzGGisoK in response to an introduced amber codon using the previously reported, selective pyrrolysyl-tRNA synthetase-derived AzGGisoKRS/tRNA_CUA_ pair.^28^ The azide moiety in AzGGisoK-modified proteins can easily be reduced to its amine analogue via Staudinger reduction using phosphines such as tris(2-carboxyethyl)phosphine or 2-(diphenylphosphino)-benzoic acid, generating GGisoK-bearing proteins. Using this approach, we expressed C-terminally H6-tagged Ub, carrying GGisoK at position K48 (Ub-K48GGisoK). In parallel, we synthesized a desthiobiotin-NGL probe (dtb-NGL, Figure 2d). Incubation of 50 μM Ub-K48GGisoK with 500 μM dtb-NGL in the presence of 0.05 mol equiv *Oa*AEP1 overnight however only afforded roughly 50% of the labeled product, as assessed by liquid chromatography mass spectrometry (LC–MS), with the remaining 50% constituting unlabeled Ub-K48GGisoK (Figure 2e).

We reckoned that the sluggish reaction progress may stem from the fact that—contrary to traditional labeling approaches—in our approach, the nucleophile-bearing POI (Ub-K48GGisoK) is used as the substoichiometric component with the recognition motif-containing NGL probe in 10-fold molar excess. As the GL leaving group is released from the recognition motif over the course of the reaction, this byproduct competes with the desired GGisoK nucleophile, stagnating in a product-limiting equilibrium. Inspired by previous work,^30,31^ we extended the NGL recognition motif in dtb-NGL by a C-terminal histidine (dtb-NGLH). Thereby, the released GLH can be quenched by Ni^2+^ complexation, sequestering the competing nucleophile acceptor (Figure S3). Using dtb-NGLH in the presence of 500 μM NiSO_4_, >80% of labeled protein was observed within 1 h at 30 °C. Increasing the *Oa*AEP1 concentration to 10 μM and/or using 20-fold excess of the NGLH peptide in combination with 1 mM Ni^2+^ led to ∼95% product formation in less than an hour with 50 μM GGisoK-bearing protein (Figures 2e and S3–S5). Importantly, for wild-type Ub (Ub-wt), lacking the GGisoK nucleophile, we could not observe any ligation product formation (Figures 2e, S3 and S4). To show that labeling progress can indeed be accurately followed by quantifying MS-peak intensities, we purified dtb-labeled Ub to homogeneity via Strep-Tag affinity chromatography and determined MS-peak intensities of various protein mixtures, confirming a linear correlation between protein concentration and MS-peak intensity (Figure S6). Having established near-quantitative labeling conditions, we next explored the chemical diversity tolerated on NGLH probes for varied protein labeling. Both alkyne- and fluorophore-NGLH probes allowed efficient labeling of GGisoK-bearing POIs. Furthermore, we synthesized an NGLH-bearing decapeptide that allowed us to follow protein modification progress via migration differences of unlabeled and labeled protein on SDS-PAGE (Figures 2f,g and S6–S8).

**Figure 3:**
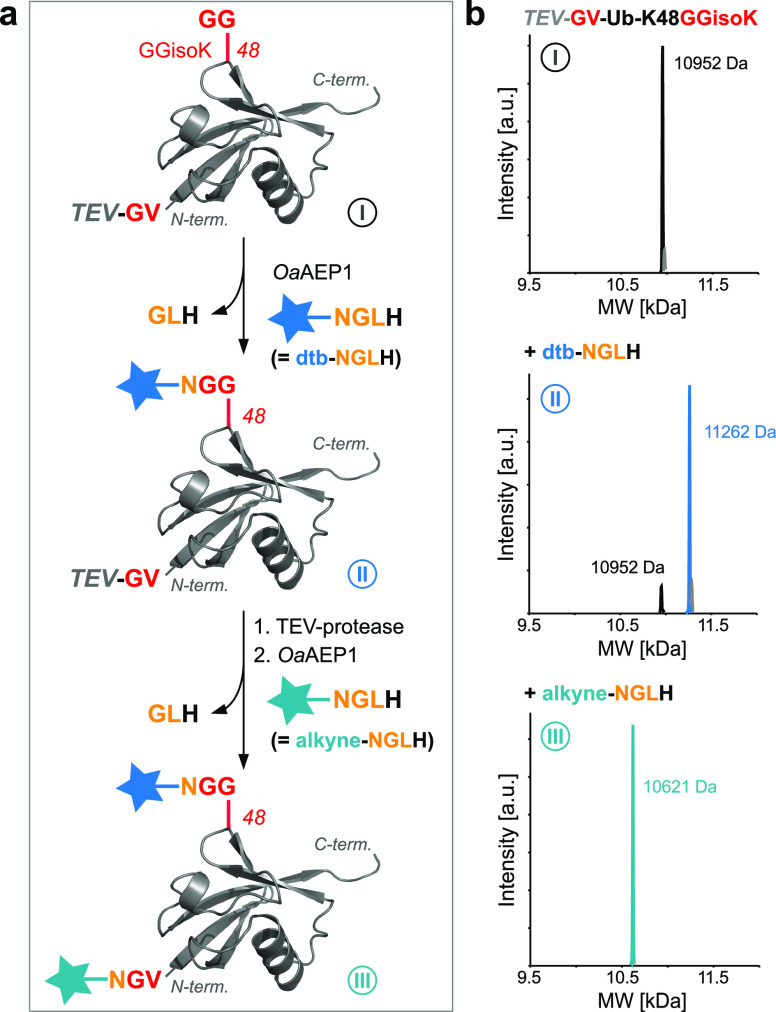
Site-specific sequential dual labeling using *Oa*AEP1. (a) Schematic representation of *Oa*AEP1-mediated dual labeling of TEV-GV-Ub-K48GGisoK (I). In the first step, the GGisoK moiety is labeled with dtb-NGLH (II) (50 μM (I), 1 mM dtb-NGLH, 2 μM *Oa*AEP1, 500 μM NiCl_2_, pH 6.8, 30 °C, 100 min). Subsequent TEV cleavage reveals the N-terminal GV moiety which is modified with alkyne-NGLH (III) (50 μM (II), 1 mM alkyne-NGLH, 2 μM *Oa*AEP1, 500 μM NiCl_2_, pH 6.8, 30 °C, 150 min). (b) LC–MS analysis of different labeling steps as depicted in (a). (Calculated masses: TEV-GV-Ub-K48GGisoK-H6 = 10952 Da, TEV-GV-Ub-K48(dtb-N)GGisoK-H6 = 11262 Da, and alkyne-NGV-Ub-K48(dtb-N)GGisoK-H6 = 10621 Da). For detailed conditions, see Figure S9.

As the NGG motif that is installed upon transpeptidation is essentially refractory toward *Oa*AEP1-mediated hydrolysis, we next explored site-specific sequential protein dual labeling at internal sites and at the N-terminus using differently functionalized NGLH-probes. Therefore, we expressed a Ub-variant bearing GGisoK at position K48 and displaying a TEV recognition site at its N-terminus, followed by a GV motif (Figure 3a). In the first step, we labeled the internal GGisoK with dtb-NGLH using *Oa*AEP1. Next, we exposed the N-terminal GV acceptor nucleophile by TEV protease cleavage to make it amenable to transpeptidation with *Oa*AEP1 using an alkyne-NGLH probe. Near-quantitative labeling was ascertained by LC–MS for each step (Figures 3b and S9). Additionally, we showed that the alkyne moiety could be efficiently used in further Cu(I)-catalyzed alkyne–azide cycloaddition for functionalization with commercially available azide-bearing fluorophores (Figure S9).

**Figure 4:**
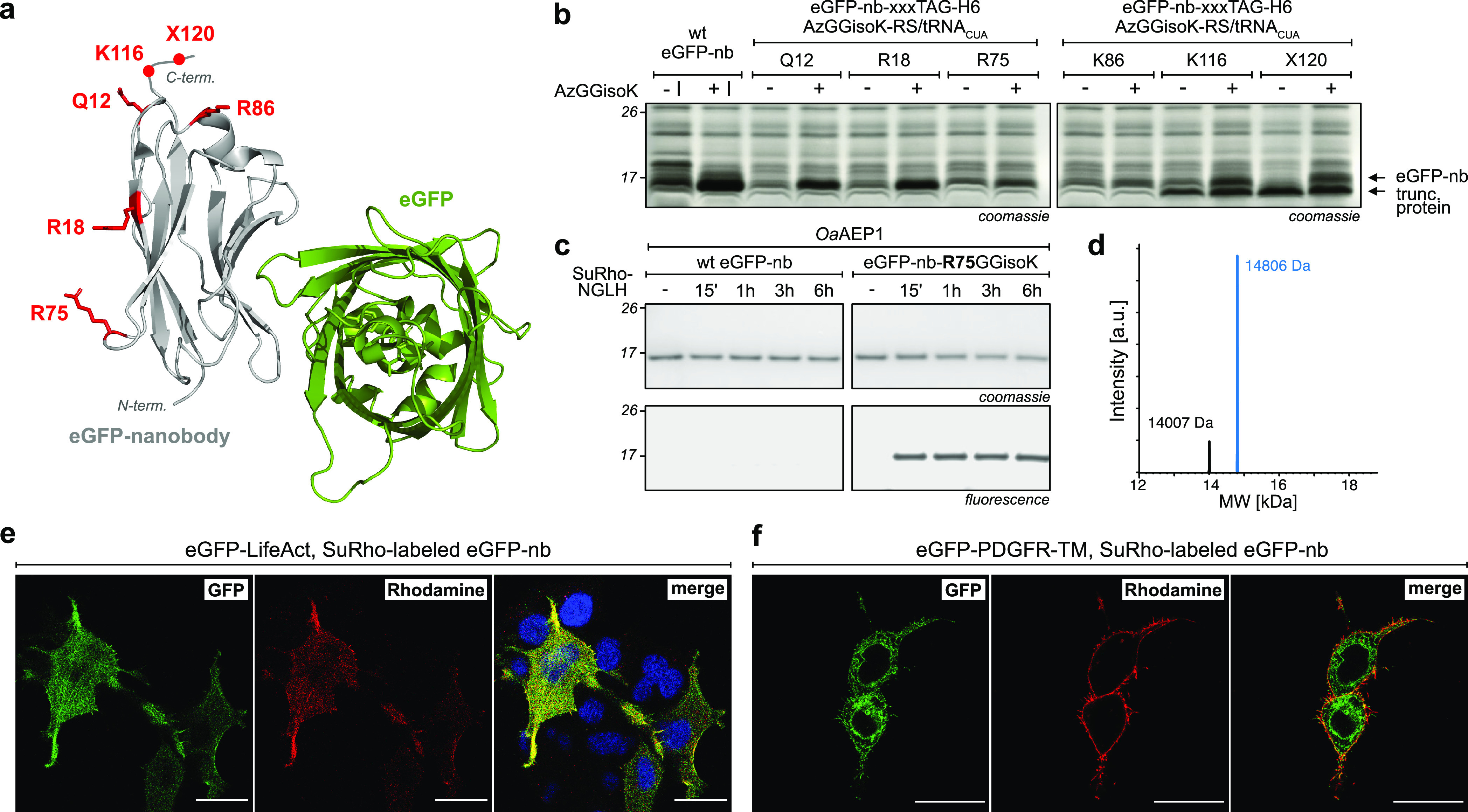
*Oa*AEP1-mediated functionalization of the eGFP nanobody (eGFP-nb). (a) Crystal structure of the eGFP-nb bound to eGFP (PDB: 3OGO). Individual positions chosen for the site-specific introduction of GGisoK are marked in red. (b) SDS–PAGE analysis of eGFP-nb expressions (−/+ I = without/with induction). (c) Coomassie staining and in-gel fluorescence of *Oa*AEP1-mediated site-specific labeling of eGFP-nb-R75GGisoK with SuRho-NGLH shows specific labeling for GGisoK bearing eGFP-nb (30 μM eGFP-nb-R75GGisoK, 0.6 mM SuRho-NGLH, 5 μM *Oa*AEP1, 1 mM NiSO_4_, pH 7.4, 30 °C). (d) LC–MS analysis of purified eGFP-nb-R75(SuRho-N)GGisoK shows >85% labeling yield (calculated masses: eGFP-nb-R75GGisoK-H6 = 14,009 Da and eGFP-nb-R75(SuRho-N)GGisoK-H6 = 14809 Da). (e) Fluorescence microscopy of fixed HEK293T cells overexpressing eGFP-LifeAct treated with eGFP-nb-R75(SuRho-N)GGisoK. (f) Live-cell microscopy of HEK293T cells overexpressing eGFP-PDGFR-TM treated with eGFP-nb-R75(SuRho-N)GGisoK. Scale bars correspond to 20 μm. For detailed conditions and controls, see Figures S10 and S13.

To demonstrate that GGisoK-directed transpeptidation is suited for the preparative generation of defined POI-small-molecule conjugates, we aimed at site-specifically labeling the anti-eGFP nanobody (eGFP-nb) and test its specific binding to eGFP expressed in mammalian cells.^32^ Guided by the eGFP-nb:eGFP crystal structure (Figure 4a, PDB: 3OGO),^33^ we selected various surface-exposed positions within eGFP-nb to be replaced by GGisoK. For this, we expressed and purified eGFP-nb variants bearing GGisoK at individual positions (Q12, R18, R75, K86, K116, and at position 120 within an artificially prolonged C-terminus) in yields from 1 to 8 mg/L culture (Figures 4b and S10). Having confirmed via in vitro pull-down assays that all GGisoK-bearing eGFP-nb variants retained their binding capability toward eGFP (Figure S10), we proceeded with *Oa*AEP1-mediated labeling using a sulforhodamine-NGLH probe (SuRho-NGLH, Figures 2f, S8 and S11). While we observed specific labeling for all six GGisoK-bearing nanobody variants as judged by in-gel fluorescence, the labeling efficiencies varied greatly among the six variants, presumably due to disparate steric access of *Oa*AEP1 to the GGisoK acceptor nucleophile. eGFP-nb-R75GGisoK showed >85% labeling within 3 h, as assessed by LC–MS, and was purified at preparative scale for further experiments (Figures 4c,d and S11).

We benchmarked this site-specifically SuRho-labeled eGFP-nb for imaging of eGFP-expressing proteins both in fixed and live mammalian cells. For this, we transfected HEK293T cells with eGFP-LifeAct, a fusion between eGFP and a 17-amino-acid-long peptide that binds specifically to the actin cytoskeleton.^34^ Cells were fixed and incubated with SuRho-labeled eGFP-nb. Efficient labeling of the cytoskeleton and colocalized green and red fluorescence was confirmed by fluorescence microscopy (Figure 4e). Importantly, eGFP-LifeAct-expressing cells treated with the SuRho-NGLH probe alone or with unlabeled eGFP-nb did not show any nonspecific labeling (Figure S12). To show membrane labeling on live cells, we transfected HEK293T cells with a pDisplay construct where eGFP is fused to the transmembrane domain of the platelet-derived growth factor receptor (eGFP-PDGFR-TM) to display eGFP on the cell surface.^35^ Incubation of eGFP-PDGFR-TM-expressing cells with SuRho-labeled eGFP-nb led to specific labeling of cells expressing eGFP at their plasma membrane (Figure 4f), while cells treated with SuRho or eGFP-nb alone did not show any nonspecific labeling (Figure S13).

**Figure 5:**
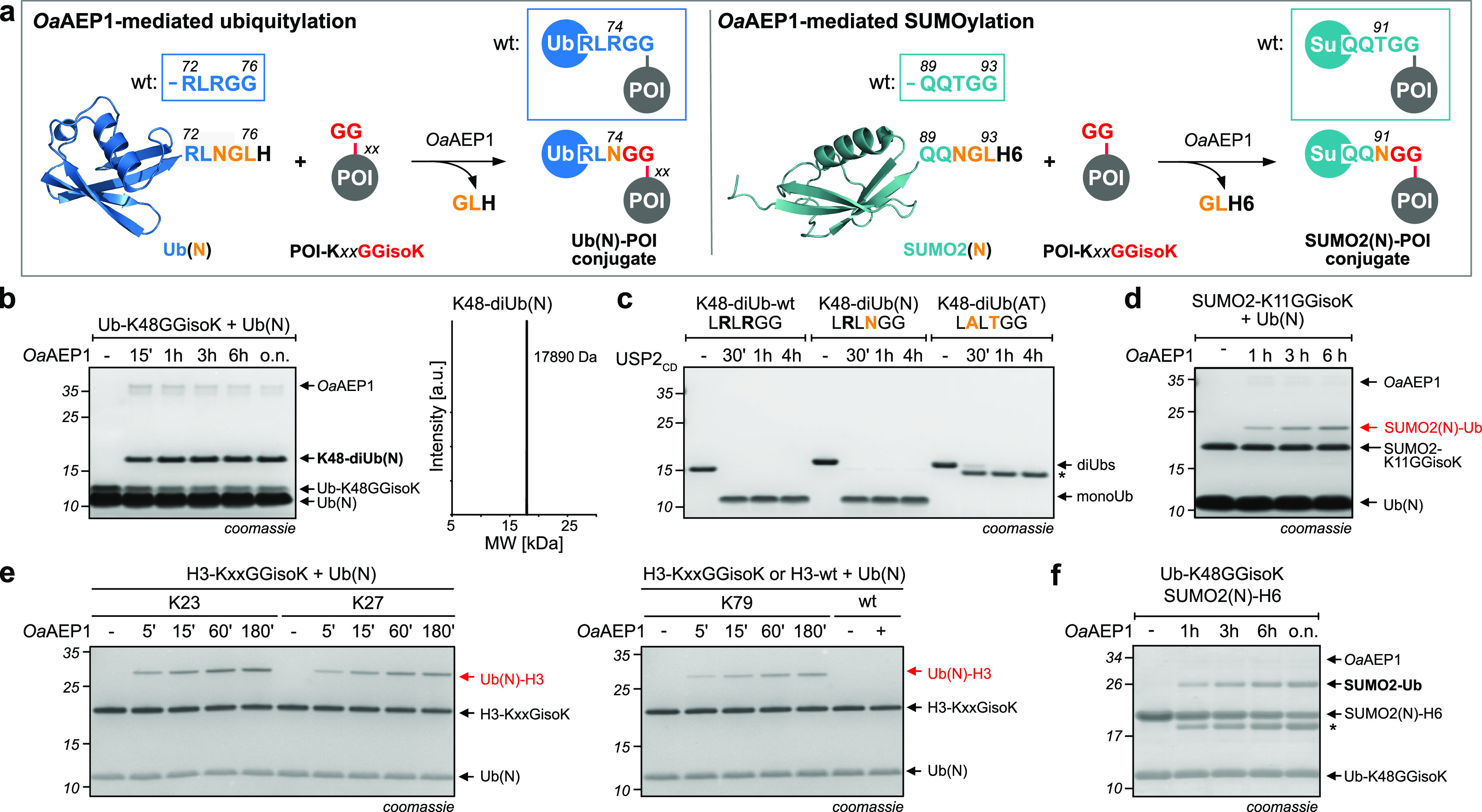
Site-specific monoubiquitylation and monoSUMOylation using *Oa*AEP1. (a) Schematic representation of *Oa*AEP1-mediated site-specific monoubiquitylation/SUMOylation. Transpeptidation between Ub displaying an NGLH motif at its C-terminus [Ub(N)] and a GGisoK-bearing target protein results in the formation of an isopeptide-linked Ub-POI conjugate bearing only a single point mutation in the flexible linker region (R74N). In a similar fashion, SUMO2(N), bearing a T91N mutation, can be attached to a substrate protein displaying a GGisoK moiety using *Oa*AEP1. (b) SDS–PAGE analysis showing *Oa*AEP1-mediated K48-diUb formation by incubation of Ub-K48GGisoK, Ub(N), and *Oa*AEP1 [left, 50 μM Ub-K48GGisoK, 250 μM Ub(N), 2 μM *Oa*AEP1, 500 μM NiCl_2_, pH 6.8, 37 °C]. LC–MS analysis of purified K48-diUb(N) [right, calculated mass: K48-diUb(N) = 17892 Da]. (c) SDS–PAGE analysis of deubiquitylation assays with USP2_CD_. Natively linked K48-diUb (K48-diUb-wt) and *Oa*AEP1-generated K48-diUb(N) are hydrolyzed within 30 min, while Srt2A-generated K48-diUb(AT) is recalcitrant toward DUB hydrolysis (10 μM K48-diUbs and 200 nM USP2_CD_). *denotes cleavage of the C-terminal H6-tag of the acceptor Ub of K48-diUb(AT). (d) SDS–PAGE analysis showing *Oa*AEP1-mediated ubiquitylation of SUMO2-K11GGisoK-H_6_ by incubation of SUMO2-K11GGisoK, Ub(N), and *Oa*AEP1 (50 μM SUMO2-K11GGisoK, 250 μM Ub(N), 2 μM *Oa*AEP1, pH 6.8, 37 °C). (e) SDS–PAGE analysis showing *Oa*AEP1-mediated ubiquitylation of histone H3 by incubation of H3-KxxGGisoK, Ub(N), and *Oa*AEP1 (20 μM H3-KxxGGisoK-H_6_, 150 μM Ub(N), 1 μM *Oa*AEP1, pH 7.0, 25 °C). (f) SDS–PAGE analysis of *Oa*AEP1-mediated SUMOylation of Ub-K48GGisoK (150 μM Ub-K48GGisoK, 150 μM SUMO2(N), 2 μM *Oa*AEP1, pH 7.4, 25 °C) *denotes hydrolysis of the C-terminal *Oa*AEP1 motif leading to cleavage of the H6-tag of SUMO2(N)-H6. For detailed conditions, see Figures S14 and S15.

Given that *Oa*AEP1 transpeptidation using substrates with NGL as a recognition motif and GGisoK-bearing proteins as acceptor nucleophiles leads to ligation products with an NGG sequence, we hypothesized that we could use this approach to build Ub-POI conjugates bearing a native isopeptide bond and harboring only one point mutation in the Ub C-terminus. Ub is a small, globular, and highly conserved 76 amino acid protein.^25,26^ Its C-terminus is unstructured, and the last six amino acids have the sequence_71_-LRLRGG. During ubiquitylation, the carboxylate of G76 is attached to specific lysine residues in a POI (or Ub itself) through a complex enzymatic machinery, forming an isopeptide bond. We have recently developed a chemoenzymatic approach to build ubiquitylated proteins using the transpeptidase sortase (sortylation).^28,29^ For this, we mutated the Ub C-terminus to contain a sortase (Srt2A)^36^ recognition motif [_71_-LALTGG, dubbed Ub(AT)]. Srt2A-mediated transpeptidation between Ub(AT) and a GGisoK-bearing POI leads to Ub-POI conjugates displaying a native isopeptide bond and two point mutations in the linker region (R72A and R74T). We were able to show that sortase-generated diUbs largely maintain structural and functional integrity by retaining their binding affinities to many Ub-binding domains, a requirement for decoding diverse cellular functions. Nevertheless, the two point mutations make sortase-generated diUbs resistant toward DUBs, indicating that their recognition by DUBs might be impaired. In order to build more native diUbs, we expressed Ub bearing an RLNGLH sequence at its C-terminus [_72_-RLNGLH, dubbed Ub(N), Figure 5a]. Incubation of Ub-K48GGisoK (50 μM) with a fivefold excess of Ub(N) in the presence of 0.04 equiv of *Oa*AEP1 afforded K48-linked diUb within 15 min, bearing a native isopeptide bond and one point mutation R74N [K48-diUb(N), Figures 5b and S14]. K48-diUb(N) could be produced in mg yields, and its identity was confirmed by LC–MS. We next set out to test if the isopeptide bond in K48-diUb(N) would be cleaved by DUBs. We therefore incubated purified K48-diUb-wt, Srt2A-generated diUb [K48-diUb(AT)], and *Oa*AEP1-generated diUb [K48-diUb(N)] with the catalytic domain of Ub carboxyl terminal hydrolase 2 (USP2_CD_). Although the Srt2A-generated K48-diUb(AT) is completely resistant to USP2_CD_ cleavage, K48-diUb(N) is as efficiently cleaved to the corresponding monoUbs as observed for K48-diUb bearing the wt sequence in the linker region (Figures 5c and S14).

To prove the generality of *Oa*AEP1-mediated ubiquitylation, we showed that other natively ubiquitylated substrate proteins can also be accessed by *Oa*AEP1 and Ub(N). We prepared SUMO2 displaying GGisoK at position K11, as K11 represents a well-known and the most abundant Ub linkage site on SUMO2.^37^ Incubation of SUMO2-K11GGisoK with Ub(N) in the presence of *Oa*AEP1 led to specific formation of the SUMO2-Ub conjugate (Figure 5d), while SUMO2 bearing *tert* butyloxycarbonyl-l-lysine (BocK) at position K11 did not generate any SUMO2-Ub conjugate (Figure S14). Among the most abundant monoubiquitylated proteins are histones H2A and H2B with ubiquitylation fulfilling critical roles in regulating transcription and other cellular processes.^38−40^ Recent reports have shown that also histones H3 and H4 are ubiquitylated,^41^ for example, various large-scale quantitative proteomics studies identified several ubiquitylation sites within H3,^42^ but the functions of these modifications are less understood. To demonstrate that *Oa*AEP1-mediated ubiquitylation is suited to generate defined Ub-H3 conjugates, we prepared H3-constructs bearing amber codons at nine lysine positions and incorporated AzGGK in response to these introduced amber codons (Figure S15). After reduction of the azide group in AzGGK, we purified H3-K23GGisoK, H3-K27GGisoK, and H3-K79GGisoK via affinity chromatography and cation exchange followed by refolding. All three H3-GGisoK variants were incubated with Ub(N) and *Oa*AEP1, and specific formation of Ub-H3 conjugates was observed within 5 min. Ubiquitylation yields ranged between 31 und 35% (Figures 5e and S15).

We envisioned that *Oa*AEP1-mediated generation of Ub conjugates may also be extendable to other Ubls that share Ub’s common ß-grasp fold with a flexible six-residue C-terminal tail and the characteristic GG motif that is attached to a lysine residue in the target protein. We introduced the NGL recognition motif into the C-terminus of the small-Ub-like-modifier SUMO2^43^ by introducing the point mutation T91N, generating SUMO2(N) with the C-terminal sequence _89_-QQNGLH_6_ (Figure 5f). Gratifyingly, incubation of SUMO2(N) and Ub-K48GGisoK in the presence of *Oa*AEP1 led to the formation of the expected SUMO2-Ub conjugate bearing a T91N mutation in its linker region, confirming that *Oa*AEP1-mediated transpeptidation is transferable to other Ubls (Figures 5d and S14).

## Conclusions

We have shown that the recently engineered and chemically improved AEP *Oa*AEP1 allows site-specific internal labeling of GGisoK-bearing proteins with a variety of small molecules and biophysical probes. *Oa*AEP1-mediated labeling can be conducted at physiological pH in mild and aqueous buffers and leads to near-quantitative and essentially irreversible formation of the ligation product in the presence of Ni^2+^ salts. *Oa*AEP1-mediated labeling compares favorably to other chemoenzymatic labeling approaches in terms of size and nature of recognition tag and footprint left in the ligation product. It requires an NGL recognition motif in the labeling reagent and the site-specific incorporation of GGisoK into a target protein, leading to a flexible and minimal tripeptidic NGG motif in the linker between the label and POI in the conjugation product. This is in contrast to other enzymatic labeling approaches such as transferases (e.g., transglutaminase,^44^ SUMO-conjugating enzyme Ubc9,^16^ phosphopanthetheinyl transferase,^13^ and phosphocholine transferase^45^) and ligases (e.g., biotin ligase^14^ and lipoic acid ligase^15^) that typically leave a mark of at least 6–15 amino acids in their conjugation products and often result in bulky and artificial linkages. Importantly, *Oa*AEP1 only requires stable peptidic probes that can be easily synthesized and/or are often commercially available and results in entirely peptidic linkers, which may be especially advantageous for generating antibody-drug-like conjugates with reduced immunogenicity for in vivo administration. As the resulting NGG motif in the ligation product is essentially resistant toward further *Oa*AEP1 hydrolysis, our approach is also suitable for sequential dual labeling at both an internal site and the N-terminus with diverse probes. Additionally, only low concentrations of *Oa*AEP1 (0.01–0.04 mol equivalents) are required for efficient transpeptidation, as opposed to, for example, sortase-mediated labeling^28^ and Ubc9-mediated labeling,^16^ which mostly rely on >0.2 mol equivalents of their corresponding enzymes for high conversion rates. *Oa*AEP1-mediated transpeptidation in combination with site-specific incorporation of GGisoK via genetic code expansion therefore represents an operationally simple process for labeling of recombinant proteins at internal sites, leaving a minimal, entirely peptidic scar (NGG) in the ligation product. It is however worth noting that due to the small recognition motif NGL, unwanted cleavage within the target protein may be a side reaction that can occur during *Oa*AEP1 transpeptidation, especially if the POI displays asparagine residues followed by small and hydrophobic amino acids in highly exposed loop regions.

Importantly, *Oa*AEP1-mediated labeling can also be harnessed for conjugation of folded proteins to generate novel architectures and protein–protein conjugates in a programmable manner. On our quest to generate and study distinct Ub/Ubl topologies, we were able to show that *Oa*AEP1-mediated transpeptidation allows site-specific monoubiquitylation and monoSUMOylation of target proteins. We have shown the ubiquitylation of diverse target proteins including the site-specific covalent attachment of Ub to SUMO2 and histones using *Oa*AEP1. *Oa*AEP1-generated Ub-POI conjugates display a native isopeptide bond connecting Ub and the POI with only one point mutation in the Ub/Ubl C-terminus. Compared to our previously developed sortylation approach,^28,29^*Oa*AEP1-mediated site-specific conjugation of Ub/Ubl to target proteins requires less enzyme and shows faster conversion to product. For sortase-mediated ubiquitylation, a leucine spacer amino acid is often introduced preceding the sortase recognition motif, as this increases accessibility of the sortase to the Ub C-terminus and therefore sortylation yields.^28^ Interestingly, introduction of such a spacer amino acid was not needed for *Oa*AEP1-mediated ubiquitylation and SUMOylation. Excitingly, this leads to Ub/Ubl-POI conjugates that contain only one single point mutation in the linker region between Ub/Ubl and the POI. In fact—in contrast to sortase-generated diUbs—*Oa*AEP1-generated diUbs are cleaved by DUBs to a similar extent as diUbs bearing a wt-linker, confirming their functional and structural resemblance to endogenous Ub/Ubl-conjugates. In this sense, *Oa*AEP1-mediated ubiquitylation complements the approach recently reported by Bode and co-workers that requires the introduction of up to three point mutations into the POI to make it a substrate for Ubc9-mediated ubiquitylation.^16,46^ Ultimately, we envision that *Oa*AEP1-mediated generation of Ub and Ubl conjugates may be combined with sortylation to further extend our recently developed approach *Ubl-tools* (Ubl-topologies via orthogonal sortylation)^29^ as a modular and robust tool for accessing defined Ub/Ubl chains where we can place DUB-resistant and DUB-susceptible linkages at defined positions.

## Supporting Information Available

Figures supporting additional experiments, full gels and western blots, experimental procedures, plasmid sequences, and amino acid sequences of used proteins (PDF)
