# Supplementary material for: Site-Specific Protein Labeling and Generation of Defined Ubiquitin-Protein Conjugates Using an Asparaginyl Endopeptidase
Source: J Am Chem Soc. 2022 Jul 18;144(29):13118–26. doi: 10.1021/jacs.2c02191 (PMC9335880; doi:10.1021/jacs.2c02191)
Supplement: Supplementary file 1 — ja2c02191_si_001.pdf [file ja2c02191_si_001.pdf]

Supplementary Information

Site-Specific Protein Labeling and Generation of Defined Ubiquitin-Protein Conjugates Using an Asparaginyl Endopeptidase

Maximilian Fottner,[1] Johannes Heimgärtner,[1,‡] Maximilian Gantz,[2,‡] Rahel Mühlhofer,[2,‡] Timon Nast-Kolb[3] and Kathrin Lang[1,2]*

* to whom correspondence should be addressed: kathrin.lang@org.chem.ethz.ch

[1]Laboratory for Organic Chemistry (LOC), Department of Chemistry and Applied Biosciences (D-CHAB), ETH Zurich, Vladimir-Prelog-Weg 3, 8093 Zurich, Switzerland

[2]Department of Chemistry, Technical University of Munich, Lichtenbergstr. 4, 85748 Garching, Germany

[3]Center for Protein Assemblies (CPA) and Lehrstuhl für Biophysik (E27), Physics Department, Technical University of Munich, Ernst-Otto-Fischer-Str. 8, 85748 Garching, Germany

[‡] these authors contributed equally to this work.

TABLE of CONTENT

Supplementary Figures S1-S15 (pages 3-19)

Supplementary Tables S1-S3 (pages 20-24)

Supplementary Notes (pages 25-28)

Experimental procedures (pages 29-49)

1. **General methods: Plasmids and reagents (page 29)**
2. **Chemical synthesis (pages 30-35)**
3. **Protein expression and purification (pages 36-42)**
4. ***Oa*AEP1 substrate scope and reversibility assays (page 42)**
5. ***Oa*AEP1-mediated labeling of GGisoK-bearing proteins (pages 42-45)**
6. ***Oa*AEP1-mediated Ublyation of GGisoK-bearing proteins (pages 45-46)**
7. **Preparation of K48-diUbs and DUB assays (pages 46-47)**
8. **Pull-down assays (pages 47-48)**
9. **Mammalian cell culture (page 48)**
10. **Confocal microscopy (page 49)**
11. **LC-MS (page 49)**

Author contributions (page 50)

References (page 50)

Supplementary Figures

Supplementary Figure S1

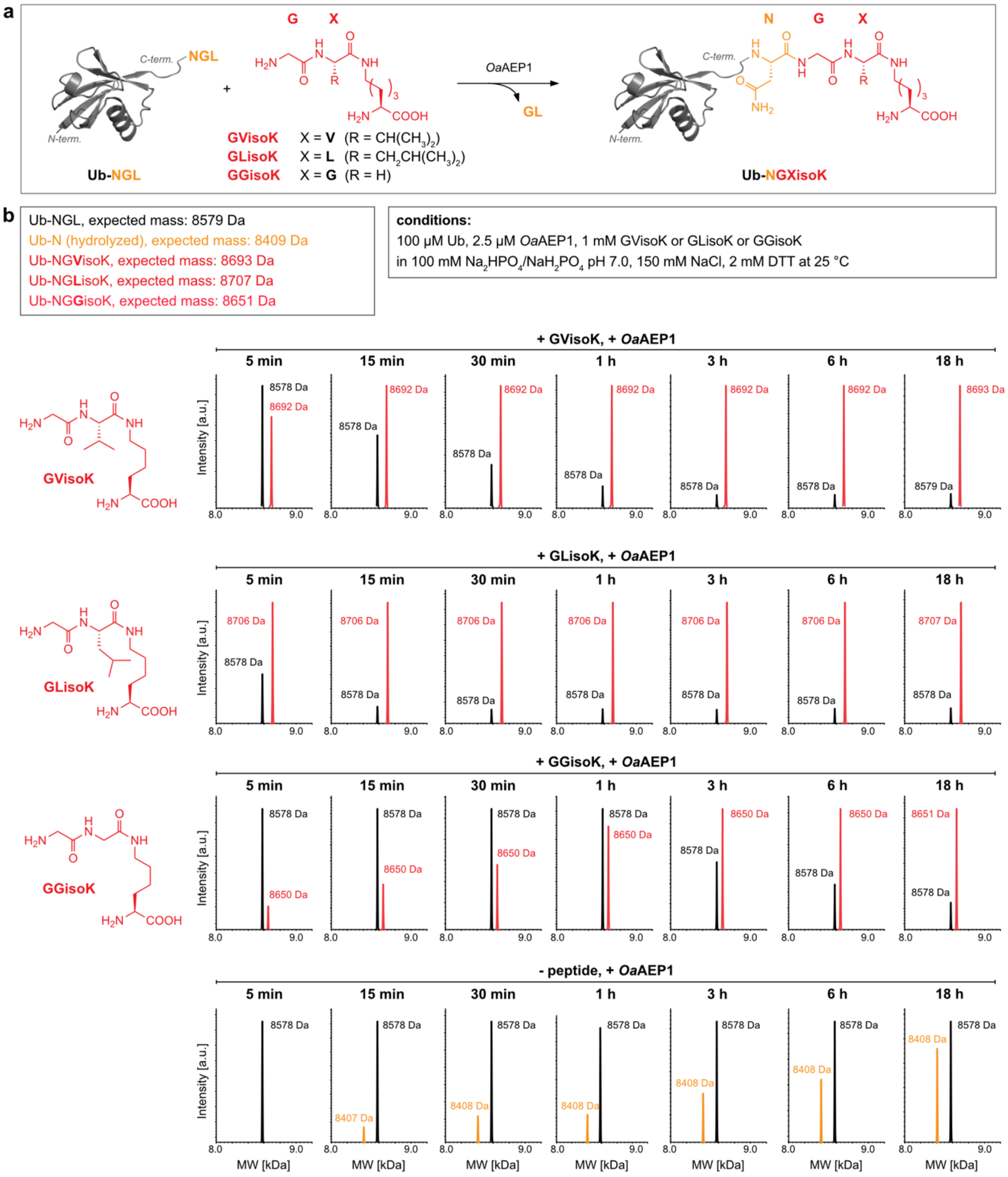

Supplementary Figure S1. Evaluation of different acceptor nucleophiles for *Oa*AEP1-mediated transpeptidation. **a)** Schematic representation of *Oa*AEP1-mediated transpeptidation between ubiquitin (Ub) bearing an NGL motif at its C-terminus and GXisoK peptides (with X being V, L or G). **b)** Time-resolved LC-MS analysis of the transpeptidation reactions between Ub-NGL and GVisoK, GLisoK or GGisoK, as well as a control reaction without acceptor nucleophile. Results show that GLisoK is the best acceptor nucleophile with approx. 90 % conversion after 15 min followed by GVisoK (approx. 90 % after 3 h) and GGisoK (approx. 85 % after 18 h). In absence of acceptor nucleophile *Oa*AEP1-mediated hydrolysis of the NGL motif can be observed.

Supplementary Figure S2

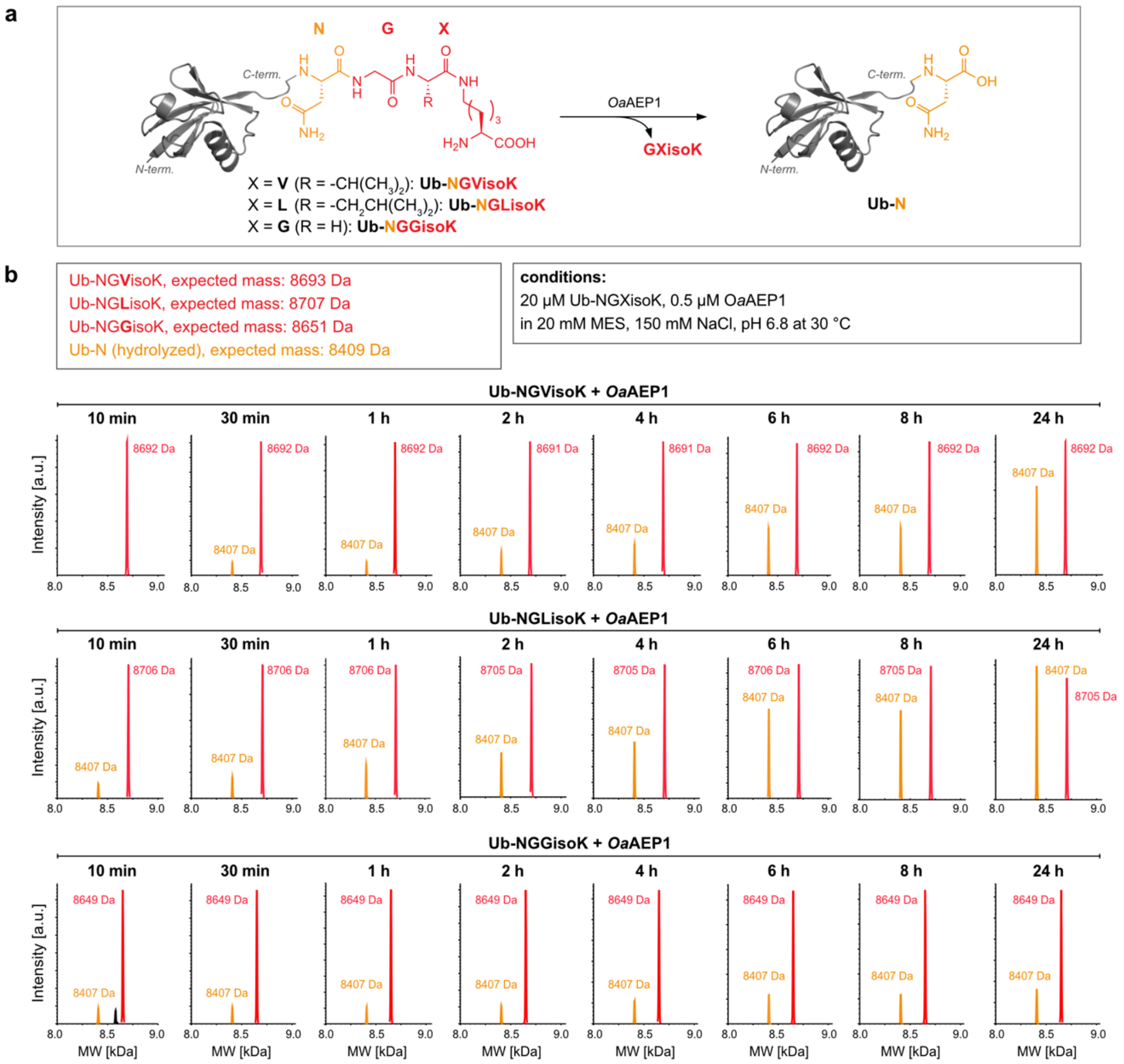

Supplementary Figure S2. Evaluation of *Oa*AEP1-generated conjugate stability against enzymatic hydrolysis. **a)** Schematic representation of *Oa*AEP1-mediated hydrolysis of Ub-NGXisoK conjugates (with X being V, L or G). **b)** Time-resolved LC-MS analysis of the *Oa*AEP1-mediated hydrolysis of Ub-NGXisoK conjugates. Results show that Ub-NGLisoK is the least stable conjugate with >50 % hydrolysis after 24 h of incubation, followed by Ub-NGVisoK (approx. 40 % hydrolysis after 24 h). Ub-NGGisoK is the most stable conjugate showing only minor hydrolysis (approx. 15 % after 24 h).

Supplementary Figure S3

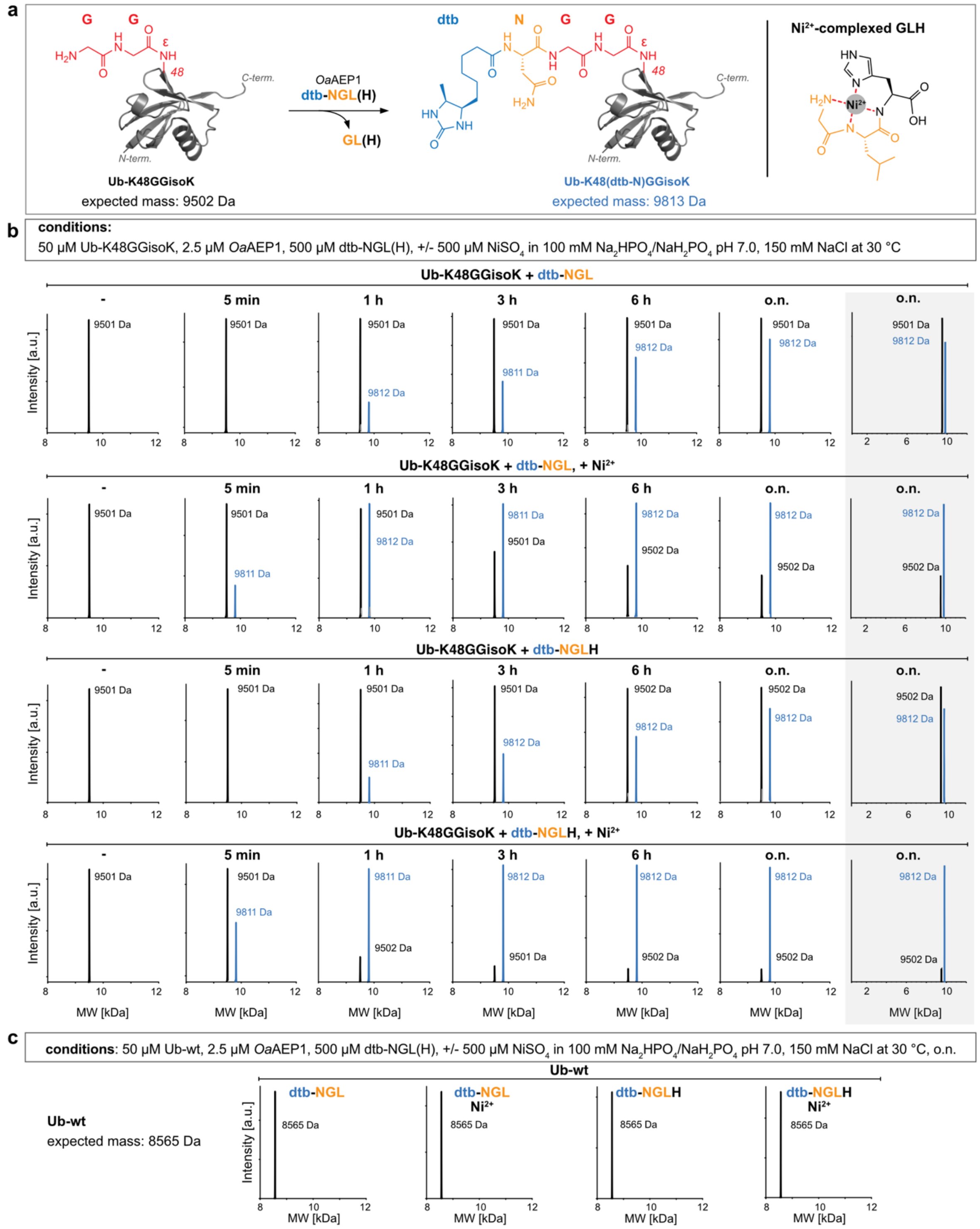

Supplementary Figure S3. Optimization of *Oa*AEP1-mediated protein labeling *via* acceptor nucleophile quenching. **a)** Schematic representation of *Oa*AEP1-mediated internal labeling of Ub-K48GGisoK with desthiobiotin(dtb)-NGL(H) peptide. The side product of the reaction (GLH) that competes with the GGisoK acceptor nucleophile can be complexed with Ni^{2+} (right) and is thereby removed from the reaction equilibrium, pushing it towards the product side. **b)** Time-resolved LC-MS analysis of *Oa*AEP1-mediated labeling of Ub-K48GGisoK under annotated conditions. Labeling with dtb-NGLH in the presence of Ni^{2+} yields the highest labeling efficiency of all tested conditions with approx. 90 % labeled product within 3 h. Full-range LC-MS deconvolutions of overnight timepoints are shown in grey-shaded box. **c)** LC-MS analysis of overnight control reactions with wild type Ub. Independent of conditions, no changes in Ub full-length mass were observed.

Supplementary Figure S4

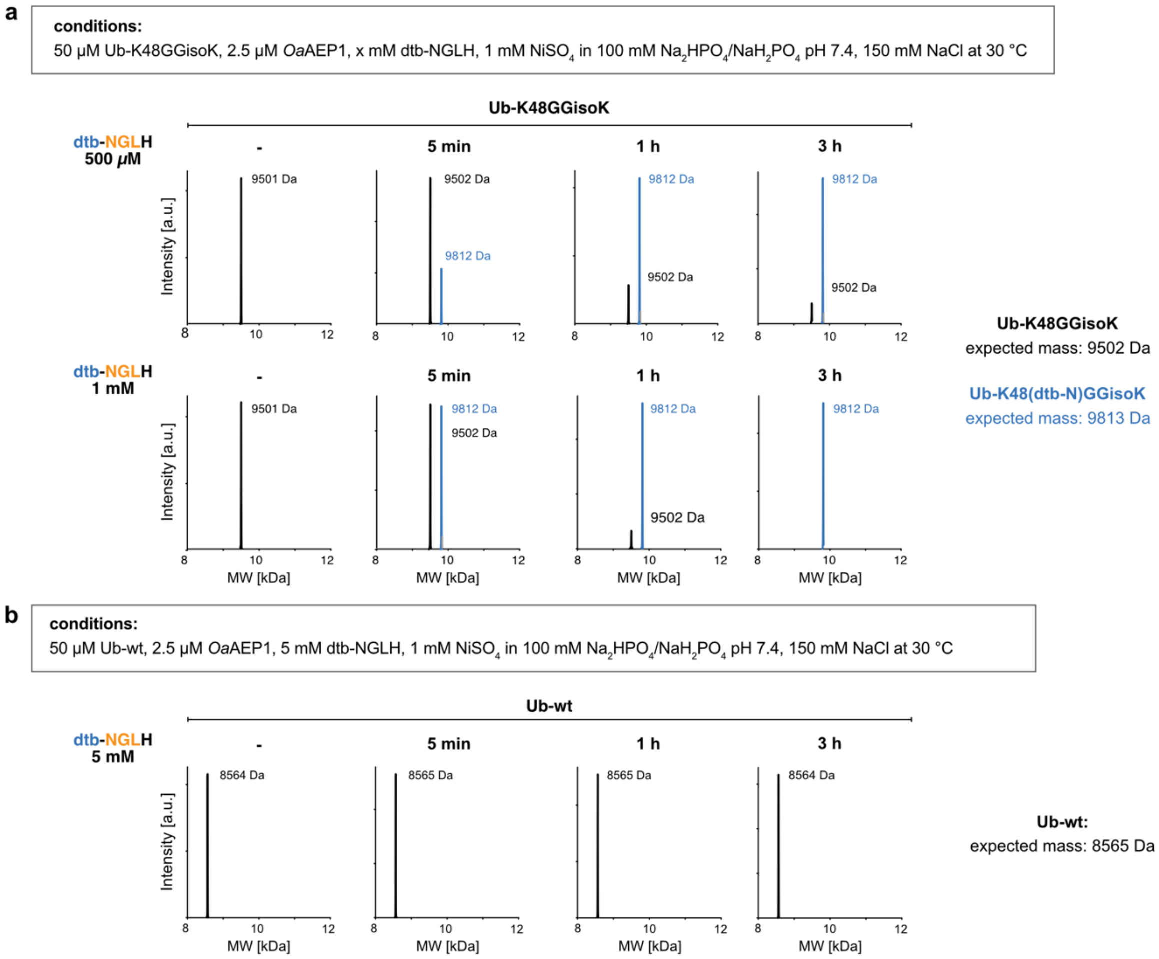

Supplementary Figure S4. Optimizing donor peptide concentrations for efficient labeling. **a)** Time-resolved LC-MS analysis of *Oa*AEP1-mediated labeling of Ub-K48GGisoK with varying dtb-NGLH concentrations. When using 10-fold excess of dtb-NGLH (500 µM) the labeling reaction proceeds to approx. 90 % within 3 h (and approx. 25 % after 5 min). Increasing donor peptide excess to 20-fold (1 mM) enhances labeling efficiency (50 % at 5 min, >90 % after 3 h). **b)** Time-resolved LC-MS analysis of a control reaction with Ub-wt does not show any labeling in absence of acceptor nucleophile.

Supplementary Figure S5

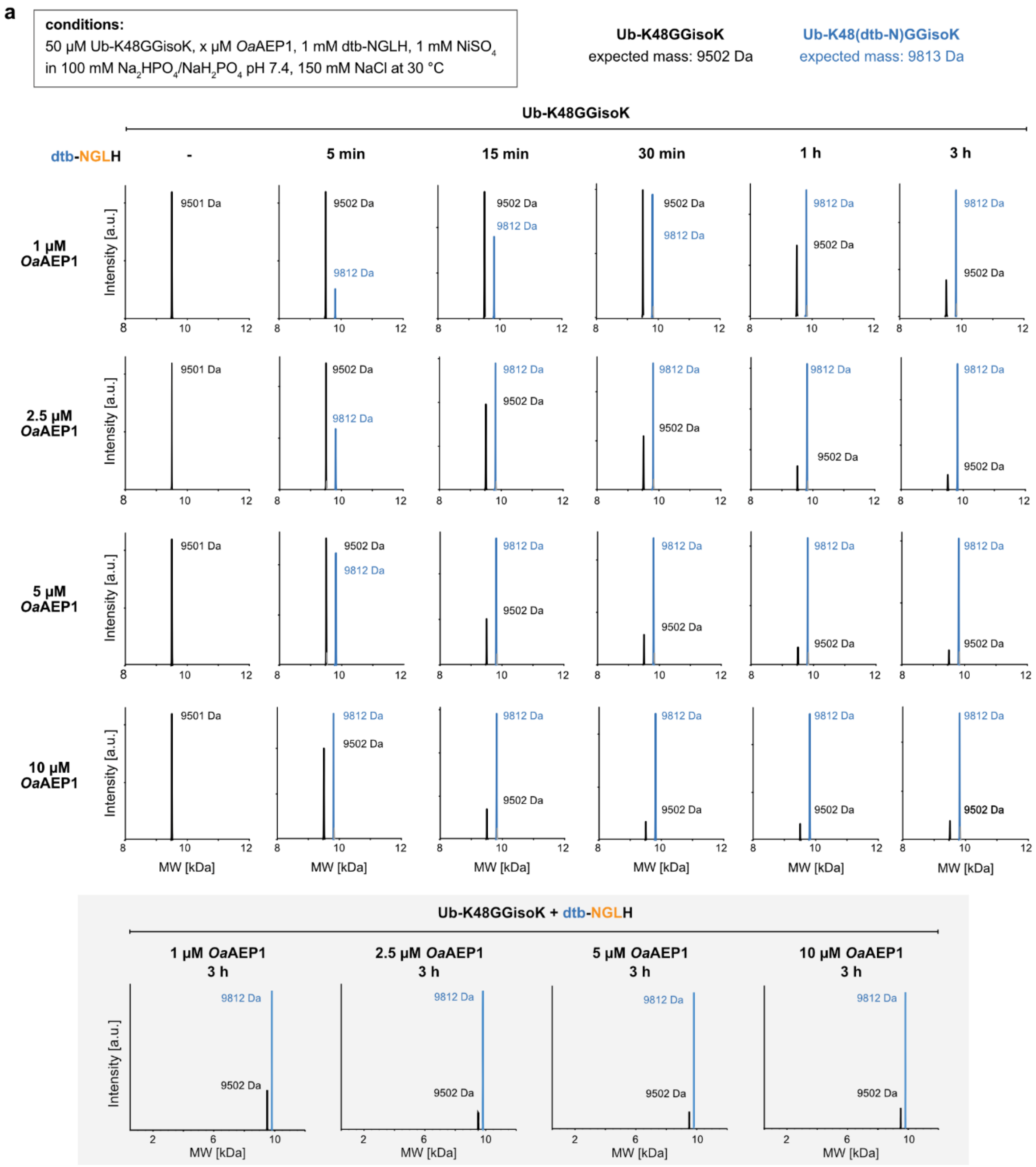

Supplementary Figure S5. Optimizing *Oa*AEP1 concentrations for efficient labeling. Time-resolved LC-MS analysis of *Oa*AEP1-mediated labeling of Ub-K48GGisoK with varying *Oa*AEP1 concentrations. Increasing the concentration of *Oa*AEP1 enhances labeling kinetics of Ub-K48GGisoK with dtb-NGLH. By using 10 µM *Oa*AEP1 (1:5 ratio; *Oa*AEP1:POI) approx. 90 % labeling can be achieved within 30 min. Full-range LC-MS deconvolutions of 3 h timepoints are shown in grey-shaded box.

Supplementary Figure S6

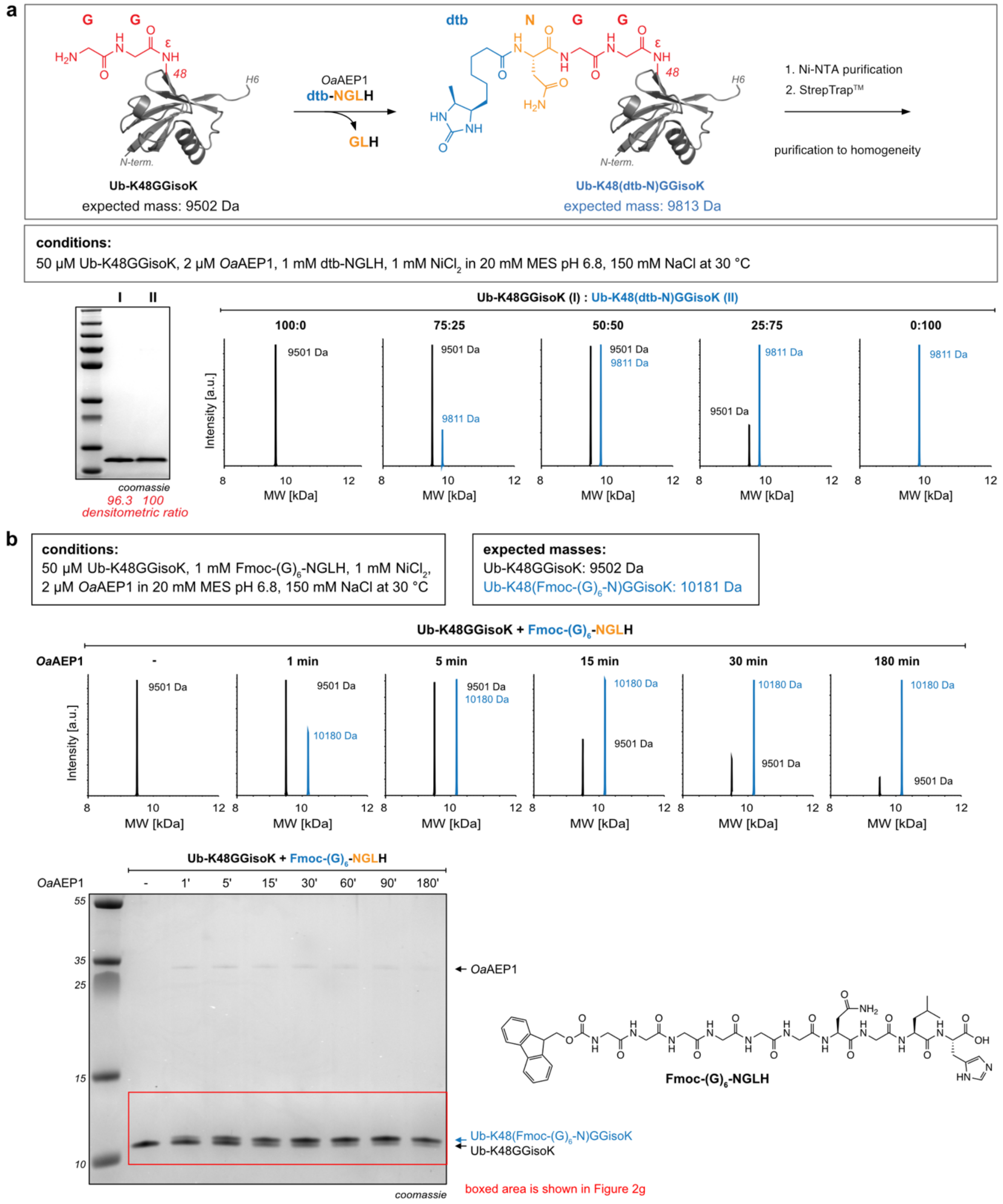

Supplementary Figure S6. Benchmarking of LC-MS as a valid tool for monitoring *Oa*AEP1-mediated protein labeling. **a)** Schematic representation of *Oa*AEP1-mediated internal labeling of Ub-K48GGisoK with dtb-NGLH and further processing by Ni-NTA and Strep-Tag purification (top). SDS-PAGE analysis of pure Ub-K48GGisoK and Ub-K48(dtb-N)GGisoK normalized by Pierce^{TM} BCA protein assay (bottom panel, left). LC-MS analysis of Ub-K48GGisoK and Ub-K48(dtb-N)GGisoK mixtures at the indicated ratios (bottom panel, right). **b)** Time-resolved LC-MS analysis of *Oa*AEP1-mediated labeling of Ub-K48GGisoK with Fmoc-(G)$_6$-NGLH (top) and SDS-PAGE analysis of the very same reaction (bottom left). A gel-shift in agreement with the LC-MS analysis can be observed upon successful labeling with the large peptidic cargo. Chemical structure of Fmoc-(G)$_6$-NGLH (bottom right).

Supplementary Figure S7

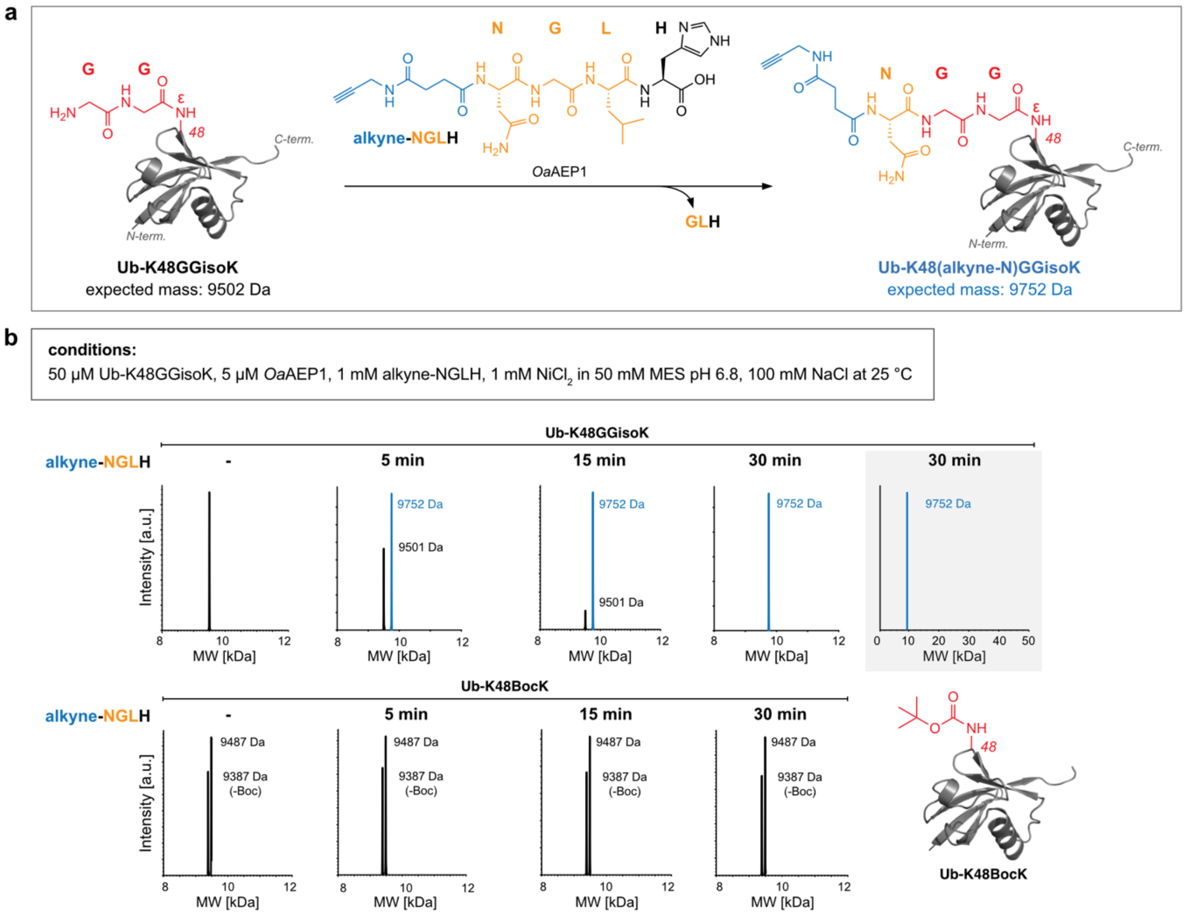

Supplementary Figure S7. *Oa*AEP1-mediated protein labeling with an alkyne-functionalized peptide (alkyne-NGLH). **a)** Schematic representation of *Oa*AEP1-mediated labeling of Ub-K48GGisoK with alkyne-NGLH. **b)** Time-resolved LC-MS analysis of *Oa*AEP1-mediated labeling of Ub-K48GGisoK with alkyne-NGLH under stated conditions shows >90 % conversion within 30 min (top). Full-range LC-MS deconvolution of 30 min timepoint is shown in grey-shaded box. Control reaction with Ub-K48BocK does not show any labeling (bottom). The -100 Da peak corresponds to loss of the tert-butoxycarbonyl in BocK during ionization.

Supplementary Figure S8

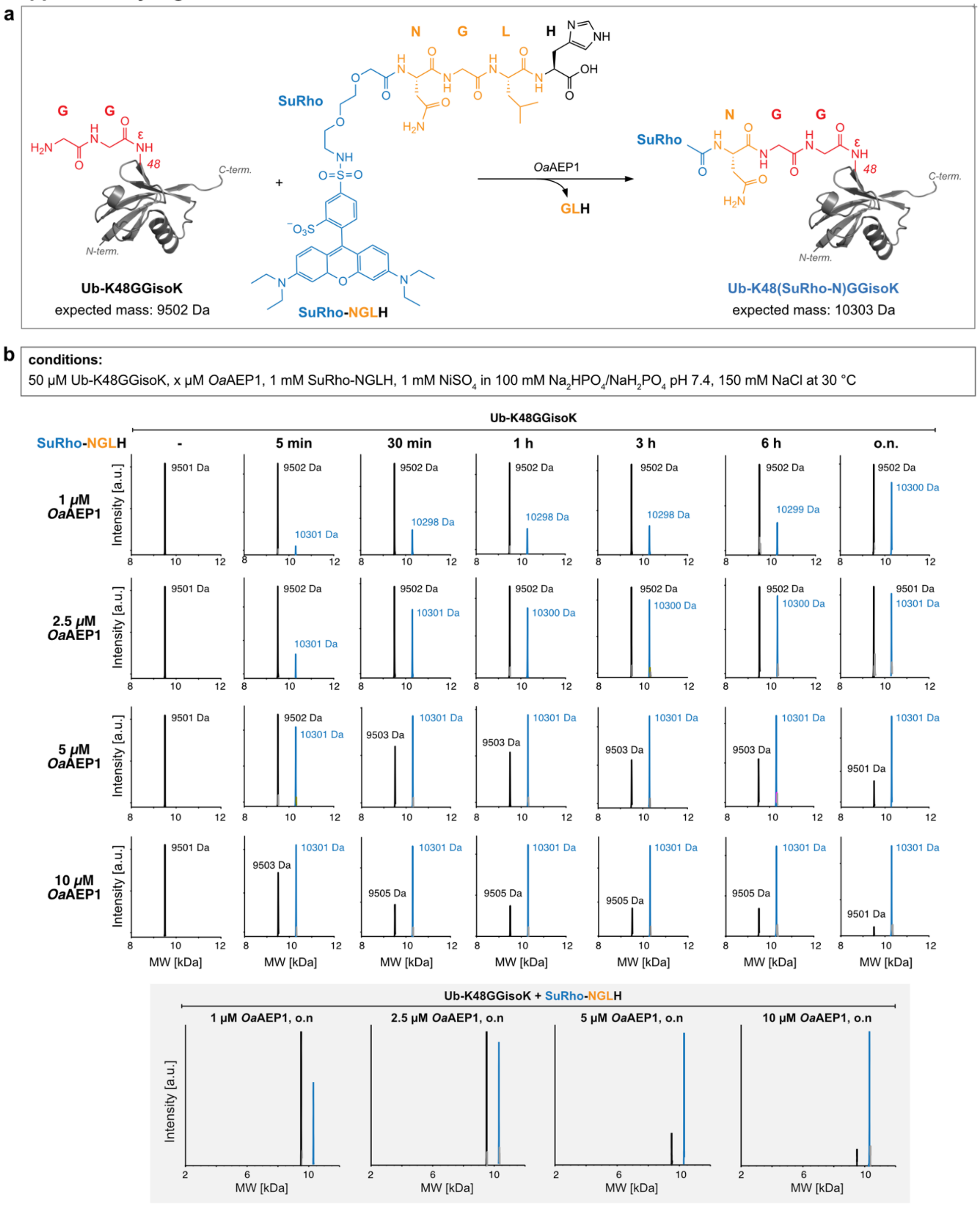

Supplementary Figure S8. *Oa*AEP1-mediated protein labeling with a sulforhodamine-functionalized peptide (SuRho-NGLH). **a)** Schematic representation of *Oa*AEP1-mediated labeling of Ub-K48GGisoK with SuRho-NGLH. **b)** Time-resolved LC-MS analysis of *Oa*AEP1-mediated labeling of Ub-K48GGisoK with SuRho-NGLH. Due to size and hydrophobicity of SuRho-NGLH, increased *Oa*AEP1 concentrations are necessary for efficient labeling. Incubation with 10 µM *Oa*AEP1 (1:5 ratio; *Oa*AEP1:POI) yields approx. 90 % labeling overnight. Full-range LC-MS deconvolutions of overnight timepoints are shown in grey-shaded box.

Supplementary Figure S9

a

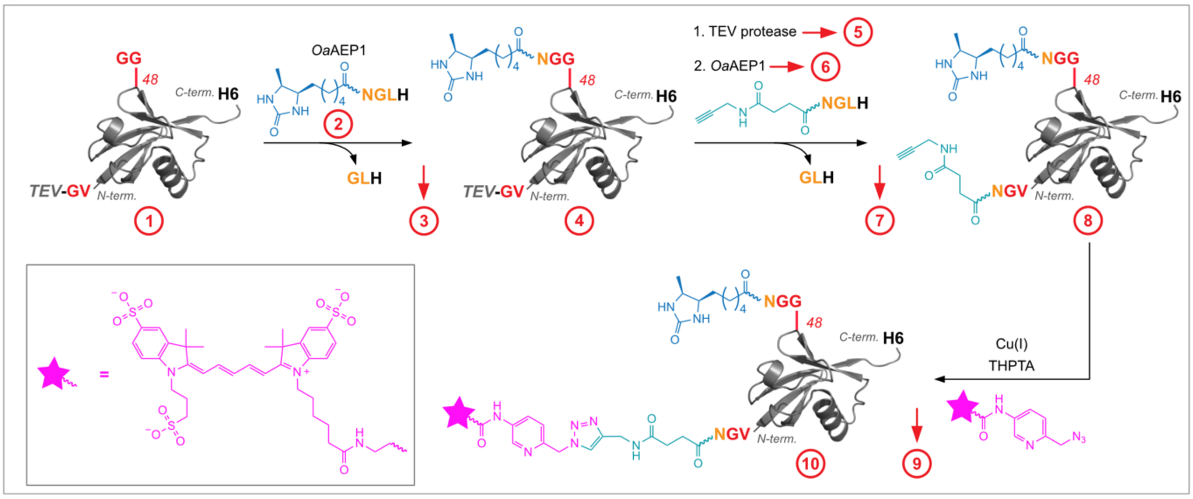

b

procedure and conditions:
50 µM TEV-GV-Ub-K48GGisoK (1), 1 mM dtb-NGLH, 500 µM $NiCl_2$ and 2 µM *Oa*AEP1 in 20 mM MES, 150 mM NaCl, pH 6.8 at 30 °C for 100 min (2). NiNTA purification (flowthrough: 3; eluate: 4). TEV cleavage with 0.2 U/µl TEV protease for 1 hour at 30 °C (5). Addition of 1 mM alkyne-NGLH, 500 µM $NiCl_2$ and 2 µM *Oa*AEP1 in 20 mM MES, 150 mM NaCl, pH 6.8; labeling at 30 °C for 150 min (6). NiNTA purification (flowthrough: 7; eluate: 8). CuAAC on NiNTA beads with 50 µM $CuSO_4$, 250 µM THPTA, 2.5 mM ascorbic acid and 100 µM Sulfo-Cy5-picolyl azide for 60 min at RT. NiNTA purification (flowthrough: 9; eluate: 10).

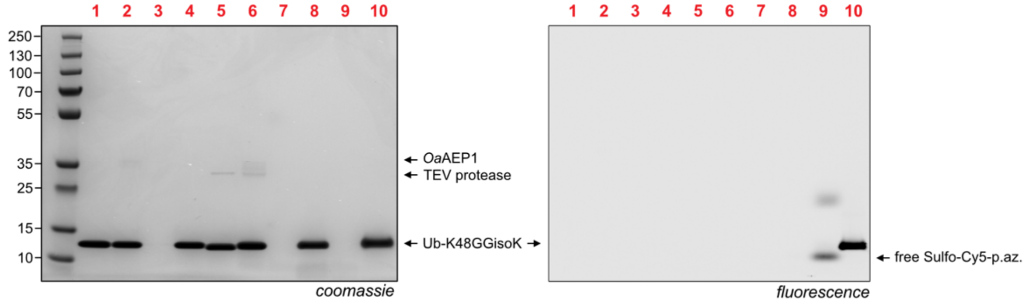

c

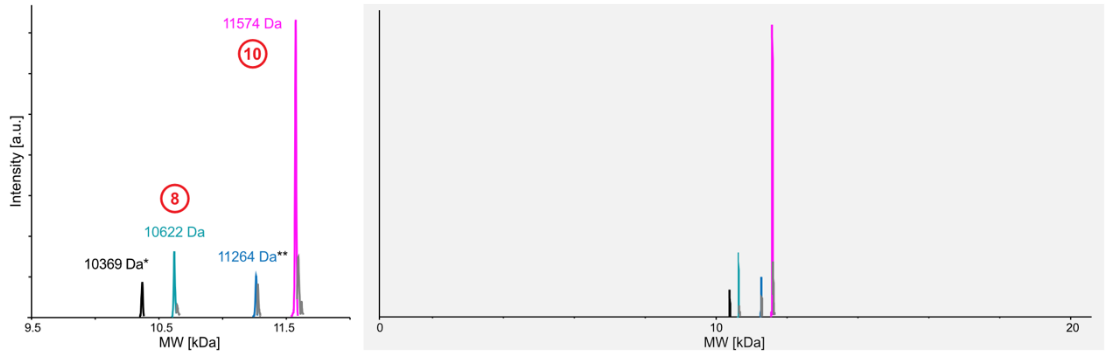

(1) starting material: *TEV*-**GV-Ub-K48GGisoK**
expected mass: 10952 Da, observed: 10952 Da, see Figure 3b

(4) ligation of dtb-NGLH onto GGisoK: *TEV*-**GV-Ub-K48(dtb-N)GGisoK**
expected mass: 11262 Da, observed: 11262 Da, see Figure 3b

(8) transpeptidation of alkyne-NGLH onto N-terminal GV: **alkyne-NGV-Ub-K48(dtb-N)GGisoK**
expected mass: 10621 Da, observed: 10621 Da or 10622 Da, also see Figure 3b

(10) CuAAC click product: **Sulfo-Cy5-triazole-NGV-Ub-K48(dtb-N)GGisoK**
expected mass: 11574 Da, observed: 11574 Da

*observed mass 10369 Da corresponds to **GV-Ub-K48(dtb-N)GGisoK** (expected: 10370 Da)
i.e. starting material from second transpeptidation reaction after TEV-cleavage
observed mass 11264 Da corresponds to **Sulfo-Cy5-triazole-NGV-Ub-K48GGisoK (expected: 11264 Da)
i.e. starting material that got transpeptidated with alkyne-NGLH and subsequently underwent CuAAC

Supplementary Figure S9. *Oa*AEP1-mediated dual labeling of TEV-GV-Ub-K48GGisoK. **a)** Schematic representation of *Oa*AEP1-mediated dual labeling of **1**. In a first step the GGisoK moiety is labeled with dtb-NGLH (**4**) followed by TEV cleavage (**5**) to reveal an N-terminal GV moiety. In a second step the unmasked N-

terminal GV moiety of **5** is modified with alkyne-NGLH (**6**) to yield **8**. Copper(I)-catalyzed azide-alkyne cycloaddition (CuAAC) with Sulfo-Cy5-picolyl azide yields fluorescently labeled product **10**. **b)** SDS-PAGE analysis of the multistep procedure for dual labeling. Sample identity is indicated with red numbers that correspond to Supplementary Figure S9a and the described procedure. Coomassie staining confirms integrity of Ub construct over the course of dual labeling (left). In-gel fluorescence imaging shows successful fluorescent labeling with Sulfo-Cy5-picolyl azide (right). **c)** LC-MS analysis of successful CuAAC of Sulfo-Cy5-picolyl azide to **8** yields **10** (top). Full-range LC-MS deconvolution is shown in grey-shaded box. Relevant expected and observed masses of the individual labeling steps are depicted (bottom).

Supplementary Figure S10

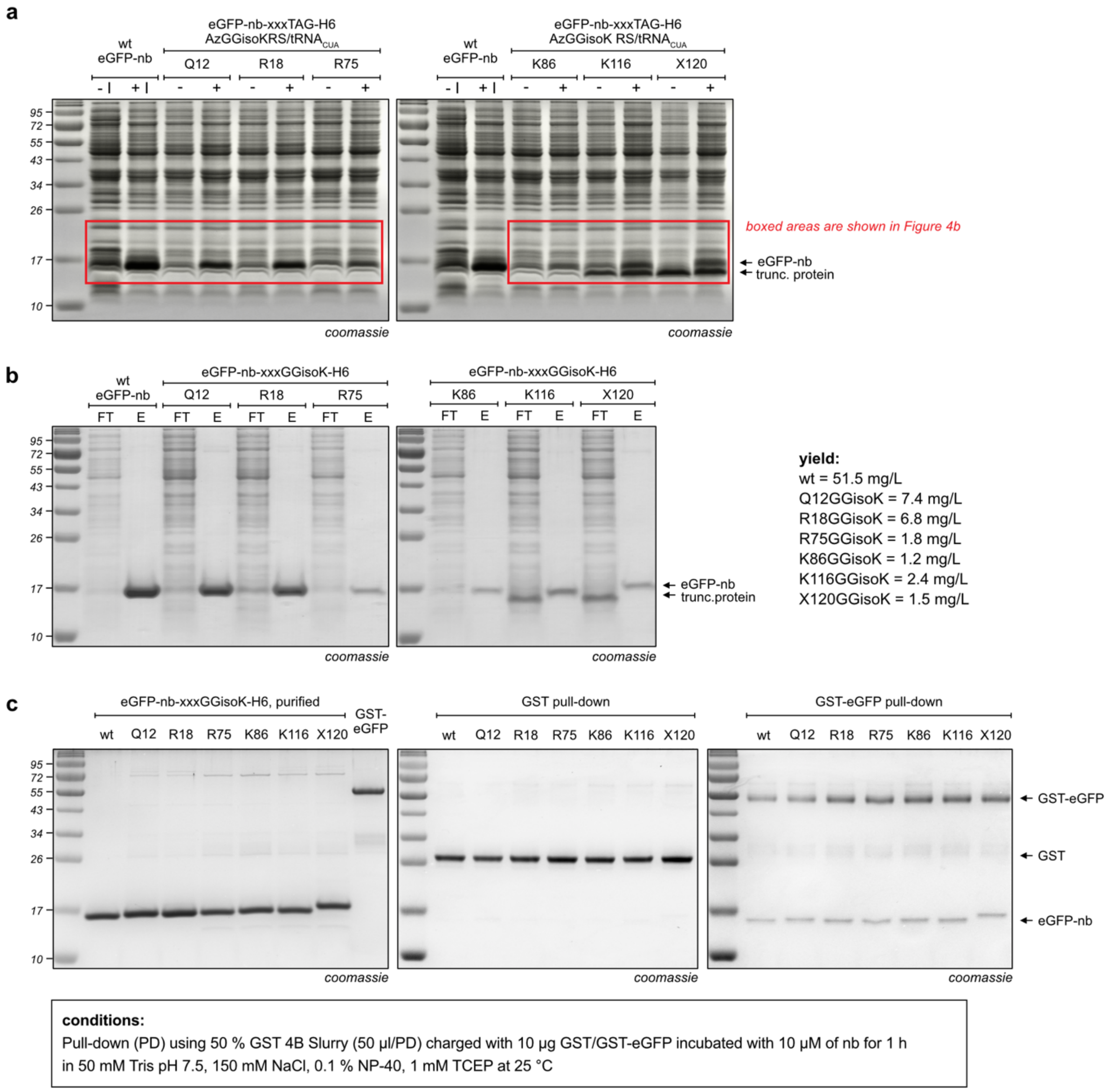

Supplementary Figure S10. Expression, purification and characterization of GGisoK-bearing eGFP-nanobodies (eGFP-nbs). **a)** SDS-PAGE showing expression of site-specifically modified eGFP-nb with AzGGisoK at different positions (-/+ I = with and without induction; -/+ = with and without AzGGisoK). **b)** NiNTA purification of wild type and AzGGisoK-bearing eGFP-nbs (FT = flow-through; E = elution). Purification yields of expressed nanobody variants are listed. **c)** Pull-down (PD) experiments of eGFP-nbs with GST/GST-eGFP. SDS-PAGE of purified proteins used for the PD experiments (left). SDS-PAGE of PD’s with immobilized GST (middle). SDS-PAGE of PD’s with immobilized GST-eGFP confirming the ability of GGisoK-bearing eGFP-nbs to bind to eGFP (right).

Supplementary Figure S11

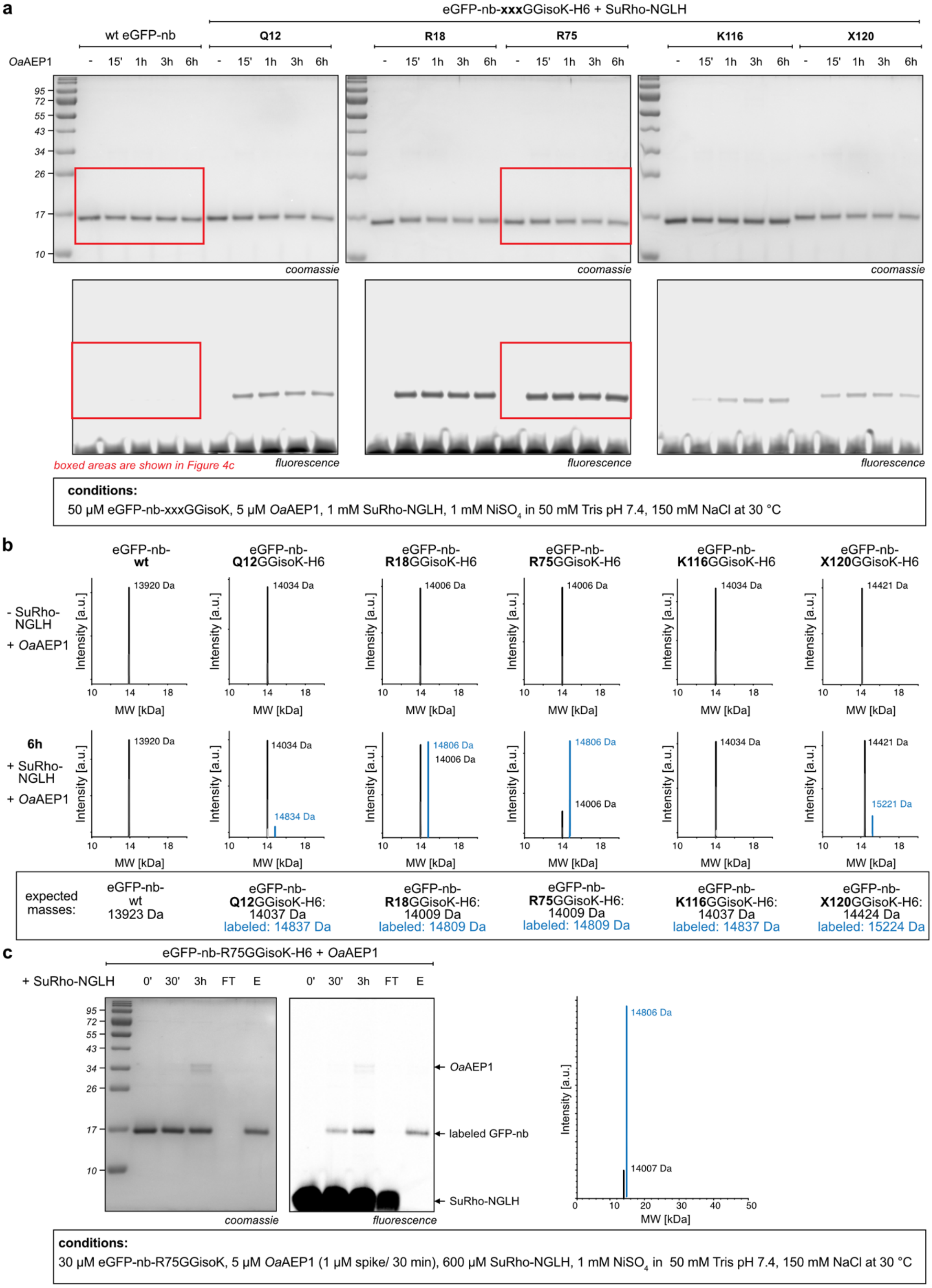

Supplementary Figure S11. *Oa*AEP1-mediated labeling of GGisoK-bearing eGFP-nbs with SuRho-NGLH. **a)** SDS-PAGE analysis of *Oa*AEP1-mediated labeling of eGFP-nb-**xxx**GGisoK. Coomassie staining confirms pure, equally concentrated eGFP-nbs (top). In-gel fluorescence shows specific labeling of GGisoK-bearing nanobodies with varying efficiencies depending on the accessibility of the GGisoK moiety (bottom). **b)** LC-MS analysis of SuRho-NGLH labeling reactions of the different GGisoK-bearing eGFP-nbs. eGFP-nb-R75GGisoK shows the best labeling efficiency (approx. 75 % after 6 h). **c)** Preparative *Oa*AEP1-mediated labeling of eGFP-nb-

R75GGisoK with SuRho-NGLH. Coomassie stain depicts the labeling and purification process (left). In-gel fluorescence confirms labeling of eGFP-nb-R75GGisoK and removal of residual *Oa*AEP1 and free SuRho-NGLH (middle). LC-MS analysis shows the purified, fluorescently labeled eGFP-nb-R75(SuRho-N)GGisoK (approx. 90 %, right).

Supplementary Figure S12

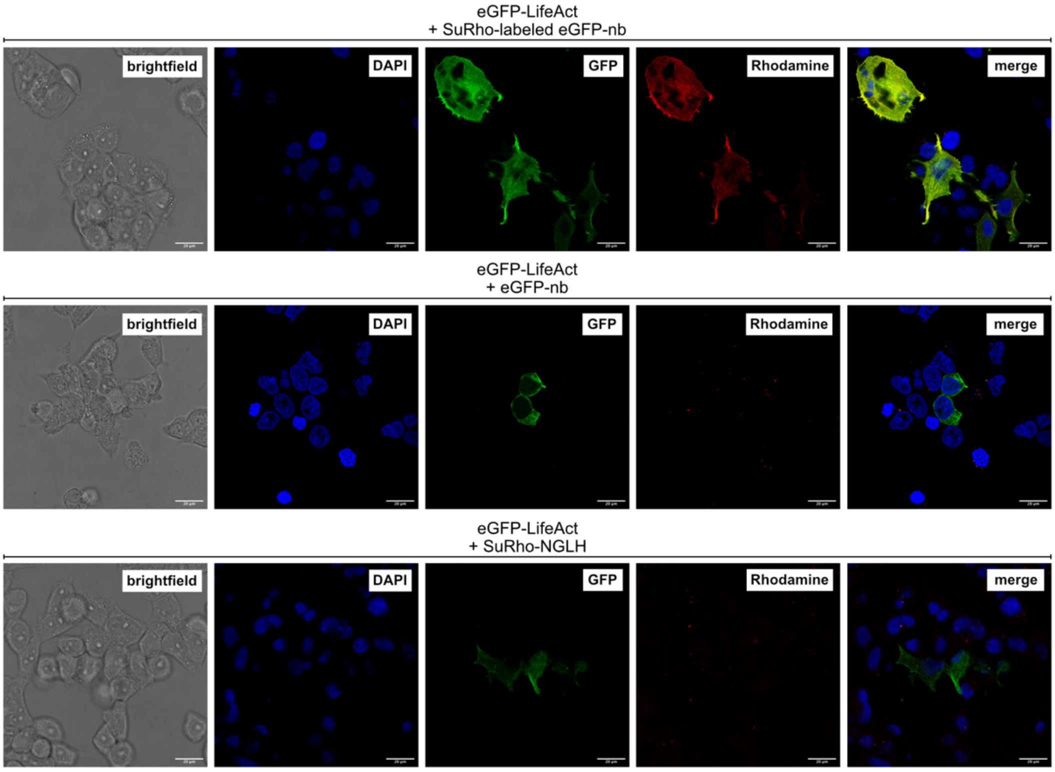

Supplementary Figure S12. Confocal microscopy images of fixed HEK293T cells overexpressing eGFP-LifeAct. Shown are brightfield (grey), DAPI (blue), GFP (green), SuRho (red) and merge (DAPI, GFP and SuRho) images of transfected cells treated with 300 nM SuRho-labeled eGFP-nb [top, eGFP-nb-R75(SuRho-N)GGisoK], unlabeled eGFP-nb (middle, eGFP-nb-R75GGisoK)) or SuRho-NGLH peptide (bottom). Scale bars correspond to 20 µm.

Supplementary Figure S13

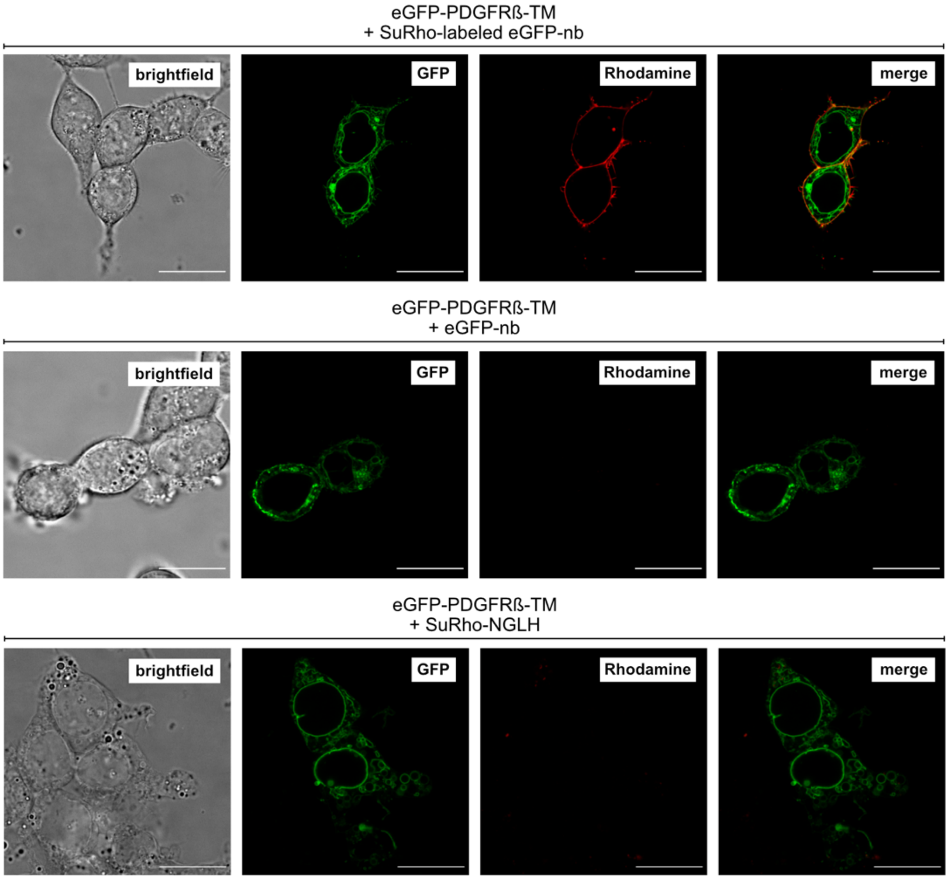

Supplementary Figure S13. Live-cell microscopy of HEK293T cells overexpressing eGFP-PDGFR-TM. Shown are brightfield (grey), GFP (green), SuRho (red) and merge (GFP and SuRho) images of transfected cells treated with 300 nM SuRho-labeled eGFP-nb [top, eGFP-nb-R75(SuRho-N)GGisoK)], unlabeled eGFP-nb (middle, eGFP-nb-R75GGisoK)) or SuRho-NGLH peptide (bottom). Scale bars correspond to 20 µm.

Supplementary Figure S14

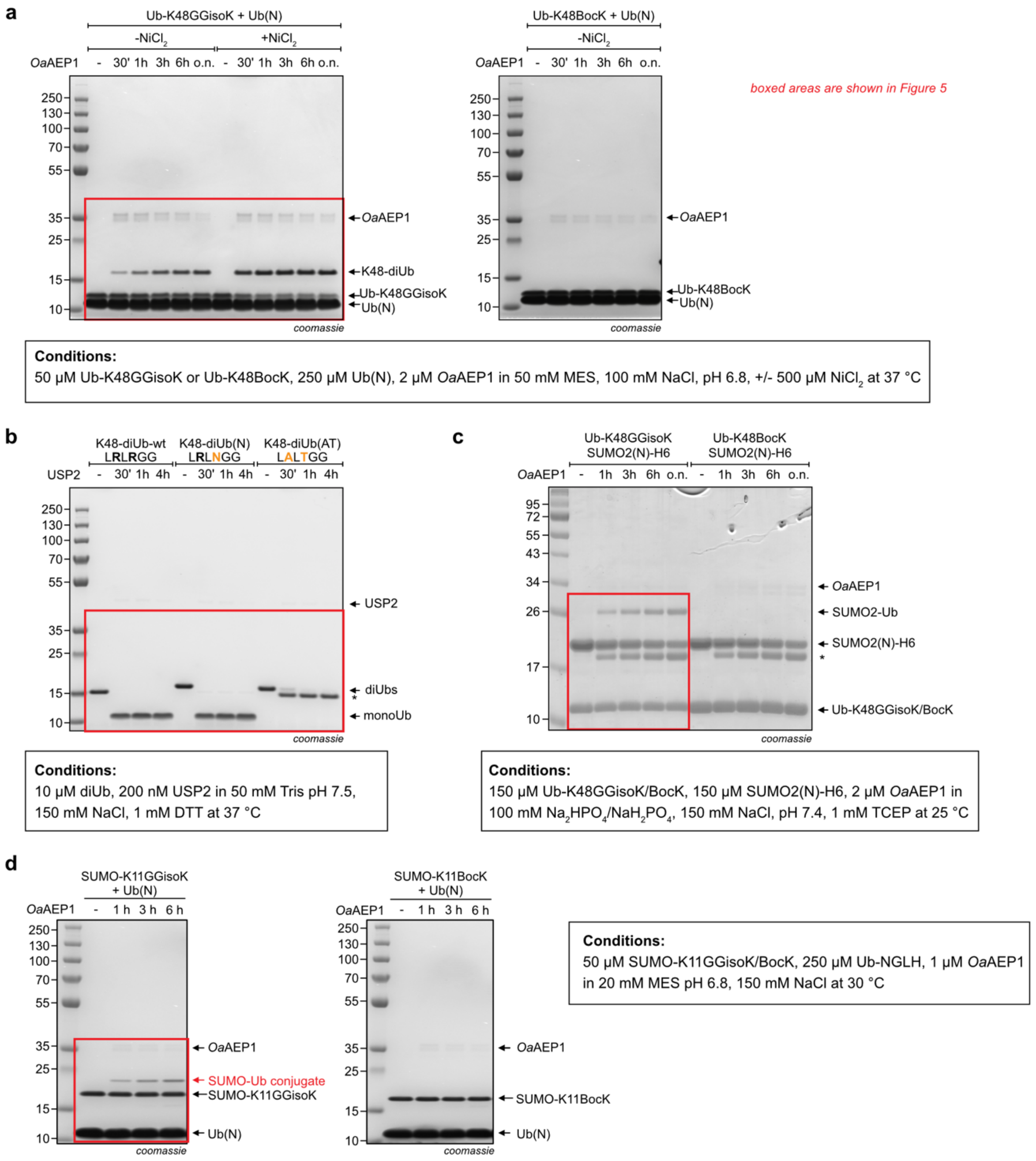

Supplementary Figure S14. *Oa*AEP1-mediated ubiquitylation and SUMOylation. **a)** SDS-PAGE analysis of *Oa*AEP1-mediated ubiquitylation of Ub-K48GGisoK using Ub(N). K48-diUb formation is more efficient in presence of $NiCl_2$ (left). When using Ub-K48BocK instead of Ub-K48GGisoK no K48-diUb formation is observed (right). **b)** Incubation of differently linked K48-diUbs [K48-diUb-wt, K48-diUb(N), K48-diUb(AT)] with deubiquitylase USP2 shows that K48-diUb wt and K48-diUb(N) are quantitatively cleaved within 30 min, while K48-diUb(AT) is recalcitrant towards USP2-mediated hydrolysis [*denotes cleavage of the C-terminal H6-tag of the acceptor Ub of K48-diUb(AT)]. **c)** SDS-PAGE analysis of *Oa*AEP1-mediated SUMOylation of Ub-K48GGisoK using SUMO2(N)-H6 [*denotes hydrolysis of the C-terminal *Oa*AEP1 motif leading to cleavage of the H6-tag of SUMO2(N)-H6]. **d)** SDS-PAGE analysis of *Oa*AEP1-mediated ubiquitylation of SUMO-K11GGisoK using Ub(N) (left). When using SUMO-K11BocK instead of SUMO-K11GGisoK no SUMO-Ub conjugate is observed (right). Ubiquitylation yields were determined via densitometry using ImageJ (S14 a: 60.5 %; S14 c: 35.0 %; S14 d: 28.2 %).

Supplementary Figure S15

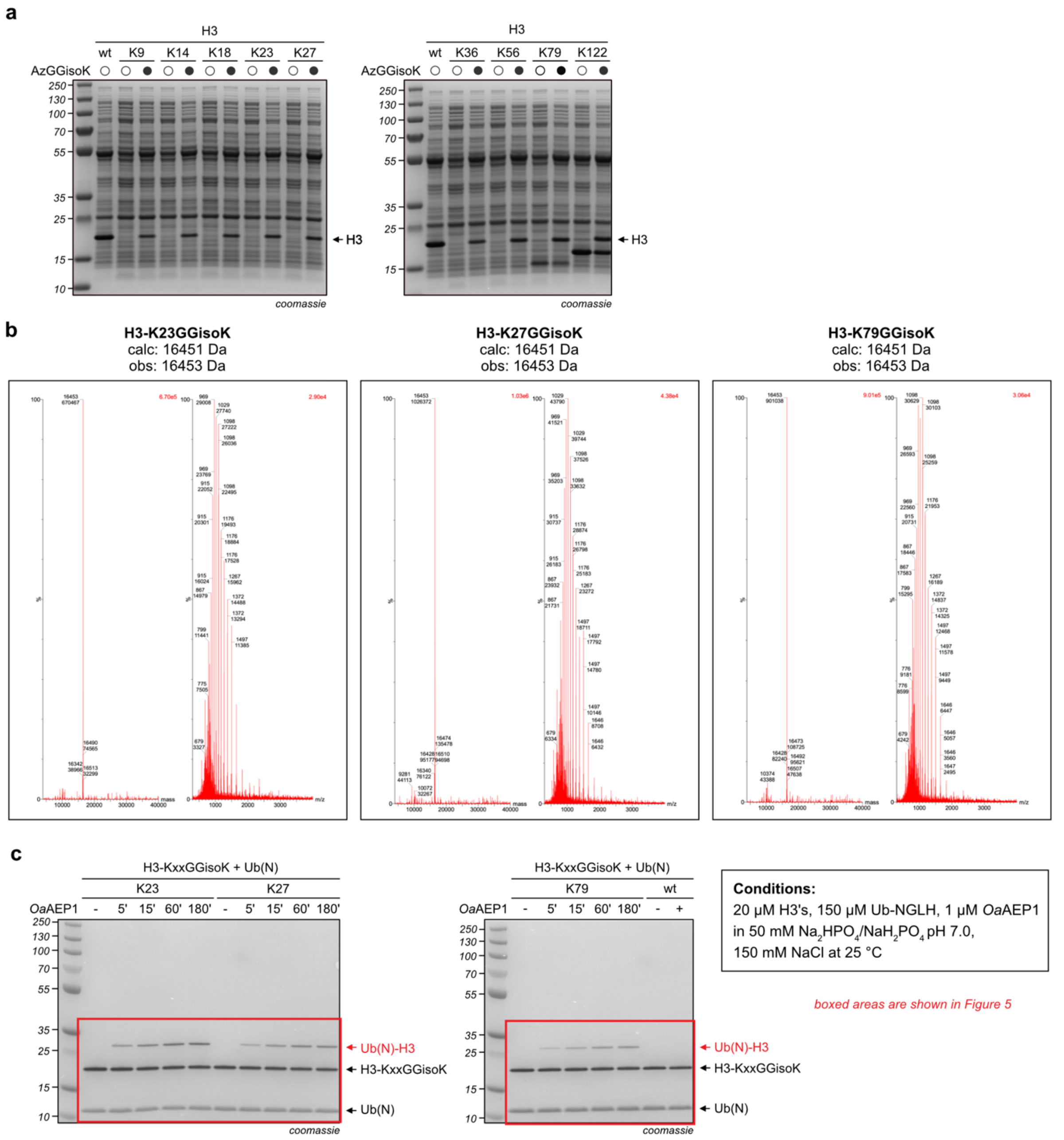

Supplementary Figure S15. *Oa*AEP1-mediated ubiquitylation of Histone H3. **a)** SDS-PAGE showing expression of site-specifically modified H3 with AzGGisoK at different positions. **b)** HPLC-MS analysis of indicated GGisoK-bearing H3 variants. **c)** SDS-PAGE analysis of *Oa*AEP1-mediated ubiquitylation of H3-K23/27/79-GGisoK-H_6 using Ub(N) (reactions conditions denoted in box). When using H3 wt instead of H3-KxxGGisoK-H_6 no Ub-H3 conjugate is observed. Ubiquitylation yields were determined via densitometry using ImageJ (H3-K23GGisoK: 31.2 %; H3-K27GGisoK: 35.1 %; H3-K79GGisoK: 35.3 %).

Supplementary Tables

Supplementary Table S1. Primers for introduction of TAG mutants

Construct	Primer	Sequence (5‘ => 3‘)
pPylT_pelB-eGFP-nanobody-Q12TAG-H_6	Short Fw	CTCTCCTGTGCAGCCTCTGG
	Short Rv	CACCAAGGCTCCCCCAGAC
	Tail Fw	TAGCCGGGGGGGTCTCTGAGACTCTCCTGTGCAGCCTCTGG
	Tail Rv	TCTCAGAGACCCCCCCGGCTACACCAAGGCTCCCCCAGAC
pPylT_pelB-eGFP-nanobody-R18TAG-H_6	Short Fw	CTCTCCTGTGCAGCCTCTGG
	Short Rv	CACCAAGGCTCCCCCAGAC
	Tail Fw	CAGCCGGGGGGGTCTCTGTAGCTCTCCTGTGCAGCCTCTGG
	Tail Rv	CTACAGAGACCCCCCCGGCTGCACCAAGGCTCCCCCAGAC
pPylT_pelB-eGFP-nanobody-R75TAG-H_6	Short Fw	TATCTGCAAATGAACAGCCTGAAACCTG
	Short Rv	GGCGTCGTCTCTGGAGATGG
	Tail Fw	TAGAATACGGTGTATCTGCAAATGAACAGCCTGAAACCTG
	Tail Rv	CACCGTATTCTAGGCGTCGTCTCTGGAGATGG
pPylT_pelB-eGFP-nanobody-R86TAG-H_6	Short Fw	ACGGCCGTGTATTACTGTAATGTCA
	Short Rv	CAGGCTGTTCATTTGCAGATACACC
	Tail Fw	TAGCCTGAGGACACGGCCGTGTATTACTGTAATGTCA
	Tail Rv	GTCCTCAGGCTACAGGCTGTTCATTTGCAGATACACC
pPylT_pelB-eGFP-nanobody-K116TAG-H_6	Short Fw	CACCACCATCACCATCACTAAAGCT
	Short Rv	GGAGACGGTGACCTGGGTC
	Tail Fw	TCAAAGTAGAAGCACCACCATCACCATCACTAAAGCT
	Tail Rv	CTTCTACTTTGAGGAGACGGTGACCTGGGTC
pPylT_pelB-eGFP-nanobody-X120TAG-H_6	Short Fw	CACCACCATCACCATCACTAAAGCTC
	Short Rv	CTTTTTCTTTGAGGAGACGGTGACCT
	Tail Fw	GGCGGCTAGGGCAGCCACCACCATCACCATCACTAAAGCTC
	Tail Rv	GCTGCCCTAGCCGCCCTTTTTCTTTGAGGAGACGGTGACCT
pPylT_SUMO2-K11TAG-H_6	Short Fw	ACCGAAAACAACGATCATATTAACCTGAAAGT
	Short Rv	TTCATCCGCCATGGTTAATTCCTCCT
	Tail Fw	AAACCGAAAGAAGGCGTGTAGACCGAAAACAACGATCATATTAA CCTGAAAGT
	Tail Rv	CTACACGCCTTCTTTCGGTTTTTCATCCGCCATGGTTAATTCCTCCT

**pBAD_H3-K23TAG-H_6**	Short Fw	TCCGCTCCTGCTACCGG
	Short Rv	GGTGGCCAGCTGCTTG
	Tail Fw	TAGGCAGCCAGGAAGTCCGCTCCTGCTACCGG
	Tail Rv	CTTCCTGGCTGCCTAGGTGGCCAGCTGCTTG
**pBAD_H3-K27TAG-H_6**	Short Fw	TCCGCTCCTGCTACCGG
	Short Rv	GGTGGCCAGCTGCTTG
	Tail Fw	AAGGCAGCCAGGTAGTCCGCTCCTGCTACCGG
	Tail Rv	CTACCTGGCTGCCTTGGTGGCCAGCTGCTTG
**pBAD_H3-K79TAG-H_6**	Short Fw	ACCGACCTGCGCTTCC
	Short Rv	AGCGATCTCCCGGACCAG
	Tail Fw	CAGGACTTCTAGACCGACCTGCGCTTCC
	Tail Rv	CTAGAAGTCCTGAGCGATCTCCCGGACCAG

Supplementary Table S2. Primers for introduction of *Oa*AEP1 motifs

Construct	Primer	Sequence (5‘ => 3‘)
**pET17b_Ub(NGL)**	Short Fw	TGATAAGAATTCTGAAGATATCCATC
	Short Rv	TCTGAGGACCAGGTGCAG
	Tail Fw	CTCAACGGTCTCTGATAAGAATTCTGCAGATATCCATC
	Tail Rv	GAGACCGTTGAGTCTGAGGACCAGGTGCAG
**pET17b_Ub(NGLH)**	Short Fw	TGATAAGAATTCTGCAGATATCCATCACACTGG
	Short Rv	TCTGAGGACCAGGTGCAGGG
	Tail Fw	CTCAATGGTCTCCACTGATAAGAATTCTGCAGATATCCATCACA CTGG
	Tail Rv	GTGGAGACCATTGAGTCTGAGGACCAGGTGCAGGG
**pET17b_SUMO2(NGLH$_6$)**	Short Fw	CACCACCACCACCATCACC
	Short Rv	AAACACATCAATGGTATCTTCA
	Tail Fw	CAGCAGCAGGAACGGCCTGCACCACCACCATGCCC
	Tail Rv	CAGGCCGTTCTGCTGCTGAAACACATCAATGGTATCTTCA

Supplementary Table S3. Plasmids

Plasmid	Description
**pET29b_H_6-Ub-*Oa*AEP1b-C274A**	H_6 Ub *Oa*AEP1b C274A under an IPTG inducible T7 promoter with a C-terminal H_6-tag.
**pBK_*M.b.*-AzGGKRS**	*M. barkeri* AzGGK aminoacyl-tRNA-synthetase (aaRS) under a constitutive GlnS promoter.
**pBK_*M.a.*-AzGGKRS**	*M. alvus* AzGGK aminoacyl-tRNA-synthetase (aaRS) under a constitutive GlnS promoter.
**pBK_*M.b.*-wt-RS**	*M. bakeri* wt aaRS under a constitutive GlnS promoter.
**pBK_*M.a.*-wt-RS**	*M. alvus* wt aaRS under a constitutive GlnS promoter.
**pEVOL_*M.b.*-AzGGKRS**	*M. barkeri* AzGGK aminoacyl-tRNA-synthetase (aaRS) under a constitutive GlnS and an arabinose inducible promoter with a PylT copy und an constitutive promotor.
**pPylT_sfGFP-N150TAG-H_6**	sfGFP-N150TAG-H_6 under an arabinose promoter with a C-terminal H_6-tag and a *M.b.* PylT copy under a constitutive promoter.
**pPylT_UbK48TAG-H_6**	UbK48TAG-H_6 under an arabinose promoter with a C-terminal H_6-tag and a *M.a.* PylT copy under a constitutive promoter.
**pPylT_SUMO2-K11TAG-H_6**	SUMO-K11TAG-H_6 under an arabinose promoter with a C-terminal H_6-tag and a *M.b.* PylT copy under a constitutive promoter.
**pBAD_H3-K23TAG-H_6**	H3-K23TAG-H_6 under an arabinose promotor with a C-terminal H_6-tag.
**pBAD_H3-K27TAG-H_6**	H3-K27TAG-H_6 under an arabinose promotor with a C-terminal H_6-tag.
**pBAD_H3-K79TAG-H_6**	H3-K79TAG-H_6 under an arabinose promotor with a C-terminal H_6-tag.
**pET17b_Ub-wt**	Ubiquitin wt under an IPTG-inducible T7 promoter.
**pET17b_Ub(NGL)**	Ubiquitin with C-terminus LRLNGG under an IPTG- inducible T7 promoter.
**pET17b_Ub(AT)**	Ubiquitin with C-terminus LALTGG under an IPTG-inducible T7 promoter.

**pET17b_Ub(NGLH)**	Ubiquitin with C-terminus LRLNGLH under an IPTG-inducible T7 promoter.
**pET17b_SUMO2(NGLH$_6$)**	SUMO2 with C-terminus QQQNGLH$_6$ under an IPTG-inducible T7 promoter.
**pPylT_TEV-GV-UbK48TAG-H$_6$**	UbK48TAG-H$_6$ under an arabinose promoter with a C-terminal H$_6$-tag, an N-terminal TEV site followed by a "GV" motif and a *M.a.* PylT copy under a constitutive promoter.
**pPylT_pelB-eGFP-nanobodyXXX TAG-H$_6$**	pelB-eGFP-nanobody with a TAG codon at denoted position under an arabinose promoter with a C-terminal H$_6$-tag and a *M.b.* PylT copy under a constitutive promoter.
**pET17_GST**	GST under an IPTG-inducible T7 promoter.
**pET17_GST_eGFP**	GST-eGFP under an IPTG-inducible T7 promoter.
**pGEX_6P_1_UBE2R1**	GST-UBE2R1 (Cdc34) under an IPTG-inducible T7 promoter.
**pET29b_Srt2A-TEV-H$_6$**	Srt2A with a C-terminal TEV-site followed by a H$_6$-tag under an IPTG inducible T7 promoter with a C-terminal H$_6$-tag.
**pET28a_H$_6$-Thr-USP2**	Catalytic domain of USP2 with an N-terminal H$_6$-tag followed by a thrombin cleavage site under an IPTG-inducible T7 promoter.
**mEGFP-Lifeact-7**	Lifeact-mEGFP under a CMV promoter (Addgene Nr. 54610).
**pDisplay-mSA-EGFP-TM**	mSA-EGFP-TM under a CMV promoter (Addgene Nr. 39863).

Supplementary Notes

Supplementary Note 1: Amino acid sequences of proteins

Bacterial constructs

H_6-Ubiquitin-*Oa*AEP1b (C247A)
MHHHHHHMQIFVKTLTGKTITLEVEPSDTIENVKAKIQDKEGIPPDQQRLIFAGKQLEDGRTLSDYNIQK
ESTLHLVLRLRGGARDGDYLHLPSEVSRFFRPQETNDDHGEDSVGTRWAVLIAGSKGYANYRHQAGV
CHAYQILKRGGLKDENIVVFMYDDIAYNESNPRPGVIINSPHGSDVYAGVPKDYTGEEVNAKNFLAAIL
GNKSAITGGSGKVVDSGPNDHIFIYYTDHGAAGVIGMPSKPYLYADELNDALKKKHASGTYKSLVFYL
EACESGSMFEGILPEDLNIYALTSTNTTESSWAYYCPAQENPPPPEYNVCLGDLFSVAWLEDSDVQNSW
YETLNQQYHHVDKRISHASHATQYGNLKLGEEGLFVYMGSNPANDNYTSLDGNALTPSSIVVNQRDA
DLLHLWEKFRKAPEGSARKEEAQTQIFKAMSHRVHIDSSIKLIGKLLFGIEKCTEILNAVRPAGQPLVDD
WACLRSLVGTFETHCGSLSEYGMRHTRTIANICNAGISEEQMAEAASQACASIP*

***M. barkeri* AzGGKRS (mutations: L274A, N311Q and C313S)**
MDKKPLDVLISATGLWMSRTGTLHKIKHHEVSRSKIYIEMACGDHLVVNNSRSCRTARAFRHHKYRKT
CKRCRVSDEDINNFLTRSTESKNSVKVRVVSAPKVKKAMPKSVSRAPKPLENSVSAKASTNTSRSVPSP
AKSTPNSSVPASAPAPSLTRSQLDRVEALLSPEDKISLNMAKPFRELEPELVTRRKNDFQRLYTNDREDY
LGKLERDITKFFVDRGFLEIKSPILIPAEYVERMGINNDTELSKQIFRVDKNLCLRPMLAPTLYNYARKLD
RILPGPIKIFEVGPCYRKESDGKEHLEEFTMVQFSQMGSGCTRENLEALIKEFLDYLEIDFEIVGDSCMVY
GDTLDIMHGDLELSSAVVGPVSLDREWGIDKPWIGAGFGLERLLKVMHGFKNIKRASRSESYYNGISTN
L*

***M. alvus* AzGGKRS (mutations: M129A, N166Q, V168S, H227 and Y228P)**
MTVKYTDAQIQRLREYGNGTYEQKVFEDLASRDAAFSKEMSVASTDNEKKIKGMIANPSRHGLTQLM
NDIADALVAEGFIEVRTPIFISKDALARMTITEDKPLFKQVFWIDEKRALRPMLAPNLYSVARDLRDHTD
GPVKIFEMGSCFRKESHSGMHLEEFTMLQLSDMGPRGDATEVLKNYISVVMKAAGLPDYDLVQEESD
VYKETIDVEINGQEVCSAAVGPIPLDAAHDVHEPWSGAGFGLERLLTIREKYSTVKKGGASISYLNGAKI
N*

***M. barkeri* wt RS**
MDKKPLDVLISATGLWMSRTGTLHKIKHHEVSRSKIYIEMACGDHLVVNNSRSCRTARAFRHHKYRKT
CKRCRVSDEDINNFLTRSTESKNSVKVRVVSAPKVKKAMPKSVSRAPKPLENSVSAKASTNTSRSVPSP
AKSTPNSSVPASAPAPSLTRSQLDRVEALLSPEDKISLNMAKPFRELEPELVTRRKNDFQRLYTNDREDY
LGKLERDITKFFVDRGFLEIKSPILIPAEYVERMGINNDTELSKQIFRVDKNLCLRPMLAPTLYNYLRKLD
RILPGPIKIFEVGPCYRKESDGKEHLEEFTMVNFCQMGSGCTRENLEALIKEFLDYLEIDFEIVGDSCMVY
GDTLDIMHGDLELSSAVVGPVSLDREWGIDKPWIGAGFGLERLLKVMHGFKNIKRASRSESYYNGISTN
L*

***M. alvus* wt RS**
MTVKYTDAQIQRLREYGNGTYEQKVFEDLASRDAAFSKEMSVASTDNEKKIKGMIANPSRHGLTQLM
NDIADALVAEGFIEVRTPIFISKDALARMTITEDKPLFKQVFWIDEKRALRPMLAPNLMSVMRDLRDHT
DGPVKIFEMGSCFRKESHSGMHLEEFTMLNLVDMGPRGDATEVLKNYISVVMKAAGLPDYDLVQEES
DVYKETIDVEINGQEVCSAAVGPHYLDAAHDVHEPWSGAGFGLERLLTIREKYSTVKKGGASISYLNG
AKIN*

sfGFP-N150TAG-H_6
MPSKGEELFTGVVPILVELDGDVNGHKFSVRGEGEGDATNGKLTLKFICTTGKLPVPWPTLVTTLTYGV
QCFSRYPDHMKRHDFFKSAMPEGYVQERTISFKDDGTYKTRAEVKFEGDTLVNRIELKGIDFKEDGNIL
GHKLEYNFNSH*VYITADKQKNGIKANFKIRHNVEDGSVQLADHYQQNTPIGDGPVLLPDNHYLSTQS
VLSKDPNEKRDHMVLLEFVTAAGITHGMDELYKGSHHHHHH

Ubiquitin-K48TAG-H_6
MQIFVKTLTGKTITLEVEPSDTIENVKAKIQDKEGIPPDQQRLIFAG*QLEDGRTLSDYNIQKESTLHLVL
RLRGGHHHHHH*

SUMO2-K11TAG-H_6
MADEKPKEGV*TENNDHINLKVAGQDGSVVQFKIKRHTPLSKLMKAYCERQGLSMRQIRFRFDGQPIN
ETDTPAQLEMEDEDTIDVFQQQTGGHHHHHH*

H3-K23TAG-H_6
MARTKQTARKSTGGKAPRKQLAT*AARKSAPATGGVKKPHRYRPGTVALREIRRYQKSTELLIRKLPF
QRLVREIAQDFKTDLRFQSSAVMALQEASEAYLVALFEDTNLCAIHAKRVTIMPKDIQLARRIRGERAR
SHHHHHH*

H3-K27TAG-H_6
MARTKQTARKSTGGKAPRKQLATKAAR*SAPATGGVKKPHRYRPGTVALREIRRYQKSTELLIRKLPF
QRLVREIAQDFKTDLRFQSSAVMALQEASEAYLVALFEDTNLCAIHAKRVTIMPKDIQLARRIRGERAR
SHHHHHH*

H3-K79TAG-H_6
MARTKQTARKSTGGKAPRKQLATKAARKSAPATGGVKKPHRYRPGTVALREIRRYQKSTELLIRKLPF
QRLVREIAQDF*TDLRFQSSAVMALQEASEAYLVALFEDTNLCAIHAKRVTIMPKDIQLARRIRGERARS
HHHHHH*

wt Ubiquitin
MQIFVKTLTGKTITLEVEPSDTIENVKAKIQDKEGIPPDQQRLIFAGKQLEDGRTLSDYNIQKESTLHLVL
RLRGG*

Ubiquitin (NGL)
MQIFVKTLTGKTITLEVEPSDTIENVKAKIQDKEGIPPDQQRLIFAGKQLEDGRTLSDYNIQKESTLHLVL
RLNGL

Ubiquitin (AT)
MQIFVKTLTGKTITLEVEPSDTIENVKAKIQDKEGIPPDQQRLIFAGKQLEDGRTLSDYNIQKESTLHLVL
RLALTGG

Ubiquitin (NGLH)
MQIFVKTLTGKTITLEVEPSDTIENVKAKIQDKEGIPPDQQRLIFAGKQLEDGRTLSDYNIQKESTLHLVL
RLNGLH

SUMO2 (NGLH$_6$)
MADEKPKEGVKTENNDHINLKVAGQDGSVVQFKIKRHTPLSKLMKAYCERQGLSMRQIRFRFDGQPIN
ETDTPAQLEMEDEDTIDVFQQQNGLHHHHHH*

TEV-GV-Ubiquitin-K48TAG-H_6 (for double labeling)
MPENLYFQGVGSGGGSMQIFVKTLTGKTITLEVEPSDTIENVKAKIQDKEGIPPDQQRLIFAG*QLEDGR
TLSDYNIQKESTLHLVLRLRGGHHHHHH*

PelB-eGFP-nanobody-wt-H_6
MKYLLPTAAAGLLLLAAQPAMAQVQLVESGGALVQPGGSLRLSCAASGFPVNRYSMRWYRQAPGKE
REWVAGMSSAGDRSSYEDSVKGRFTISRDDARNTVYLQMNSLKPEDTAVYYCNVNVGFEYWGQGTQ
VTVSSKKKHHHHHH*

PelB-eGFP-nanobody-Q12TAG-H_6
MKYLLPTAAAGLLLLAAQPAMAQVQLVESGGALV*PGGSLRLSCAASGFPVNRYSMRWYRQAPGKER
EWVAGMSSAGDRSSYEDSVKGRFTISRDDARNTVYLQMNSLKPEDTAVYYCNVNVGFEYWGQGTQV
TVSSKKKHHHHHH*

PelB-eGFP-nanobody-R18TAG-H_6
MKYLLPTAAAGLLLLAAQPAMAQVQLVESGGALVQPGGSL*LSCAASGFPVNRYSMRWYRQAPGKE
REWVAGMSSAGDRSSYEDSVKGRFTISRDDARNTVYLQMNSLKPEDTAVYYCNVNVGFEYWGQGTQ
VTVSSKKKHHHHHH*

PelB-eGFP-nanobody-R75TAG-H_6
MKYLLPTAAAGLLLLAAQPAMAQVQLVESGGALVQPGGSLRLSCAASGFPVNRYSMRWYRQAPGKE
REWVAGMSSAGDRSSYEDSVKGRFTISRDDA*NTVYLQMNSLKPEDTAVYYCNVNVGFEYWGQGTQ
VTVSSKKKHHHHHH*

PelB-eGFP-nanobody-K86TAG-H_6
MKYLLPTAAAGLLLLAAQPAMAQVQLVESGGALVQPGGSLRLSCAASGFPVNRYSMRWYRQAPGKE
REWVAGMSSAGDRSSYEDSVKGRFTISRDDARNTVYLQMNSL*PEDTAVYYCNVNVGFEYWGQGTQ
VTVSSKKKHHHHHH*

PelB-eGFP-nanobody-K116TAG-H_6
MKYLLPTAAAGLLLLAAQPAMAQVQLVESGGALVQPGGSLRLSCAASGFPVNRYSMRWYRQAPGKE
REWVAGMSSAGDRSSYEDSVKGRFTISRDDARNTVYLQMNSLKPEDTAVYYCNVNVGFEYWGQGTQ
VTVSSK*KHHHHHH*

PelB-eGFP-nanobody-X120TAG-H_6
MKYLLPTAAAGLLLLAAQPAMAQVQLVESGGALVQPGGSLRLSCAASGFPVNRYSMRWYRQAPGKE
REWVAGMSSAGDRSSYEDSVKGRFTISRDDARNTVYLQMNSLKPEDTAVYYCNVNVGFEYWGQGTQ
VTVSSKKKGG*GSHHHHHH*

GST
MSPILGYWKIKGLVQPTRLLLEYLEEKYEEHLYERDEGDKWRNKKFELGLEFPNLPYYIDGDVKLTQS
MAIIRYIADKHNMLGGCPKERAEISMLEGAVLDIRYGVSRIAYSKDFETLKVDFLSKLPEMLKMFEDRL
CHKTYLNGDHVTHPDFMLYDALDVVLYMDPMCLDAFPKLVCFKKRIEAIPQIDKYLKSSKYIAWPLQG
WQATFGGGDHPPKGIE*

GST-eGFP
MSPILGYWKIKGLVQPTRLLLEYLEEKYEEHLYERDEGDKWRNKKFELGLEFPNLPYYIDGDVKLTQS
MAIIRYIADKHNMLGGCPKERAEISMLEGAVLDIRYGVSRIAYSKDFETLKVDFLSKLPEMLKMFEDRL
CHKTYLNGDHVTHPDFMLYDALDVVLYMDPMCLDAFPKLVCFKKRIEAIPQIDKYLKSSKYIAWPLQG
WQATFGGGDHPPKGIEGGGSMVSKGEELFTGVVPILVELDGDVNGHKFSVSGEGEGDATYGKLTLKFI
CTTGKLPVPWPTLVTTLTYGVQCFSRYPDHMKQHDFFKSAMPEGYVQERTIFFKDDGNYKTRAEVKFE
GDTLVNRIELKGIDFKEDGNILGHKLEYNYNSHNVYIMADKQKNGIKVNFKIRHNIEDGSVQLADHYQ
QNTPIGDGPVLLPDNHYLSTQSALSKDPNEKRDHMVLLEFVTAAGITLGMDELYK*

GST-UBE2R1 (Cdc34)
MSPILGYWKIKGLVQPTRLLLEYLEEKYEEHLYERDEGDKWRNKKFELGLEFPNLPYYIDGDVKLTQS
MAIIRYIADKHNMLGGCPKERAEISMLEGAVLDIRYGVSRIAYSKDFETLKVDFLSKLPEMLKMFEDRL
CHKTYLNGDHVTHPDFMLYDALDVVLYMDPMCLDAFPKLVCFKKRIEAIPQIDKYLKSSKYIAWPLQG
WQATFGGGDHPPKSDLEVLFQGPLGSARPLVPSSQKALLLELKGLQEEPVEGFRVTLVDEGDLYNWEV
AIFGPPNTYYEGGYFKARLKFPIDYPYSPPAFRFLTKMWHPNIYETGDVCISILHPPVDDPQSGELPSERW
NPTQNVRTILLSVISLLNEPNTFSPANVDASVMYRKWKESKGKDREYTDIIRKQVLGTKVDAERDGVK
VPTTLAEYCVKTKAPAPDEGSDLFYDDYYEDGEVEEEADSCFGDDEDDSGTEES*

Srt2A-TEV-H_6
MQAKPQIPKDKSKVAGYIEIPDADIKEPVYPGPATREQLNRGVCFHDENESLDDQNISIAGHTFIDRPNY
QFTNLKAAKPGSMVYFKVGNETRIYKMTSIRKVHPNAVEVLDEQEGKDKQLTLVTCDDYNEETGVWE
SRKIFVATEVKGSENLYFQGHHHHHH*

H_6-Thrombin-USP2
MGSSHHHHHHSSGLVPRGSSSPGRDGMNSKSAQGLAGLRNLGNTCFMNSILQCLSNTRELRDYCLQRL
YMRDLHHGSNAHTALVEEFAKLIQTIWTSSPNDVVSPSEFKTQIQRYAPRFVGYNQQDAQEFFRFLLDG
LHNEVNRVTLRPKSNPENLDHLPDDEKGRQMWRKYLEREDSRIGDLFVGQLKSSLTCTDCGYCSTVFD
PFWDLSLPIAKRGYPEVTLMDCMRLFTKEDVLDGDAAPTCCRCRGRKRCIKKFSIQRFPKILVLHLKRFS
ESRIRTSKLTTFVNFPLRDLDLREFASENTNHAVYNLYAVSNHSGTTMGGHYTAYCRSPGTGEWHTFN
DSSVTPMSSSQVRTSDAYLLFYELASPPSRM*

Mammalian constructs:

Lifeact-mEGFP (Addgene 54610)
MGVADLIKKFESISKEEGDPPVATMVSKGEELFTGVVPILVELDGDVNGHKFSVSGEGEGDATYGKLTL
KFICTTGKLPVPWPTLVTTLTYGVQCFSRYPDHMKQHDFFKSAMPEGYVQERTIFFKDDGNYKTRAEV
KFEGDTLVNRIELKGIDFKEDGNILGHKLEYNYNSHNVYIMADKQKNGIKVNFKIRHNIEDGSVQLADH
YQQNTPIGDGPVLLPDNHYLSTQSKLSKDPNEKRDHMVLLEFVTAAGITLGMDELYK*

mSA-EGFP-TM (Addgene 39863)
METDTLLLWVLLLWVPGSTGDYPYDVPDYAGAQPARSMAEAGITGTWYNQSGSTFTVTAGADGNLT
GQYENRAQGTGCQNSPYTLTGRYNGTKLEWRVEWNNSTENCHSRTEWRGQYQGGAEARINTQWNLT
YEGGSGPATEQGQDTFTKVKPSAASGSDYKDDDDKGAPMVSKGEELFTGVVPILVELDGDVNGHKFS
VSGEGEGDATYGKLTLKFICTTGKLPVPWPTLVTTLTYGVQCFSRYPDHMKQHDFFKSAMPEGYVQER
TIFFKDDGNYKTRAEVKFEGDTLVNRIELKGIDFKEDGNILGHKLEYNYNSHNVYIMADKQKNGIKVNF
KIRHNIEDGSVQLADHYQQNTPIGDGPVLLPDNHYLSTQSALSKDPNEKRDHMVLLEFVTAAGITLGM
DELYKRPRLQVDEQKLISEEDLNAVGQDTQEVIVVPHSLPFKVVVISAILALVVLTIISLIILIMLWQKKPR
*

1. General methods: Plasmids and reagents

Codon optimized H_6-Ubiquitin-*Oa*AEP1b (C247A) was purchased as a DNA String (GeneArt, Thermo Fisher) and cloned into the pET29b vector *via* standard restriction cloning (see Supplementary Table 3). Point mutations, insertions and deletions were introduced using Site-directed, Ligase-Independent Mutagenesis (SLIM, see Supplementary Table 1 and 3).[1] Srt2A in pET29b (Addgene plasmid #75145), mEGFP-Lifeact-7 (Addgene plasmid #54610) and pDisplay-mSA-EGFP-TM (Addgene plasmid #39863) were purchased. Oligonucleotide primers were designed with NEBuilder and purchased from Sigma Aldrich or Microsynth (see Supplementary Tables 1 and 2). Amino acid sequences of all proteins are listed in Supplementary Note 1.

All solvents and chemical reagents were purchased from Sigma Aldrich, Carbolution, Acros Organics or Fisher Scientific and were used without further purification unless stated otherwise. 15 % SDS-PAGE gels were run (170 V for 60 min) on a Bolt™ Mini Gel Tank system (Invitrogen). Gels were stained with Quick Coomassie Stain (Generon). Color Prestained Protein Standard, Broad Range 11-245 kDa (NEB), Broad Range 10-250 kDa (NEB) or PageRuler Prestained Plus Protein Ladder 10-250 kDa (ThermoFisher) were used as the protein marker. Protein and DNA concentrations were measured on a NanoPhotometer® NP60 (Implen). H_6-UBE1 was purchased from Boston Biochem (Cat. No. E-304-050). Ni-NTA agarose was purchased from Jena Bioscience (Cat. No. AC-501). In-gel fluorescence was captured using an iBright FL1500 imaging system (Thermo Fisher Scientific). N^6-((2-azidoacetyl)glycyl)-L-lysine (Azido-GGisoK or AzGGisoK) was synthesized *via* solid-phase peptide synthesis (SPPS) as previously described.[2]

2. Chemical synthesis

General synthetic procedures for solid-phase peptide synthesis (SPPS)

GGisoK, GVisoK, GLisoK, dtb-NGL, dtb-NGLH, alkyne-NGLH, SuRho-NGLH and Fmoc-$(G)_6$-NGLH were synthesized via solid-phase peptide synthesis (SPPS). SPPS was performed in a custom-made glass apparatus with a frit for larger amounts of resin or in plastic syringes with a frit for small amounts (< 1 g). Shaking was performed manually or by using a rotary unit. The equivalents (eq.) used were based on the maximal loading capacity of the CTC resin given by the supplier.

Loading and capping of CTC resin

SPPS was performed according to the Fmoc-strategy for solid-phase synthesis using CTC-resin. Therefore, 1.2 eq. of Fmoc-protected amino acid and 2.5 eq. of DIPEA were dissolved in anhydrous DCM (10 mL/g resin) and then added to 1.0 eq. of CTC-resin (100-200 mesh, 1.0-1.6 mmol/g maximal loading capacity) in a syringe equipped with a frit. The mixture was allowed to shake for 1 h at RT. Capping of the remaining chlorotrityl-groups was performed by adding 3 eq. of MeOH and 2.5 eq. DIPEA to the syringe followed by 15 min of shaking at RT. Subsequently, the resin was washed 5 times with DCM and 5 times with DMF. The loading capacity of the resin was determined by mass gain after washing the resin with MeOH and drying in high vacuum.

On-resin Fmoc-deprotection

Removal of the Fmoc-protection group was performed by adding a solution of 20 % piperidine in DMF to the resin in the syringe, followed by 5 min of shaking at RT. This procedure was repeated a second time for 10 min. The resin was subsequently washed 5 times with DMF.

On-resin coupling of amino acids

For the coupling of amino acids, a 0.1 - 0.2 M solution of the Fmoc-protected amino acid (2 eq.), HATU (2 eq.), and DIPEA (5 eq.) was prepared in DMF and stirred for 5 min at RT. Afterwards, the syringe/reactor containing the Fmoc-deprotected resin-bound free amino acid was incubated with this solution at RT for 45 min. Subsequently, the resin was washed 5 times with DMF.

Cleavage from the CTC resin

The resin was washed 5 times with DMF and 5 times with DCM and then treated with a solution of 20 % HFIP in DCM (v/v) at RT for 10 min. The filtrate was collected and the procedure was repeated twice. Afterwards, the resin was washed three times with DCM. The filtrates were combined and the solvent was evaporated under reduced pressure.

Removal of acid-labile protection groups

For the removal of acid-labile protection groups the product was dissolved in a mixture of TFA/DCM/TIPS/H_2O (90/5/2.5/2.5, v/v/v/v) and stirred at RT for 1 h. Subsequently, the solvents were removed under reduced pressure followed by co-evaporation with toluene.

Precipitation with Et_2O

After removal of the solvents the product was added dropwise into a centrifugal tube containing ice-cold Et_2O. After centrifugation, the precipitate was washed twice with ice-cold Et_2O and centrifuged again. The resulting precipitate was dissolved in H_2O and lyophilized.

Synthesis of GVisoK, GLisoK and GGisoK

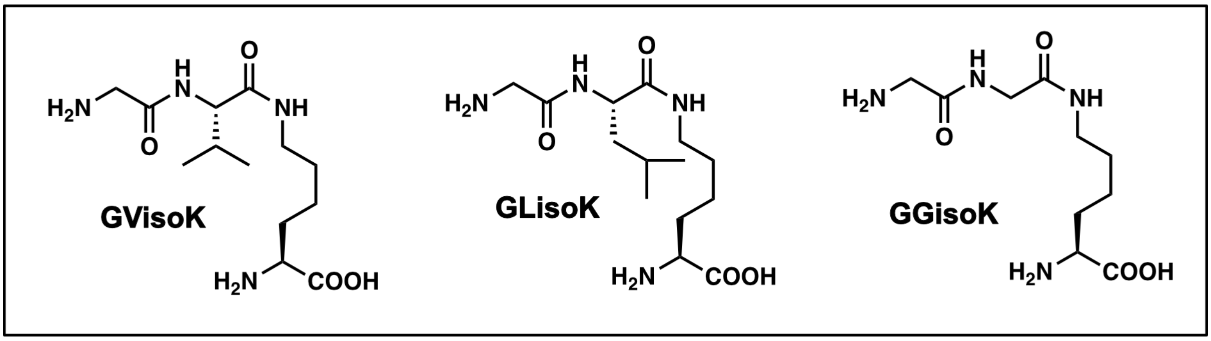

Scheme S1: *Chemical structures of GVisoK, GLisoK and GGisoK.*

For the synthesis of the tripeptides GVisoK, GLisoK and GGisoK, 0.5 g of CTC-resin (1.0 mmol/g maximal loading capacity) were used. The loading of the resin with Boc-Lys(Fmoc)-OH and all the coupling steps with the Fmoc-protected amino acids were performed as described in the standard Fmoc SPPS protocol above. After coupling of Boc-Gly-OH, the product was cleaved from the resin by charging the syringe with a solution of 20 % HFIP in DCM (v/v) followed by shaking at RT for 20 min. This procedure was repeated and the filtrates were combined in a round bottom flask. After removal of the acid-labile Boc-protection group the tripeptides were precipitated and lyophilized as described above. Lyophilization yielded GVisoK with 68.4 % yield (0.181 g), GLisoK with 65.1 % yield (0.174 g) and GGisoK with 53.4 % yield (0.130 g, all double TFA-salts, calculated using the maximal loading capacity as 100 %). GVisoK, GLisoK and GGisoK were dissolved in H_2O and stored as 100 mM stock solutions at -20 °C.

GVisoK: MS (ESI+) *m/z* 303.2 $[M+H]^+$; Calculated for $C_{13}H_{26}N_4O_4^+$: 303.2 $[M+H]^+$

GLisoK: MS (ESI+) *m/z* 317.2 $[M+H]^+$; Calculated for $C_{14}H_{28}N_4O_4^+$: 317.2 $[M+H]^+$

GGisoK: MS (ESI+) *m/z* 261.1 $[M+H]^+$; Calculated for $C_{10}H_{20}N_4O_4^+$: 261.2 $[M+H]^+$

Synthesis of desthiobiotin-NGL (dtb-NGL)

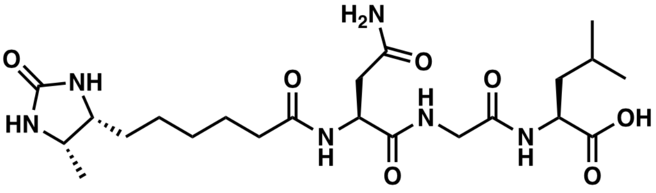

Scheme S2: *Chemical structure of dtb-NGL.*

For the synthesis of dtb-NGL, 0.25 g of CTC-resin (1.0 mmol/g maximal loading capacity) were used. The loading of the resin with Fmoc-Leu-OH and all the coupling steps with the Fmoc-protected amino acids were performed as described in the standard Fmoc SPPS protocol above. After coupling of Fmoc-Asn(Trt)-OH, the Fmoc protection group was removed as described above and desthiobiotin (dtb) was coupled using dtb (3 eq.), HATU (2.5 eq.) and DIPEA (4 eq.) in DMF for 2 h at RT. For cleavage, the syringe was charged with a solution of 20 % HFIP in DCM (v/v) followed by shaking at RT for 20 min. This procedure was repeated and the filtrates were combined in a round bottom flask. After removal of the acid-labile protection groups dtb-NGL was precipitated and lyophilized as described above. Preparative HPLC purification yielded dtb-NGL as white powder with 44.1 % yield (0.055 g). dtb-NGL was dissolved in DMSO and stored as a 200 mM stock solution at -20 °C.

dtb-NGL: MS (ESI+) *m/z* 499.3 $[M+H]^+$; Calculated for $C_{22}H_{38}N_6O_7^+$: 499.3 $[M+H]^+$

Synthesis of desthiobiotin-NGLH (dtb-NGLH)

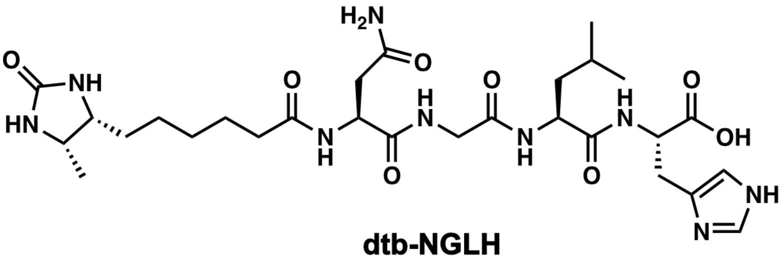

Scheme S3: *Chemical structure of dtb-NGLH.*

dtb-NGLH was synthesized analogously to dtb-NGL but Fmoc-His(Trt)-OH was used for charging of CTC resin instead of Fmoc-Leu-OH. After removal of the acid-labile protection groups dtb-NGLH was precipitated and lyophilized as described above. Preparative HPLC purification yielded dtb-NGLH as white powder with 50.9 % yield (0.081 g). dtb-NGLH was dissolved in DMSO and stored as a 200 mM stock solution at -20 °C.

dtb-NGLH: MS (ESI+) *m/z* 636.3 $[M+H]^+$; Calculated for $C_{28}H_{45}N_9O_8^+$: 636.3 $[M+H]^+$

Synthesis of alkyne-NGLH

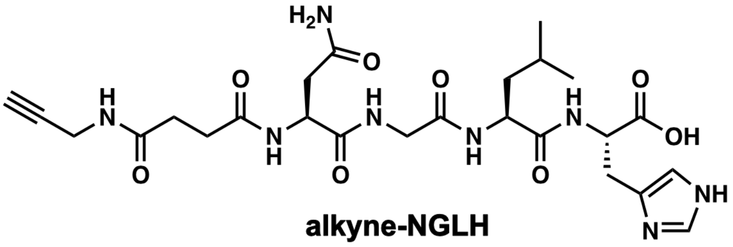

Scheme S4: *Chemical structure of alkyne-NGLH.*

Alkyne-NGLH was synthesized analogously to dtb-NGLH. After coupling of Fmoc-Asn(Trt)-OH, the Fmoc protection group was removed as described above and the syringe was charged with succinic anhydride (4 eq.) and DIPEA (2 eq.) dissolved in DMF. After incubation for 30 min at RT the syringe was washed 5 times with DMF. Afterwards a solution of HATU (2 eq.) and DIPEA (3 eq.) in DMF was added to the syringe followed by incubation for 10 min at RT. After washing 5 times with DMF a solution of propargylamine (5 eq.) and DIPEA (2 eq.) in DMF was added to the syringe for 1 h at RT, followed by 5 DMF and 5 DCM washes. For cleavage, the syringe was charged with a solution of 20 % HFIP in DCM (v/v) followed by

shaking at RT for 20 min. This procedure was repeated and the filtrates were combined in a round bottom flask. After removal of the acid-labile protection groups alkyne-NGLH was precipitated and lyophilized as described above. Preparative HPLC purification yielded alkyne-NGLH as white powder with 34.7 % yield (0.04 g). Alkyne-NGLH was dissolved in H_2O and stored as a 200 mM stock solution at -20 °C.

alkyne-NGLH: MS (ESI+) *m/z* 577.3 $[M+H]^+$; Calculated for $C_{25}H_{36}N_8O_8^+$: 577.3 $[M+H]^+$

Synthesis of Sulforhodamine B-NGLH (SuRho-NGLH)

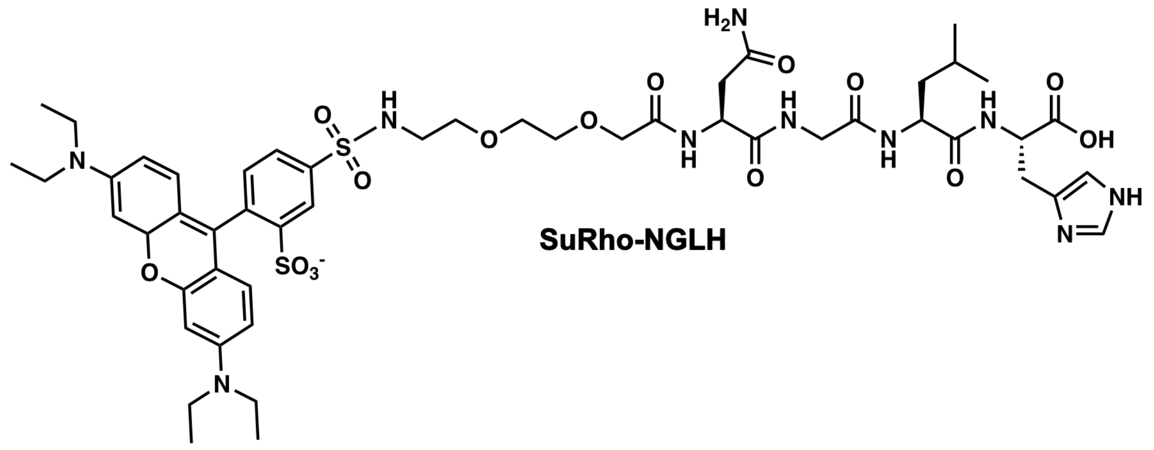

Scheme S4: *Chemical structure of SuRho-NGLH.*

SuRho-NGLH was synthesized analogously to dtb-NGLH. After coupling of Fmoc-Asn(Trt)-OH, the Fmoc protection group was removed as described above and the syringe was charged with Fmoc-NH-(PEG)$_2$-COOH (2 eq.), HATU (2 eq.) and DIPEA (2 eq.) dissolved in DMF. After incubation for 1 h at RT the syringe was washed 5 times with DMF followed by Fmoc deprotection. Afterwards, a solution of Sulforhodamine B acid chloride (1 eq.) and DIPEA (3 eq.) in DMF was added to the syringe followed by incubation for 2 h at RT as well as 5 DMF and 5 DCM washes. For cleavage, the syringe was charged with a solution of 20 % HFIP in DCM (v/v) followed by shaking at RT for 20 min. This procedure was repeated and the filtrates were combined in a round bottom flask. After removal of the acid-labile protection groups SuRho-NGLH was precipitated and lyophilized as described above. Preparative HPLC purification yielded SuRho-NGLH as pink powder with 15.3 % yield (0.043 g). SuRho-NGLH was dissolved in DMSO and stored as a 200 mM stock solution at -20 °C.

SuRho-NGLH: MS (ESI+) *m/z* 563.4 $[M+2H]^{2+}$; Calculated for $C_{51}H_{70}N_{10}O_{15}S_2^{2+}$: 563.7 $[M+2H]^{2+}$

Synthesis of Fmoc-(G)$_6$-NGLH

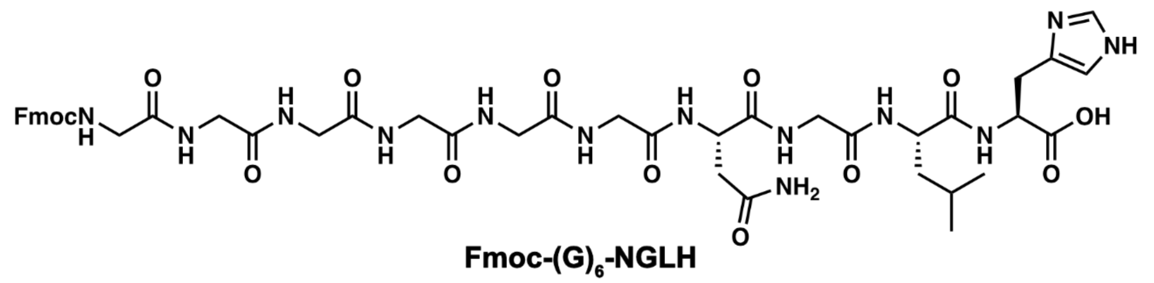

Scheme S5: *Chemical structure of Fmoc-(G)$_6$-NGLH.*

Fmoc-(G)$_6$-NGLH was synthesized analogously to dtb-NGLH. After coupling of Fmoc-Asn(Trt)-OH, the Fmoc protection group was removed as described above and the syringe was charged with Fmoc-Gly-Gly-Gly-COOH (2 eq.), HATU (2 eq.) and DIPEA (4 eq.) dissolved in DMF. After incubation for 1 h at RT the syringe was washed 5 times with DMF followed by Fmoc deprotection and repetition of the Fmoc-Gly-Gly-Gly-COOH coupling step. After 5 DMF and 5 DCM washes the syringe was charged with a solution of 20 % HFIP in DCM (v/v) followed by shaking at RT for 20 min. This procedure was repeated and the filtrates were combined in a round bottom flask. After removal of the acid-labile protection groups Fmoc-(G)$_6$-NGLH was precipitated and lyophilized as described above. Preparative HPLC purification yielded Fmoc-(G)$_6$-NGLH as white powder with 12.6 % yield (0.019 g). Fmoc-(G)$_6$-NGLH was dissolved in DMSO and stored as a 200 mM stock solution at -20 °C.

Fmoc-(G)$_6$-NGLH: MS (ESI+) *m/z* 502.8 $[M+2H]^{2+}$; Calculated for $C_{45}H_{59}N_{13}O_{14}^{2+}$: 502.7 $[M+2H]^{2+}$

3. Protein expression and purification

Preparation of GGisoK-bearing POIs

Chemically competent *E. coli* K12 cells were cotransformed with pPylT_POI (encoding *Mb/Ma* $tRNA_{CUA}$ and the C-terminally H_6-tagged POI with a TAG codon at the denoted position (see Supplementary Table 3)), and pBK_aaRS (encoding the *Mb/Ma* PylRS) plasmids. After recovery with 1 mL of SOC medium for 1 h at 37 °C, the cells were cultured overnight in 50 mL of non-inducing medium[3] supplemented with tetracycline (17.5 µg/mL) and ampicillin (100 µg/mL) at 37 °C, 200 rpm. The overnight culture was diluted to an OD_{600} of 0.05 in autoinduction medium[3] containing antibiotics (tetracycline (8.75 µg/mL) and ampicillin (50 µg/mL)) and the corresponding non-canonical amino acid (either 2 mM BocK or 4 mM AzGGisoK). After overnight incubation at 37 °C the cells were harvested by centrifugation (4000 x g, 20 min, 4 °C), flash frozen in liquid nitrogen and stored at -80 °C. The obtained cell pellets were thawed on ice, resuspended in lysis buffer (50 mM Tris pH 8.0, 300 mM NaCl, 0.1 mg/mL DNase I (AppliChem), one cOmplete™ protease inhibitor tablet (Roche) and 1 mM PMSF) and, after incubation on ice for 30 min, subjected to ultrasonication in an ice-water bath. Lysed cells were centrifuged (15 000 x g, 40 min, 4 °C) and the cleared lysate was added to 1 mL Ni-NTA slurry / 1 L culture (Jena Bioscience) equilibrated with wash buffer (20 mM Tris pH 8.0, 300 mM NaCl and 30 mM imidazole). Afterwards, the mixture was incubated with agitation for 1 h at 4 °C, transferred to an empty plastic column and washed with 10 column volumes (CV) of wash buffer. The protein was eluted in 1 mL fractions using wash buffer supplemented with 300 mM imidazole pH 8.0. Fractions containing the POI (identified *via* SDS-PAGE) were pooled, concentrated and rebuffered (50 mM Tris pH 7.5 and 150 mM NaCl) using Amicon® centrifugal filter units (Millipore) with a suitable molecular weight cut-off (MWCO). Protein concentration was calculated from the measured A280 absorption (extinction coefficients were calculated with ProtParam (https://web.expasy.org/protparam/)). In case of Ub and SUMO the determination of protein concentration using the absorption at 280 nm is inaccurate (due to their low extinction coefficient (ε)), so BCA (Thermo Scientific) and Bradford (Sigma Aldrich) assays were used to determine the accurate protein concentration. POIs with incorporated ncAAs were flash frozen in liquid nitrogen and stored at -80 °C until further use.

Preparation of GGisoK-bearing H3 variants

Chemically competent *E. coli* K12 cells were cotransformed with pBAD_H3-KxxTAG-H_6 encoding the C-terminally H_6-tagged H3 wildtype or with a TAG codon at the denoted position (see Supplementary Table 3), and pEVOL_AzGGKRS (encoding the *Mb* PylRS and the *Mb*

tRNA$_{CUA}$) plasmids. After recovery with 1 mL of SOC medium for 1 h at 37 °C, the cells were cultured overnight in 50 mL of non-inducing medium[3] supplemented with chloramphenicol (50 µg/mL) and ampicillin (100 µg/mL) at 37 °C, 200 rpm. The overnight culture was diluted to an OD$_{600}$ of 0.05 in autoinduction medium[3] containing antibiotics (chloramphenicol (25 µg/mL) and ampicillin (50 µg/mL)) and the non-canonical amino acid AzGGK (4 mM). After overnight incubation at 37 °C the cells were harvested by centrifugation (4000 x g, 20 min, 4 °C), flash frozen in liquid nitrogen and stored at -80 °C. The obtained cell pellets were thawed on ice, resuspended in lysis buffer (50 mM Tris pH 8.0, 500 mM NaCl) and, after incubation on ice for 30 min, subjected to ultrasonication in an ice-water bath. Lysed cells were centrifuged (15 000 x g, 20 min, 4 °C) and the supernatant was discarded followed by two washing steps of the insoluble fraction with 2 % Triton (15 000 x g, 10 min, 4 °C). After an additional washing step with lysis buffer, the pellet was resuspended in lysis buffer supplemented with 6 M Urea followed by sonication and centrifugation (15 000 x g, 10 min, 4 °C). In a first step, the supernatant containing a H3 variant was purified using a HisTrap FF (Cytiva) with a linear gradient (Buffer A: 50 mM Tris pH 8.0, 500 mM NaCl, 6 M Urea, 30 mM Imidazol; Buffer B: 50 mM Tris pH 8.0, 500 mM NaCl, 6 M Urea, 300 mM Imidazol). Fractions containing H3 (identified *via* SDS-PAGE) were pooled and subjected to cation exchange chromatography using a Resource S column (Cytiva) with a gradient from 5 % - 40 % B (Buffer A: 50 mM Tris pH 7.5, 100 mM NaCl, 6 M Urea, 1 mM DTT, Buffer B: 50 mM Tris pH 7.5, 1 M NaCl, 6 M Urea, 1 mM DTT). Fractions containing H3 (identified *via* SDS-PAGE) were pooled and refolded by stepwise dialysis (6 M Urea to 0 M Urea in six steps) using Pur-A-Lyzer™ (Sigma-Aldrich) dialysis chambers with a 3.5 kDa cutoff at 4 °C into Histone storage buffer (50 mM Tris pH 7.5, 150 mM NaCl, 0.5 mM TCEP). Finally, H3 variants were concentrated using Amicon® centrifugal filter units (Millipore) with a 3 kDa molecular weight cut-off (MWCO). Protein concentration was calculated from the measured A280 absorption (extinction coefficients were calculated with ProtParam (https://web.expasy.org/protparam/)). H3 variants were flash frozen in liquid nitrogen and stored at -80 °C until further use.

Usual yields of amber suppressed POIs per litre culture were as follows:
Ub-K48GGisoK: 3-5 mg/L
H3-K23/27/79GGisoK: 4-6 mg/L
SUMO-K11GGisoK: 5-8 mg/L

Expression, purification and activation of *Oa*AEP1 (C247A)

Apart from a few adaptations, recombinant *Oa*AEP1 (C247A) was produced as previously described.[4] Chemically competent *E. coli* T7 Shuffle cells (NEB) were transformed with pET29_His$_6$_ubiquitin_*Oa*AEP1_C247A plasmid (see Supplementary Table 3). After recovery with 1 mL of SOC medium for 1 h at 37 °C, the cells were cultured overnight in 50 mL of 2x YT medium containing kanamycin (50 µg/mL) at 37 °C, 200 rpm. The overnight culture was diluted to an OD_{600} of 0.05 in 1 L of fresh 2xYT medium supplemented with kanamycin (25 µg/ml) and cultured at 37 °C while shaking (200 rpm) until OD_{600} = 0.5 - 0.8 was reached. IPTG was added to a final concentration of 0.4 mM followed by incubation at 16 °C overnight. Cells were harvested by centrifugation (4000 x g, 20 min, 4 °C) and lysed by ultrasonication in *Oa*AEP1 lysis buffer (100 mM Na_2HPO_4 / NaH_2PO_4 pH 7.4, 150 mM NaCl, 2 mM DTT, cOmplete^{TM} protease inhibitor (Roche), 1 mM PMSF). The lysate was cleared by centrifugation (14 000 x g, 25 min, 4 °C). Washed NiNTA agarose (Jena Bioscience) was added for Ni-NTA purification and incubated at 4 °C for 1 h. The mixture was transferred to an empty plastic column, washed with 10 CV of wash buffer (100 mM Na_2HPO_4 / NaH_2PO_4 pH 7.4, 150 mM NaCl, 30 mM imidazole) and the protein was subsequently eluted with elution buffer (washing buffer supplemented with 300 mM imidazole pH 8.0). *Oa*AEP1 activation was performed by dialysis against 50 mM sodium acetate pH 4.0 and 1 mM EDTA overnight at RT. The solution was cleared by centrifugation (14 000 x g, 25 min, 4 °C) and subjected to cation exchange chromatography. A HiTrap SP FF 5 mL column (GE) was loaded and washed with 10 column volumes (CV) sodium acetate pH 4.0. *Oa*AEP1 was eluted using a NaCl gradient (1.5 CV, 1 % to 100 %, 50 mM sodium acetate pH 4.0, 1 M NaCl). The protein was concentrated, rebuffered into *Oa*AEP1 storage buffer (100 mM Na_2HPO_4 / NaH_2PO_4 pH 7.4, 150 mM NaCl, 2 mM DTT, 10% glycerol), aliquoted and stored at -80 °C. Under these conditions the activated protein was used for several months without loss of activity.

Expression and purification of sortase 2A

Chemically competent *E. coli* BL21 (DE3) were transformed with pET29b_Srt2A-TEV-H$_6$ plasmid (see Supplementary Table 3). After recovery with 1 mL of SOC medium for 1 h at 37 °C, the cells were cultured overnight in 50 mL of 2xYT medium containing kanamycin (50 µg/mL) at 37 °C, 200 rpm. The overnight culture was diluted to an OD_{600} of 0.05 in 3 L of fresh 2xYT medium supplemented with kanamycin (25 µg/mL) and cultured at 37 °C while shaking (200 rpm) until OD_{600} = 0.5 - 0.8 was reached. IPTG was added to a final concentration of 0.4 mM and protein expression was induced for 3 h at 30 °C. The cells were harvested by

centrifugation (4000 x g, 20 min, 4 °C) and resuspended in lysis buffer (50 mM Tris pH 8.0, 300 mM NaCl supplemented with 1 mM $MgCl_2$, 0.1 mg/mL DNase I (AppliChem), one cOmplete^{TM} protease inhibitor tablet (Roche) and 1 mM PMSF). Cells were lysed by sonication in an ice-water bath and centrifuged (15 000 x g, 40 min, 4 °C). The cleared lysate was added to Ni-NTA slurry (Jena Bioscience, 1 mL of slurry per 1 L culture) and equilibrated with wash buffer (20 mM Tris pH 8.0, 300 mM NaCl and 30 mM imidazole). Subsequently, the mixture was incubated with agitation for 1 h at 4 °C, transferred to an empty plastic column and washed with 10 CV of wash buffer. The protein was eluted in 1 mL fractions using wash buffer supplemented with 300 mM imidazole pH 8.0. In order to cleave off the H_6-tag, protein-containing fractions were pooled and 200 µL of TEV protease (1.8 mg/mL) were added. The mixture was transferred to a dialysis tubing (Roth) and the dialysis bag was immersed in 2 L of cold dialysis buffer (25 mM Tris pH 8.0, 150 mM NaCl, 0.5 mM DTT) and stirred at 4 °C overnight. The protein mixture was recovered from the dialysis tubing and centrifuged (15 000 xg, 10 min, 4 °C) in order to precipitate TEV protease. Equilibrated Ni-NTA slurry was added to the supernatant and the mixture was incubated with agitation for 1 h at 4 °C. The mixture was then poured into an empty plastic column and the flow-through was collected. The Ni-NTA beads were washed twice with 15 mL of wash buffer (20 mM Tris pH 8.0, 150 mM NaCl and 5 mM $CaCl_2$). Flow-through and wash fractions containing purified sortase without H_6-tag were pooled, concentrated and rebuffered (50 mM Tris pH 7.5, 150 mM NaCl, 5 mM $CaCl_2$) using Amicon® Ultra-4 10K MWCO centrifugal filter units (Millipore). Enzyme concentration was calculated from the measured A280 absorption (extinction coefficients were calculated with ProtParam (https://web.expasy.org/protparam/)). Sortase 2A was flash frozen in liquid nitrogen and stored at -80 °C until further use.

Expression and purification of tagless ubiquitin and ubiquitin variants

Chemically competent *E. coli* Rosetta2 (DE3) were transformed with pET17b-ubiquitin plasmid (see Supplementary Table 3). After recovery with 1 mL of SOC medium for 1 h at 37 °C, the cells were cultured overnight in 50 mL of 2xYT medium containing ampicillin (100 µg/mL) and chloramphenicol (50 µg/mL) at 37 °C, 200 rpm. The overnight culture was diluted to an OD_{600} of 0.05 in 3 L of fresh 2xYT medium supplemented with ampicillin (50 µg/mL) and chloramphenicol (25 µg/mL) and cultured at 37 °C while shaking (200 rpm) to OD_{600} = 0.8 - 1.0. IPTG was added to a final concentration of 1 mM and protein expression was induced for 4 h at 37 °C. Cells were harvested by centrifugation (4000 x g, 20 min, 4 °C), resuspended in lysis buffer (50 mM Tris pH 7.6, supplemented with 10 mM $MgCl_2$, 1 mM EDTA, 0.1 % NP-

40, 0.1 mg/mL DNAse I, one cOmplete^{TM} protease inhibitor tablet and 1 mM PMSF), lysed by ultrasonication in an ice-water bath and centrifuged (15 000 x g, 40 min, 4 °C).
The cleared lysate was transferred into a glass beaker in an ice-water bath that was placed on a magnetic stirrer. Precipitation was performed with 35 % perchloric acid until pH 4.0 – 4.5 was reached. After 5 min incubation at 4 °C while stirring, the milky solution was centrifuged (15 000 x g, 40 min, 4 °C) and the supernatant was transferred into a dialysis tubing with a MWCO of 2 kDa (Roth). Dialysis was performed overnight at 4 °C with 50 mM ammonium acetate buffer pH 4.5. The dialysed solution was centrifuged (15 000 x g, 40 min, 4 °C), filtered and purified *via* HiTrap SP FF 5 mL cation exchange chromatography (GE, gradient 0 – 1 M NaCl). Fractions that showed > 95 % purity, as judged by SDS-PAGE, were pooled and rebuffered (50 mM Tris pH 8.0, 150 mM NaCl, 5 mM $CaCl_2$) using Amicon® Ultra-15 3 kDa MWCO centrifugal filter units (Millipore). BCA (Thermo Scientific™) and Bradford (Sigma Aldrich) assays were used for protein concentration determination. All Ub variants were flash frozen in liquid nitrogen and stored at -80 °C until further use.

Expression and purification of H_6-tagged SUMO(NGLH_6)

Chemically competent *E. coli* Rosetta2 (DE3) were transformed with H_6-tagged SUMO(NGLH_6) in a pET17b plasmid (see Supplementary Table 3). After recovery with 1 mL of SOC medium for 1 hour at 37 °C, the cells were cultured overnight in 50 mL of 2xYT medium containing ampicillin (100 µg/mL) and chloramphenicol (50 µg/mL) at 37 °C, 200 rpm. The overnight culture was diluted to an OD_{600} of 0.05 in 3 L of fresh 2xYT medium supplemented with ampicillin (50 µg/mL) and chloramphenicol (25 µg/mL) and cultured at 37 °C, 200 rpm, to OD_{600} = 0.8 - 1.0. IPTG was added to a final concentration of 1 mM and protein expression was induced for 4 h at 37 °C. Cells were harvested by centrifugation (4000 x g, 20 min, 4 °C), resuspended in 20 mL of lysis buffer / 1 L of culture (20 mM Tris pH 8.0, 300 mM NaCl, 30 mM imidazole, 1 mM PMSF, 0.1 mg/mL DNase I and one cOmplete^{TM} protease inhibitor tablet (Roche)), incubated on ice for 30 min and sonicated in an ice-water bath. Lysed cells were centrifuged (15 000 x g, 40 min, 4 °C), the cleared lysate added to Ni-NTA slurry (Jena Bioscience, 1 mL of slurry per 1 L of culture) an incubated with agitation for 1 h at 4 °C. The mixture was then transferred to an empty plastic column and washed with 10 CV of wash buffer (20 mM Tris pH 8.0, 300 mM NaCl and 30 mM imidazole pH 8.0). The protein was eluted in 1 mL fractions with wash buffer supplemented with 300 mM imidazole pH 8.0. The protein-containing fractions were pooled, concentrated and rebuffered (50 mM Tris pH 7.5, 150 mM NaCl, 5 mM $CaCl_2$) using Amicon® centrifugal filter units with a 3 kDa MWCO

(Millipore). Purified proteins were analyzed by SDS-PAGE and mass spectrometry. BCA (Thermo Scientific™) and Bradford (Sigma Aldrich) assays were used for protein concentration determination. SUMO(NGLH$_6$) was flash frozen in liquid nitrogen and stored at -80 °C until further use.

Expression and purification of GST-Cdc34, GST and GST-eGFP

Chemically competent *E. coli* Rosetta2 (DE3) were transformed with pGEX-6P-1-UBE2R1 (encoding for GST-Cdc34), pGEX-6P-1-GST or pGEX-6P-1-GST-eGFP (see Supplementary Table 3). After recovery with 1 mL of SOC medium for 1 h at 37 °C, the cells were cultured overnight in 50 mL of 2xYT medium containing ampicillin (100 µg/mL) and chloramphenicol (50 µg/mL) at 37 °C, 200 rpm. The overnight culture was diluted to an OD_{600} of 0.05 in 1 L of fresh 2xYT medium supplemented with ampicillin (50 µg/mL) and chloramphenicol (25 µg/mL) and cultured at 37 °C while shaking (200 rpm) to OD_{600} = 0.8. IPTG was added to a final concentration of 0.25 mM and protein expression was induced for 18 h at 20 °C. Cells were harvested by centrifugation (4000 x g, 20 min, 4 °C), resuspended in lysis buffer (50 mM Tris pH 8.0, 300 mM sucrose, 50 mM NaF, 2 mM DTT, 0.1 mg/mL DNase I and one cOmplete™ protease inhibitor tablet (Roche)), incubated on ice for 30 min and sonicated in an ice-water bath. Lysed cells were centrifuged (15 000 x g, 40 min, 4 °C), the cleared lysate added to Glutathione Sepharose 4B (GE Healthcare, 0.1 mL of slurry per 100 mL of culture) and incubated with agitation for 1 h at 4 °C. After incubation, the mixture was transferred to an empty plastic column and washed with 10 CV of wash buffer (25 mM Tris pH 8.5, 400 mM NaCl, 5 mM DTT). The protein was eluted in 1 mL fractions using elution buffer (wash buffer supplemented with 10 mM GSH pH 8.0). The fractions containing GST-Cdc34, GST or GST-eGFP were pooled and concentrated with Amicon® centrifugal filter units with a 10 kDa MWCO (Millipore) followed by SEC using a Superdex S75 16/600 (GE Healthcare) with SEC buffer (50 mM Tris pH 7.5, 150 mM NaCl and 1 mM DTT). Fractions containing GST-Cdc34, GST or GST-eGFP were again pooled and concentrated with Amicon® centrifugal filter units (Millipore). Protein concentration was calculated from the measured A280 absorption (extinction coefficients were calculated with ProtParam (https://web.expasy.org/protparam/)). GST-Cdc34, GST or GST-eGFP were flash frozen in liquid nitrogen and stored at -80 °C until further use.

Expression and purification of USP2

USP2 was expressed and purified as previously described.[5]

Staudinger reduction of AzGGisoK-bearing proteins

Reduction of AzGGisoK to GGisoK on POIs was either performed in cell lysate using 500 µM TCEP during protein purification or on purified protein using 2 eq. TCEP and incubation for 2 hours at 4 °C followed by rebuffering in an Amicon® centrifugal filter unit with appropriate size cutoff.

4. *Oa*AEP1 substrate scope and reversibility assays

Ub(NGL) was diluted to 100 µM in *Oa*AEP1 reaction buffer (100 mM Na_2HPO_4 / NaH_2PO_4 pH 7.4, 150 mM NaCl, 2 mM DTT) followed by the addition of 1 mM GVisoK/GLisoK/GGisoK (100 mM stock solutions in H_2O). Subsequently 2.5 µM *Oa*AEP1 were added to the reaction followed by incubation at 25 °C. Samples were taken at denoted time points by quenching the reaction mixture with 10 volumes of 0.1 % formic acid prior to HPLC-MS analysis.

For the reversibility assays, samples of Ub-NGXisoK were rebuffered to 20 mM MES pH 6.8 with 150 mM NaCl using Amicon® Ultra-0.5 3 kDa MWCO centrifugal filter units to remove any free GXisoK peptide. Concentration of rebuffered Ub-NGXisoK was determined using BCA assay (Thermo Scientific™). 20 µM Ub-NGXisoK were mixed with 0.5 µM *Oa*AEP1 in 20 mM MES pH 6.8 with 150 mM NaCl and incubated at 30 °C. Samples were taken at denoted time points by quenching the hydrolysis reaction with 10 volumes of 0.1 % formic acid prior to HPLC-MS analysis.

5. *Oa*AEP1-mediated labeling of GGisoK-bearing proteins

*Oa*AEP1-mediated labeling of Ub-K48GGisoK with dtb-NGL(H)

Ub-K48GGisoK-H_6 was diluted to 50 µM in *Oa*AEP1 reaction buffer (100 mM Na_2HPO_4 / NaH_2PO_4 pH 7.4, 150 mM NaCl, 2 mM DTT, ± 500 µM $NiSO_4$) followed by the addition of 1 mM dtb-NGL(H) (200 mM stock solutions in DMSO). Subsequently, 2.5 µM *Oa*AEP1 were added to the reaction followed by incubation at 25 °C. Samples were taken at denoted time points by quenching the reaction mixture with 10 volumes of 0.1 % formic acid prior to HPLC-MS analysis.

For control experiments Ub-K48BocK-H_6 or Ub-wt were used instead of Ub-K48GGisoK-H_6. Alterations of reaction conditions are stated in the corresponding Supplementary Figures.

*Oa*AEP1-mediated labeling of Ub-K48GGisoK-H_6 with alkyne-NGLH

Ub-K48GGisoK-H_6 was diluted to 50 µM in *Oa*AEP1 reaction buffer (50 mM MES pH 6.8, 100 mM NaCl, 1 mM $NiCl_2$) followed by the addition of 1 mM alkyne-NGLH (200 mM stock solution in H_2O). Subsequently, 5 µM *Oa*AEP1 were added to the reaction followed by incubation at 25 °C. Samples were taken at denoted time points by quenching the reaction mixture with 10 volumes of 0.1 % formic acid prior to HPLC-MS analysis. For control experiments Ub-K48BocK-H_6 was used instead of Ub-K48GGisoK-H_6.

*Oa*AEP1-mediated labeling of Ub-K48GGisoK-H_6 with SuRho-NGLH

Ub-K48GGisoK-H_6 was diluted to 50 µM in *Oa*AEP1 reaction buffer (100 mM Na_2HPO_4 / NaH_2PO_4 pH 7.4, 150 mM NaCl, 2 mM DTT, 1 mM $NiSO_4$) followed by the addition of 1 mM SuRho-NGLH (200 mM stock solution in DMSO). Subsequently, 10 µM *Oa*AEP1 were added to the reaction followed by incubation at 25 °C. Samples were taken at denoted time points by quenching the reaction mixture with 10 volumes of 0.1 % formic acid prior to HPLC-MS analysis. For control experiments Ub-K48BocK-H_6 was used instead of Ub-K48GGisoK-H_6.

*Oa*AEP1-mediated labeling of Ub-K48GGisoK-H_6 with Fmoc-$(G)_6$-NGLH

Ub-K48GGisoK-H_6 was diluted to 50 µM in *Oa*AEP1 reaction buffer (20 mM MES pH 6.8, 150 mM NaCl, 1 mM $NiCl_2$) followed by the addition of 1 mM Fmoc-$(G)_6$-NGLH (200 mM stock solution in DMSO). The reaction was started by the addition of 2 µM *Oa*AEP1 and incubation at 30 °C. Samples were taken at denoted time points by quenching the reaction mixture with 10 volumes 0.1 % formic acid (for LC-MS analysis) or one volume 3x SDS sample buffer and boiling at 95 °C for 10 min (for SDS-PAGE analysis). SDS samples were separated on 16.5 % acrylamide gels using a Tris/Tricine/SDS buffer system.

*Oa*AEP1-mediated labeling of eGFP-nanobody-H_6 with SuRho-NGLH

eGFP-nbxxxGGisoK-H_6 was diluted to 50 µM in *Oa*AEP1 reaction buffer (50 mM Tris pH 7.4, 150 mM NaCl, 1 mM $NiSO_4$) followed by the addition of 1 mM SuRho-NGLH (200 mM stock solution in DMSO). Subsequently 5 µM *Oa*AEP1 were added to the reaction followed by incubation at 25 °C. Samples were taken at the denoted time points and quenched by the addition of 4x SDS loading buffer and boiling at 95 °C for 10 min. Samples were separated *via* SDS-PAGE and in-gel fluorescence was captured using an iBright FL-1500 imaging system (Ex: 515 - 545 nm; Em: 568 - 617 nm) followed by Coomassie staining. For control experiments eGFP-nb-wt-H_6 was used instead of eGFP-nbxxxGGisoK-H_6.

Preparative *Oa*AEP1-mediated labeling of eGFP-nb-R75GGisoK-H_6 with SuRho-NGLH

eGFP-nb-R75GGisoK-H_6 was diluted to 30 µM in *Oa*AEP1 reaction buffer (50 mM Tris pH 7.4, 150 mM NaCl, 1 mM $NiSO_4$) followed by addition of 600 µM SuRho-NGLH (200 mM stock solution in DMSO). Subsequently, 0.5 µM *Oa*AEP1 were added to the reaction followed by incubation at 25 °C. Every 30 min 0.5 µM *Oa*AEP1 were additionally spiked in over a 3 h period. Reaction progress was monitored by HPLC-MS. Once labeling efficiency ≥ 90 % was observed, 15 µM N-Acetyl-Tyr-Val-Ala-Asp chloromethyl ketone were added to the reaction followed by incubation for 10 min at 30 °C. The reaction mixture was diluted 1:20 into Ni-NTA wash buffer (50 mM Tris pH 7.4, 150 mM NaCl, 10 mM imidazole), added to Ni-NTA slurry (Jena Bioscience) and incubated with agitation for 1 h at 4 °C. The mixture was then transferred to an empty plastic column and washed with 10 CV of wash buffer (50 mM Tris pH 7.4, 150 mM NaCl and 10 mM imidazole). The protein was eluted with wash buffer supplemented with 300 mM imidazole pH 8.0 and concentrated using Amicon® centrifugal filter units with a 3 kDa MWCO (Millipore).

Samples were taken at denoted time points and quenched by the addition of 4x SDS loading buffer and boiling at 95 °C for 10 min. Samples were separated *via* SDS-PAGE and in-gel fluorescence was captured using an iBright FL-1500 imaging system (Ex: 515 - 545 nm; Em: 568 - 617 nm) followed by Coomassie staining.

*Oa*AEP1-mediated double labeling of TEV-GV-Ub-K48GGisoK-H_6 with dtb-NGLH and alkyne-NGLH

TEV-GV-Ub-K48GGisoK-H_6 was diluted to 50 µM in *Oa*AEP1 reaction buffer (20 mM MES pH 6.8, 150 mM NaCl, 500 µM $NiCl_2$) and 1 mM dtb-NGLH as well as 2 µM *Oa*AEP1 were added to the solution. The reaction was carried out at 30 °C and reaction progress was monitored *via* HPLC-MS. Once a turnover of ~90 % was observed, the reaction was quenched by addition of 3 volumes 50 mM Tris pH 8.0 with 150 mM NaCl. Single-labeled TEV-GV-Ub-K48(dtb-N)GGisoK-H_6 was then purified on Ni-NTA magnetic beads (Cube Biotech; using 1.5 eq. binding capacity compared to the amount of starting material) by binding for 2 h at 4 °C while rotating and washing 3 times with wash buffer (50 mM Tris pH 8.0 with 150 mM NaCl) for 5 min each. Protein was eluted from beads using 300 mM imidazole pH 8.0 and then further diluted with 3 volumes *Oa*AEP1 reaction buffer. 0.2 U/µl TEV protease were added to the solution and cleavage was carried out for 60 min at 30 °C. Complete cleavage of TEV sites was confirmed *via* HPLC-MS. TEV-cleaved GV-Ub-K48(dtb-N)GGisoK-H_6 was then labeled with

1 mM alkyne-NGLH using 2 µM *Oa*AEP1 at 30 °C while reaction progress was monitored *via* HPLC-MS. Once >90 % labeling efficiency was observed, the reaction was quenched and double-labeled alkyne-NGV-Ub-K48(dtb-N)GGisoK-H_6 was again purified using Ni-NTA magnetic beads as described above. Collected samples from different stages of the procedure were separated using SDS-PAGE and visualized by Coomassie staining.

On-bead CuAAC of double-labeled alkyne-NGV-Ub-K48(dtb-N)GGisoK-H_6 with Picolyl-Azide-Sulfo-Cy5

20 µM alkyne-NGV-Ub-K48(dtb-N)GGisoK-H_6 were bound to Ni-NTA magnetic beads (Cube Biotech; using 1.5 eq. binding capacity compared to the amount of starting material) for 2 h at 4 °C while rotating. Beads were washed 3 times with PBS for 5 min each. 100 µM Picolyl-Azide-Sulfo-Cy5 (Jena Bioscience) with 50 µM $CuSO_4$, 250 µM THPTA and 2.5 mM freshly dissolved ascorbic acid in PBS were added to bead-bound alkyne-NGV-Ub-K48(dtb-N)GGisoK-H_6, followed by 30 min incubation at RT while rotating. The reaction mixture was removed and beads were subjected to washing until Sulfo-Cy5 dye was not visible in washes anymore. Protein was eluted using 300 mM imidazole pH 8.0 and success of CuAAC was confirmed *via* HPLC-MS. Samples of CuAAC flow-through and labeled protein were separated using SDS-PAGE and visualized by in-gel fluorescence and Coomassie staining.

6. *Oa*AEP1-mediated Ublyation of GGisoK-bearing proteins

*Oa*AEP1-mediated ubiquitylation of Ub-K48GGisoK-H_6, SUMO-K11GGisoK-H_6 and H3-K23/27/79GGisoK-H_6

Target proteins were diluted into *Oa*AEP1 reaction buffer (conditions denoted in main and supplemental figures and associated figure legends) followed by the addition of Ub(N). Subsequently, *Oa*AEP1 was added to the reaction followed by incubation at 25/30 °C. Samples were taken at denoted time points and quenched by the addition of 4x SDS loading buffer and boiling at 95 °C for 10 min. Samples were separated using SDS-PAGE and visualized by Coomassie staining.

*Oa*AEP1-mediated SUMOylation of Ub-K48GGisoK-H_6

Ub-K48GGisoK-H_6 was diluted to 150 µM in *Oa*AEP1 reaction buffer (100 mM Na_2HPO_4 / NaH_2PO_4 pH 7.4, 150 mM NaCl, 1 mM TCEP) followed by the addition of 150 µM SUMO2(N)-H_6. Subsequently, 2 µM *Oa*AEP1 were added to the reaction followed by incubation at 25 °C. Samples were taken at denoted time points and quenched by the addition

of 4x SDS loading buffer and boiling at 95 °C for 10 min. Samples were separated using SDS–PAGE and visualized by Coomassie staining. For control experiments Ub-K48BocK-H_6 was used instead of Ub-K48GGisoK- H_6.

7. Preparation of K48-diUbs and DUB assays

Preparation of natively linked K48-diUb (K48-diUb-wt)

Assembly of natively linked K48-diUb was carried out as previously described with slight adjustments.[12] The assembly reaction contained 50 nM H_6-UBE1 (Boston Biochem, Cat. No. E-304-050), 4.5 µM GST-Cdc34 and 1 mM of Ub-wt. After incubation at 37 °C for 16 h in diUb reaction buffer (50 mM Tris pH 7.5, 10 mM $MgCl_2$, 0.6 mM DTT and 10 mM ATP) the reaction was diluted in cation exchange buffer (1:20 in 50 mM Ammonium acetate pH 4.5) in order to precipitate enzymes. Afterwards, the solution was filtered and the assembled K48-diUbs were purified by cation exchange using a Resource S column (GE Healthcare, gradient 0 - 500 mM NaCl). Fractions containing pure K48-diUb were pooled and concentrated with Amicon® 3 kDa MWCO centrifugal filter units (Millipore). K48-diUb was stored at -80 °C until further use.

Preparation of K48-diUb(AT) using sortase

Ub-K48GGisoK-H_6 acceptor was diluted to 20 µM in sortase buffer (50 mM Tris pH 7.5, 150 mM NaCl, 5 mM $CaCl_2$). 100 µM of the donor ubiquitin (Ub(AT)) were added, followed by the addition of 20 µM Srt2A (without His_6-Tag). Incubation was performed at 37 °C, 600 rpm for 18 h. Sortase-mediated transpeptidation was stopped by the addition of 200 µM phenyl vinyl sulfone and further incubation for 10 min at 37 °C, 600 rpm. Ni-NTA slurry (Jena Bioscience, 0.1 mL/mg of acceptor ubiquitin) was added to the reaction mixture followed by incubation while shaking for 1 h at 4 °C. After incubation, the mixture was transferred to an empty plastic column and washed with 40 CV of wash buffer (50 mM Tris pH 7.5, 150 mM NaCl, 5 mM $CaCl_2$, 30 mM imidazole) to remove Srt2A and the excess of donor ubiquitin. The protein was eluted in 0.2 mL fractions using wash buffer supplemented with 300 mM imidazole pH 8.0. The fractions containing the mixture of K48-diUb and unreacted Ub-K48GGisoK acceptor ubiquitin were pooled and concentrated *via* Amicon® with the corresponding MWCO centrifugal filter units (Millipore). In order to remove unreacted acceptor ubiquitin, size-exclusion chromatography (SEC) was performed using a Superdex S75 16/600 (GE Healthcare) with 50 mM Tris pH 7.5, 150 mM NaCl. Fractions containing the K48-diUb "AT" were pooled

and concentrated *via* Amicon® with the corresponding MWCO centrifugal filter units (Millipore). DiUbs were flash frozen in liquid nitrogen and stored at -80 °C until further use.

Preparation of K48-diUb(N) using *Oa*AEP1

Ub-K48GGisoK-H_6 acceptor Ub was diluted to 50 µM in 50 mM MES pH 6.8, 100 mM NaCl, 500 µM $NiCl_2$. 250 µM of the donor ubiquitin (Ub(NGLH)) and 1 µM *Oa*AEP1 were added and the mixture was incubated at 25 °C for 2 h. *Oa*AEP1-mediated transpeptidation was stopped by the addition of 10 µM N-Acetyl-Tyr-Val-Ala-Asp chloromethyl ketone and further incubation for 10 min at 25 °C. Ni-NTA slurry (Jena Bioscience, 0.1 mL/mg of acceptor ubiquitin) was then added to the reaction and incubation while agitating for 1 h at 4 °C followed. The mixture was transferred to an empty plastic column and washed with 40 CV of wash buffer (50 mM Tris pH 7.5, 150 mM NaCl, 30 mM imidazole) to remove *Oa*AEP1 and the excess of donor ubiquitin. The protein was eluted in 0.2 mL fractions using wash buffer supplemented with 300 mM imidazole pH 8.0. Fractions containing the mixture of K48-diUb and unreacted Ub-K48GGisoK acceptor ubiquitin were pooled and concentrated *via* Amicon® with the corresponding MWCO centrifugal filter units (Millipore). In order to remove unreacted acceptor ubiquitin, size-exclusion chromatography (SEC) was performed using a Superdex S75 16/600 (GE Healthcare) with 25 mM Tris pH 7.4, 100 mM NaCl. Fractions containing the K48-diUb were pooled and concentrated *via* Amicon® with the corresponding MWCO centrifugal filter units (Millipore). K48-diUb "N" was flash frozen in liquid nitrogen and stored at -80 °C until further use.

DUB Assays

USP2 was diluted into DUB reaction buffer (25 mM Tris pH 7.5, 150 mM NaCl, 10 mM DTT) and activated at RT for 10 min. Afterwards, 200 nM of USP2 were added to 10 µM K48-diUb variants. At denoted time points, 8 µL samples were taken and quenched by the addition of 12 µL 2x SDS loading buffer. After boiling at 95 °C for 10 min, samples were separated by SDS–PAGE and visualized by Coomassie staining.

8. Pull-down assays

Pull-down assays with GST-eGFP

Pull-down assays were performed as described previously.[6] 10 µg of GST or GST-eGFP were incubated with 30 µL 50 % slurry of Glutathione Sepharose 4B (GE Healthcare) preequilibrated with pull-down buffer (50 mM Tris pH 7.5, 150 mM NaCl, 1 mM TCEP and 0.1 % NP-40) for

1 h at 4 °C. Subsequently, beads were washed 4 times with pull-down buffer (4000 x g, 2 min, 4 °C) followed by addition of 10 µM of the corresponding eGFP-nanobodies variants (either wt or GGisoK-bearing) in a total volume of 50 µL. After incubation at 4 °C for 1 h the beads were washed 5 times with 100 µL pull down-buffer followed by the addition of 50 µL 1x SDS loading buffer and SDS-PAGE analysis.

9. Mammalian cell culture

HEK293T cells were maintained in Dulbecco's Modified Eagle's Medium (DMEM – high glucose, Sigma Aldrich) supplemented with 10 % (v/v) fetal bovine serum (FBS, Biochrom) and 1 % antibiotic-antimycotic solution (Sigma Aldrich) at 37 °C and 5 % CO_2 atmosphere in a humidified chamber.

<u>Seeding and transfection for confocal imaging</u>

50.000 HEK293T cells were transfected in reverse using 60 ng plasmid and 200 ng PEI (Sigma Aldrich) when seeding in an ibidi µ-slide VI 0.4 channel with a volume of 30 µl. 3 h after initial seeding, both wells of the channel were filled with 60 µl cell medium each.

<u>Preparation for fixed cell imaging</u>

Cells were fixed by glyoxal fixation 48 h post transfection. Therefore, cells were washed twice with PBS before 60 µl freshly made fixation mix (3 % (v/v) glyoxal, 20 % (v/v) ethanol and 0.75 % (v/v) acetic acid in H_2O with pH 4-5, adjusted with 1 M NaOH) were added to each channel. After incubation for 30 min on ice and 30 min at RT the fixation was quenched by addition of 60 µl 100 mM ammonium chloride and incubation at RT for another 20 min. Subsequently, cells were washed with PBS and incubated with 300 nM of either eGFP-nb-R75(SuRho-N)GGisoK-H_6, eGFP-nb-R75GGisoK-H_6 or SuRho-NGLH in PBS with 1 µg/ml DAPI for 30 min at 37 °C. Cells were then washed 3 times with PBS before image acquisition.

<u>Preparation for live cell imaging</u>

48 h post transfection cells were washed with PBS and then incubated with 300 nM of either eGFP-nb-R75(SuRho-N)GGisoK-H_6, eGFP-nb-R75GGisoK-H_6 or SuRho-NGLH in PBS for 30 min at 37 °C. Cells were washed another 3 times before proceeding with image acquisition.

10. Confocal microscopy

Confocal microscopy was performed on an SP8 Stellaris 8 Falcon system (Leica Microsystems) with an HC PL CS2 63x/1.4 oil objective. The fluorophores were imaged sequentially and with acousto-optic tunable filters set to ensure no crosstalk between the fluorophores. Between each channel of the ibidi slide the laser intensities of the respective fluorophore were kept constant. DAPI was excited at 405 nm, eGFP at 489 nm and Sulfo-Rhodamine at 543 nm. Image analysis was then performed using FIJI ImageJ.[7]

11. LC-MS

LC-MS was carried out on an Agilent Technologies 1260 Infinity LC-MS system with a 6310 Quadrupole spectrometer. The solvent system consisted of 0.1 % formic acid in water as solvent A and 0.1 % formic acid in ACN as solvent B.

Proteins were separated on a Phenomenex Jupiter C4 300 A LC Column (150 x 2 mm, 5 µm) using a gradient from 10 % to 55 % B in 1.65 min followed by a gradient from 55 % to 90 % in 0.85 min at a flow rate of 0.9 mL/min. The protein samples were analyzed in positive mode as well as by UV absorbance at 193, 254 and 280 nm. Deconvolution was performed with the Agilent OpenLAB CDS ChemStation LC/MS software using standard parameters.

Small molecules were separated on a Phenomenex Luna C18 100 A LC Column (100 x 2 mm, 2.5 µm) using a gradient from 15 % to 95 % B in 3.6 min at a flow rate of 0.38 mL/min. Samples were analyzed in positive mode, as well as by UV absorbance at 193, 254 and 280 nm.

<u>Evaluation of LC-MS data integrity</u>

Ub-K48GGisoK-H_6 was labeled with dtb-NGLH as described above. To obtain pure Ub-K48(dtb-N)GGisoK, the protein was first cleaned up from excess dtb-NGLH on NiNTA magnetic beads as described above and then further purified to homogeneity on a StrepTrap™ HP 1 ml column using 50 mM Tris-HCl pH 8.0 with 150 mM NaCl. Ub-K48(dtb-N)GGisoK-H_6 was eluted in 200 µl fractions in elution buffer (50 mM Tris-HCl pH 8.0, 150 mM NaCl, 2.5 mM desthiobiotin). Purity was confirmed via LC-MS.

Protein concentrations of aliquots of pure Ub-K48GGisoK-H_6 and pure Ub-K48(dtb-N)GGisoK-H_6 were determined via BCA assay (Thermo Scientific). Equal amounts of proteins were loaded onto an SDS-PAGE gel and coomassie staining confirmed similar protein concentrations. Both proteins were mixed in varying ratios (0.25:0.75; 0.5:0.5; 0.75:0.25) and injected into the LC-MS.

Author contributions:

K.L. and M.F. conceived the research plan and experimental strategy. M.F. synthesized peptides, performed cloning, expression and purification of proteins, labeling and ubiquitylation/SUMOylation assays. J.H. performed cloning, protein expression and purification, and performed *Oa*AEP1 hydrolysis and dual labeling experiments. M.G. established *Oa*AEP1 expression and purification, performed cloning, expression and purification of proteins and established initial *Oa*AEP1-mediated transpeptidation and ubiquitylation on GGK and GGK-POIs. R.M. performed protein expression and purification and performed preparative nanobody labeling and confocal microscopy with help from T.N. All authors analyzed data, K.L., M.F and J.H. wrote the paper with input from the other authors. All authors have given approval to the final version of the manuscript.

References

(1) Chiu, J.; March, P. E.; Lee, R.; Tillett, D. Site-directed, Ligase-Independent Mutagenesis (SLIM): a single-tube methodology approaching 100% efficiency in 4 h. *Nucleic Acids Res.* **2004**, *32* (21). DOI: ARTN e174
10.1093/nar/gnh172.
(2) Fottner, M.; Brunner, A. D.; Bittl, V.; Horn-Ghetko, D.; Jussupow, A.; Kaila, V. R. I.; Bremm, A.; Lang, K. Site-specific ubiquitylation and SUMOylation using genetic-code expansion and sortase. *Nat. Chem. Biol.* **2019**, *15* (3), 276-+. DOI: 10.1038/s41589-019-0227-4.
(3) Hammill, J. T.; Miyake-Stoner, S.; Hazen, J. L.; Jackson, J. C.; Mehl, R. A. Preparation of site-specifically labeled fluorinated proteins for F-19-NMR structural characterization. *Nat. Protoc.* **2007**, *2* (10), 2601-2607. DOI: 10.1038/nprot.2007.379.
(4) Yang, R. L.; Wong, Y. H.; Nguyen, G. K. T.; Tam, J. P.; Lescar, J.; Wu, B. Engineering a Catalytically Efficient Recombinant Protein Ligase. *J. Am. Chem. Soc.* **2017**, *139* (15), 5351-5358. DOI: 10.1021/jacs.6b12637.
(5) Renatus, M.; Parrado, S. G.; D'Arcy, A.; Eidhoff, U.; Gerhartz, B.; Hassiepen, U.; Pierrat, B.; Riedl, R.; Vinzenz, D.; Worpenberg, S.; et al. Structural basis of ubiquitin recognition by the deubiquitinating protease USP2. *Structure* **2006**, *14* (8), 1293-1302. DOI: 10.1016/j.str.2006.06.012.
(6) Komander, D.; Reyes-Turcu, F.; Licchesi, J. D. F.; Odenwaelder, P.; Wilkinson, K. D.; Barford, D. Molecular discrimination of structurally equivalent Lys 63-linked and linear polyubiquitin chains (vol 10, pg 466, 2009). *EMBO Rep.* **2009**, *10* (6), 662-662. DOI: 10.1038/embor.2009.106.
(7) Schindelin, J.; Arganda-Carreras, I.; Frise, E.; Kaynig, V.; Longair, M.; Pietzsch, T.; Preibisch, S.; Rueden, C.; Saalfeld, S.; Schmid, B.; et al. Fiji: an open-source platform for biological-image analysis. *Nat. Methods* **2012**, *9* (7), 676-682. DOI: 10.1038/nmeth.2019.
